# Nine dietary habits and risk of colorectal cancer: a Mendelian randomization study

**DOI:** 10.1186/s12920-023-01782-7

**Published:** 2024-01-17

**Authors:** Mengyang He, Luyao Huan, Xuan Wang, Yingyi Fan, Jinchang Huang

**Affiliations:** 1 Graduate School, Beijing University of Chinese Medicine, Beijing, China; 2 Xiyuan Hospital of China Academy of Chinese Medical Sciences, Beijing, China; 3 Beijing University of Chinese Medicine Third Affiliated Hospital, No. 51 Anwai Xiaoguan Street, Chaoyang District, Beijing, 100029 China

**Keywords:** Causality, Dietary, Mendelian randomization, Colorectal cancer

## Abstract

**Background:**

Epidemiological studies have provided evidence that there is an association between diet and colorectal cancer. However, the causal relationship between dietary habits and colorectal cancer remains unknown.

**Methods:**

The UK Biobank provided summary-level genome-wide association study data for nine dietary habits, including alcohol consumption (n = 549,703), instant coffee consumption (n = 250,308), fruit consumption (n = 210,947), meat consumption (n = 210,947), full cream milk consumption (n = 41,306), sweets consumption (n = 25,521), tea consumption (n = 501,494), vegetable consumption (n = 210,947), and yogurt/ice cream consumption (n = 210,947). Additionally, data on colorectal cancer were collected, consisting of 5,567 cases and 372,016 controls. The MR analysis employed inverse variance weighted, weighted median, MR-Egger regression, and MR multivariate residuals tests.

**Results:**

In the predominantly European population, a positive association was observed between vegetables (OR = 1.014, 95% CI = 1.000-1.029, p = 0.048) and an increased risk of colorectal cancer. The results for vegetable did not survive correction for multiple comparisons. However, no strong evidence was found for other dietary factors, such as alcohol (OR = 1.012, 95% CI = 0.974–1.051, p = 0.556), fruit (OR = 1.007, 95% CI = 0.986–1.029, p = 0.512), meat (OR = 1.000, 95% CI = 0.987–1.026, p = 0.968), full cream milk (OR = 1.019, 95% CI = 0.979–1.061, p = 0.357), sweets (OR = 0.998, 95% CI = 0.991–1.004, p = 0.524), and tea (OR = 1.002, 95% CI = 0.994–1.009, p = 0.672), with regards to colorectal cancer risk in the European population.

**Conclusions:**

Our study highlights the need for a more nuanced approach to dietary recommendations for CRC prevention, with greater emphasis adherence to the Mediterranean dietary pattern.

**Supplementary Information:**

The online version contains supplementary material available at 10.1186/s12920-023-01782-7.

## Introduction

Colorectal cancer (CRC) is a prevalent malignant tumor globally, ranking third in frequency and second in mortality after lung cancer. In 2020, more than 1.9 million new cases and 935,000 deaths were reported [1], and if the current trend persists, the burden of CRC will surge by 60% to over 2.2 million cases and 1.1 million deaths annually by 2030 [2]. Although lifestyle factors and metabolic conditions like smoking, physical inactivity, sedentary behavior, and diabetes mellitus have been linked to an increased incidence and mortality of CRC, the exact cause of the disease remains unclear [3–8]. Consequently, CRC is a multifactorial condition involving many potential etiological factors, emphasizing the need for identifying risk factors to aid in its prevention.

Over the past few decades, numerous epidemiological studies have established a correlation between specific dietary patterns and the risk of colorectal cancer (CRC). According to the Global Burden of Disease study (GBD) 2019, dietary factors are considered to be one of the most critical factors impacting the prognosis of CRC. Dietary compounds have the potential to influence CRC in various ways [9, 10]. For example, a meta-analysis of 13 prospective cohort studies conducted by Zhong et al. [11] in 2020 revealed that compliance with a Mediterranean diet was associated with a 10% reduction in CRC incidence. Similarly, Bradbury et al. (2019) [12] found that individuals who consumed an average of 76 g of red and processed meats daily had a 20% higher risk of CRC than those who consumed only 21 g daily. While some studies have reported positive results for a vegetarian diet in preventing and treating CRC [13, 14], others have produced conflicting results [15–17]. Therefore, it remains unclear whether there are any causal associations between dietary habits and CRC risk.

Mendelian randomization (MR) is a genetically informed methodology that utilizes single-nucleotide polymorphisms (SNPs) as instrumental variables (IVs) for risk factors of interest. This approach provides a valid way to assess causality free from confounding or reverse causality bias [18]. Unlike randomized controlled trials (RCTs) [19], MR allows investigation of many exposures that cannot be studied using RCTs. However, to date, no MR studies have explored the potential causal relationships between dietary habits and colorectal cancer (CRC) risk.

The aim of this study is to examine the potential causal associations between nine dietary habits (vegetable consumers, alcohol consumption, instant coffee consumption, tea consumption, milk consumption, yoghurt consumption, meat consumption, fruit consumption, and sweets consumption) and CRC risk using MR analyses.

## Methods

### Genetic variants associated with 9 dietary habits

This MR study is reported according to the reporting guidelines for enhancing observational epidemiological studies using MR (STROBE-MR). The data for this study were obtained from UK Biobank (https://www.nealelab.is/uk-biobank) and are publicly available without access restrictions. To increase the number of single nucleotide polymorphisms (SNPs) related to dietary habits, a more relaxed threshold (p < 5 × 10^− 6^) was used, and the chain imbalance was set to LD (r^2^ < 0.001) to ensure study robustness. An F-statistic for SNPs greater than the conventional value of 10 was used to assess the potential of the tool to predict instrumental variable [20].

The exposure instrumental variable was assessed using nine genome-wide association studies (GWAS) based on UK Biobank data, which examined the association between SNPs and vegetable consumption (n = 210,947), vegetables (female), vegetables (male) consumption, fruit consumption (n = 210,947), beef intake consumption (n = 69,687), tea consumption (n = 501,494), meat consumption (n = 210,947), sweets consumption (n = 25,521), decaffeinated coffee consumption (n = 88,784), ground coffee consumption (n = 115,952), instant coffee consumption (n = 250,308), other type of coffee consumption (n = 8,754), full cream milk consumption (n = 41,603), skimmed milk consumption (n = 119,480), semi-skimmed milk consumption (n = 382,990), soya milk consumption (n = 22,717), other type of milk consumption (n = 9,933) and yogurt/ice cream consumption (n = 210,947).

To provide a more detailed analysis of the types of alcohol consumption, we included ten phenotypes related to alcohol consumption, including alcohol (female), alcohol (male), alcohol intake frequency (n = 501,494), alcohol drinker status (current) (n = 549,703), alcohol drinker status (previous) (n = 21,317), alcohol drinker status (never) (n = 25,396), red wine consumption (n = 60,887), rose wine consumption (n = 11,404), white wine consumption (n = 48,877), and fortified wine consumption (n = 7,585). Using a touch-screen tablet, the participants filled in the prepared relevant questionnaire, from which the instrumental variables were obtained. This resource may be accessed at: https://biobank.ndph.ox.ac.uk/showcase/ukb/docs/TouchscreenQuestionsMainFinal.pdf.

## GWAS summary data for CRC

UK Biobank Cohort Study’s GWAS yielded overall cancer risk data for 5,567 cases and 372,016 controls [21]. Briefly, cancer cases were categorized according to ICD-9 (http://www.icd9data.com/2007/Volume1/default.htm) and ICD-10 (https://icd.who.int/browse10/2016/en), with data completed through September 2019, and controls were defined as individuals without any cancer code (ICD10 or ICD2) and without a self-reported cancer diagnosis. More information on estimation and quality control measures can be found in other topics [22]. We retrieved the data from the IEU OPEN GWAS PROJECT and extracted the single nucleotide polymorphisms (SNPs) associated with various dietary habits, along with their effect sizes and standard errors. Any SNPs with intermediate allele frequency were removed from the analysis. Detailed information on the SNPs associated with each dietary habit and their association with CRC can be found in Supplementary Table 1.

**Table 1:** Mendelian randomization estimates of the associations between dietary habits and colorectal cancer

Exposure	Outcome	nSNP	IVW-derived P value	OR (95%CI)	MR-Egger- derived P value	OR (95%CI)	WM-derived P value	OR (95%CI)	Egger intercept-derived P value
Vegetables	Colorectal cancer	12	0.048	1.014 (1.000-1.029)	0.819	1.004 (0.974-1.034)	0.045	1.019 (1.000-1.038)	0.438
Vegetables (Female)	Colorectal cancer	6	0.788	1.003 (0.981-1.026)	0.702	1.013 (0.953-1.076)	0.644	1.005 (0.984-1.027)	0.75
Vegetables (Male)	Colorectal cancer	9	0.32	1.006 (0.995-1.017)	0.978	1.001 (0.962-1.041)	0.916	0.999 (0.986-1.013)	0.804
Fruit	Colorectal cancer	8	0.512	1.007(0.986-1.029)	0.95	1.002 (0.932-1.078)	0.327	1.019 (0.984-1.055)	0.896
Beef intake	Colorectal cancer	5	0.692	1.002 (0.992-1.013)	0.731	1.004 (0.985-1.022)	0.821	1.002 (0.988-1.015)	0.865
Tea	Colorectal cancer	23	0.672	1.002 (0.994-1.009)	0.418	1.008 (0.990-1.026)	0.963	1.000 (0.991-1.010)	0.482
Meat	Colorectal cancer	9	0.968	1.000 (0.987-1.013)	0.683	0.993 (0.961-1.026)	0.982	1.000 (0.984-1.017)	0.645
Sweets	Colorectal cancer	18	0.524	0.998 (0.991-1.004)	0.891	1.001 (0.989-1.013)	0.59	0.998 (0.990-1.006)	0.547
Alcohol									
Alcohol drinker status (current)	Colorectal cancer	27	0.556	1.012 (0.974-1.051)	0.427	0.961 (0.873-1.058)	0.726	1.009 (0.960-1.061)	0.268
Alcohol drinker status (Never)	Colorectal cancer	23	0.715	1.010 (0.957-1.067)	0.543	0.965 (0.862-1.080)	0.73	0.987 (0.915-1.065)	0.374
Alcohol drinker status (Previous)	Colorectal cancer	20	0.971	1.001 (0.935-1.072)	0.058	0.877 (0.773-0.996)	0.806	1.011 (0.929-1.100)	0.312
Alcohol (Female)	Colorectal cancer	12	0.184	1.004 (0.998-1.011)	0.824	1.002 (0.984-1.020)	0.257	1.004 (0.997-1.011)	0.798
Alcohol (Male)	Colorectal cancer	9	0.87	1.001 (0.993-1.008)	0.912	1.001 (0.983-1.020)	0.085	1.006 (0.999-1.012)	0.958
Alcohol intake frequency	Colorectal cancer	184	0.154	0.999 (0.997-1.001)	0.178	0.997 (0.992-1.001)	0.12	0.997 (0.993-1.001)	0.404
White wine	Colorectal cancer	20	0.708	1.001 (0.997-1.005)	0.38	1.004 (0.996-1.012)	0.617	1.001 (0.996-1.006)	0.422
Fortified wine	Colorectal cancer	16	0.764	0.997 (0.981-1.014)	0.949	0.999 (0.971-1.028)	0.747	0.996 (0.975-1.018)	0.894
Red wine	Colorectal cancer	14	0.893	1.000 (0.997-1.002)	0.803	1.000 (0.997-1.003)	0.897	1.000 (0.997-1.003)	0.804
Coffee									
Decaffeinated coffee	Colorectal cancer	14	0.104	1.025 (0.995-1.005)	0.671	0.985 (0.921-1.054)	0.109	1.033 (0.993-1.075)	0.227
Ground coffee	Colorectal cancer	91	0.266	0.994 (0.983-1.005)	0.677	1.009 (0.969-1.050)	0.35	0.993 (0.978-1.0008)	0.456
Instant coffee	Colorectal cancer	33	0.435	0.994 (0.978-1.010)	0.666	1.012 (0.960-1.067)	0.338	0.990 (0.970-1.010)	0.484
Other type of coffee	Colorectal cancer	5	0.997	1.000 (0.869-1.150)	0.25	1.212 (0.930-1.579)	0.838	1.019 (0.850-1.220)	0.192
Milk									
Full cream milk	Colorectal cancer	22	0.357	1.019 (0.979-1.061)	0.086	1.117 (0.991-1.259)	0.809	1.007 (0.950-1.067)	0.127
Skimmed milk	Colorectal cancer	29	0.608	0.994 (0.970-1.018)	0.674	0.982 (0.903-1.068)	0.679	0.994 (0.964-1.024)	0.775
Semi-skimmed milk	Colorectal cancer	12	0.662	0.994 (0.965-1.023)	0.995	1.000 (0.922-1.083)	0.974	1.001 (0.9639-1.03)	0.876
Soya milk	Colorectal cancer	23	0.597	0.983 (0.923-1.047)	0.886	0.987 (0.832-1.172)	0.329	0.960 (0.886-1.042)	0.956
Other type of milk	Colorectal cancer	12	0.126	0.893 (0.773-1.032)	0.459	0.894 (0.671-1.190)	0.414	0.925 (0.768-1.115)	0.998
Rose wine	Colorectal cancer	23	–	–	–	–	–	–	0.013
Yogurt/Ice-cream	Colorectal cancer	14	–	–	–	–	–	–	0.031

*Abbreviation*: SNP, single-nucleotide polymorphism; IVW, inverse variance weighted; WM, weighted median. Due to the large directional pleiotropy (P<0.05) for the instrumental variables of yogurt/ice cream and rose wine, we considered further analysis of the causal association with CRC to be of little significance

### Statistical analyses

After obtaining GWAS summary data for different dietary habits and CRC from UK Biobank, we employed various MR methods to determine MR estimates for the different dietary habits in CRC, including inverse variance weighted (IVW), weighted median, and MR-Egger. Since these methods have different underlying assumptions regarding horizontal pleiotropy, using multiple methods helped increase the robustness of our results. Our main result was based on an inverse variance-weighted meta-analysis of Wald ratios for individual SNPs, assuming that the instrument could only influence the results through exposure of interest and not through any alternative pathway [23].

To complement our IVW estimates, we also used MR-Egger and weighted median methods. While these methods are less efficient and have wider confidence intervals, they can provide more robust estimates across a wider range of scenarios. Sensitivity analysis played a crucial role in our MR study, allowing us to detect potential pleiotropy. We used a heterogeneity marker (Cochran Q-derived p < 0.05) from the IVW method to indicate potential horizontal pleiotropy, while the intercept obtained from MR-Egger regression indicated directional pleiotropy (p < 0.05 was considered evidence of directional pleiotropy) [24]. We also used the Bonferroni multiple correction method.

**Fig. 1:**
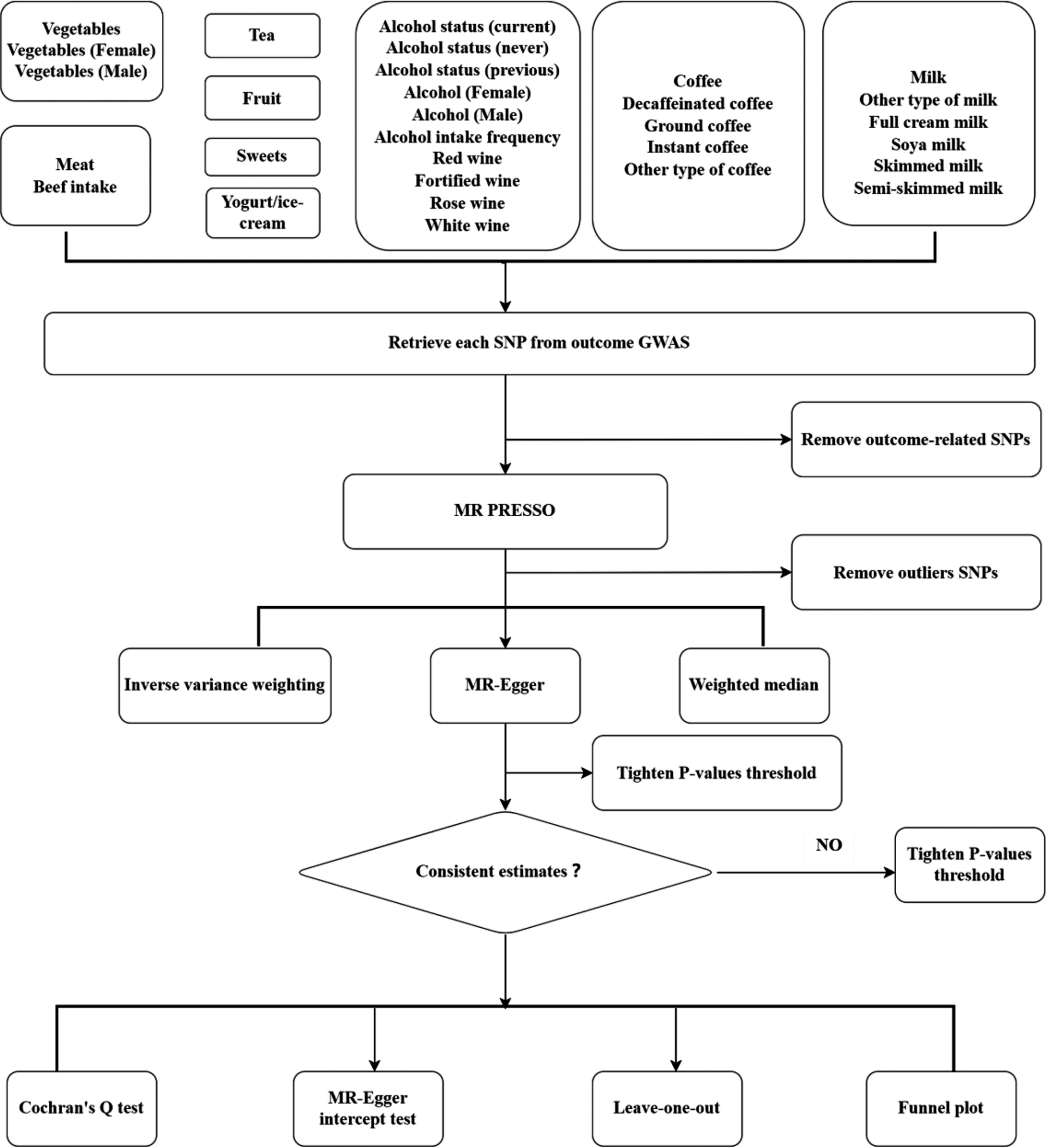
Study flame chart of the Mendelian randomization study revealing the causal relationship between dietary habits and colorectal cancer

To assess whether MR estimates were driven or biased by individual SNPs, we performed leave-one-out analysis. All analyses were performed using Two-Sample MR in R (version 4.2.2) The study frame chart is presented in Fig. 1.

## Results

### Causal effect from 9 dietary habits to CRC

**Fig. 2:**
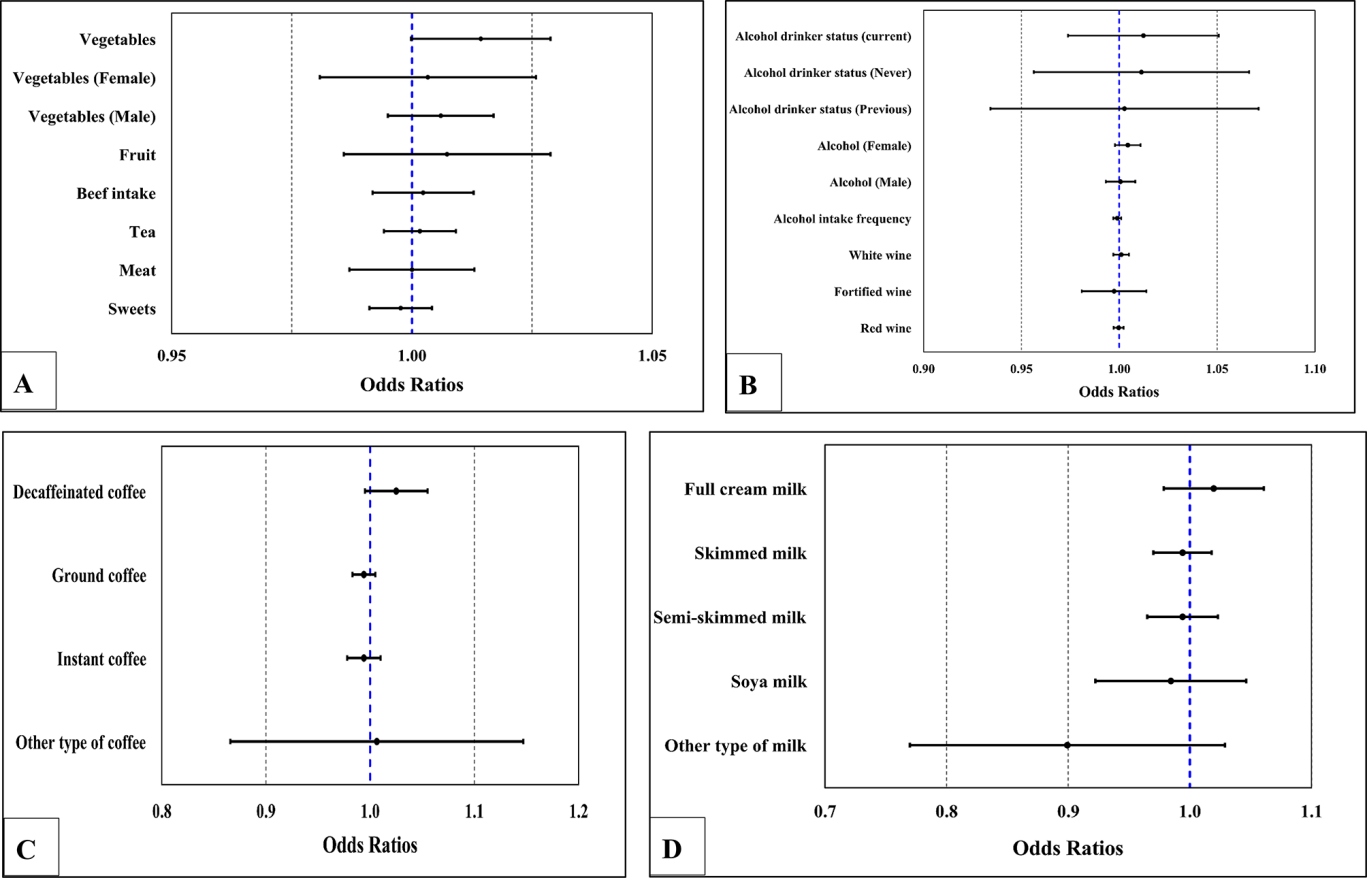
(**A**) Odds ratio plot for dietary habits and colorectal cancer. (**B**) Odds ratio plot for dietary habits of alcohol and colorectal cancer. (**C**) Odds ratio plot for dietary habits of coffee and colorectal cancer. (**D**) Odds ratio plot for dietary habits of milk and colorectal cancer

Using an IVW random effects model with 12 SNPs associated with vegetable consumers, we found a potentially causal effect of vegetable consumers on CRC risk that was significant (OR = 1.014, 95% CI = 1.000-1.029, p = 0.048) (Fig. 2A). Meanwhile, similar risk estimates were obtained using the weighted median (WM) method (OR = 1.019, 95% CI = 1.000-1.038, p = 0.045) (Table 1). The results for vegetable did not survive correction for multiple comparisons (The corrected p-value was 0.0028).

**Fig. 3:**
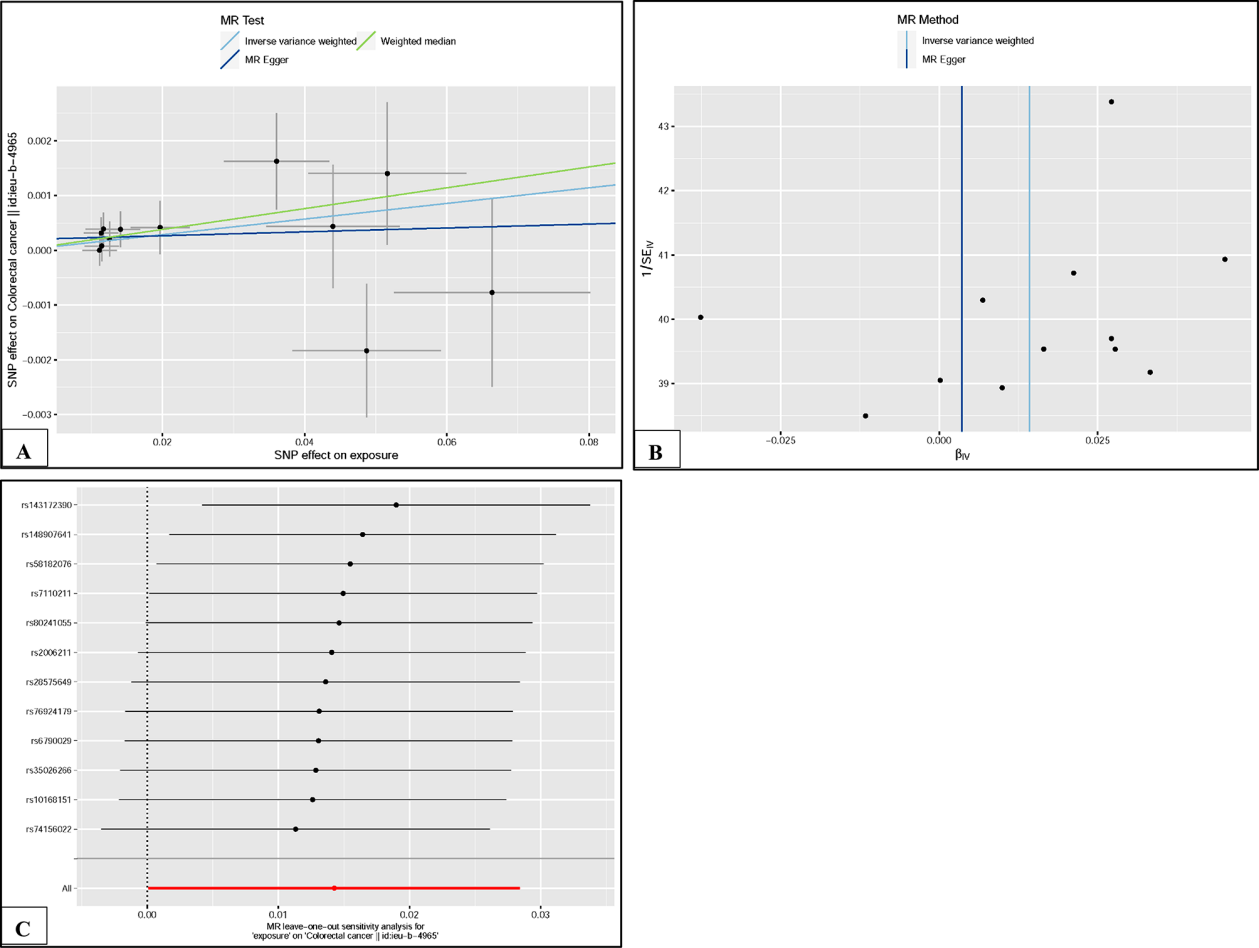
(**A**). Scatter plot of SNPs associated with vegetable and their risk of colorectal cancer. (**B**). Funnel plot of SNPs associated with vegetable and their risk of colorectal cancer. (**C**). Leave one-out of SNPs associated with vegetable and their risk of colorectal cancer

The p-value of Cochran Q test for MR-Egger was 6.13E-01 and for IVW was 6.39E-01. That is, there was no heterogeneity in the causal association between vegetables and CRC. Figure 3A shows the MR regression slope and individual causal estimates for each of the 12 SNPs. In addition, there was no evidence of significant interception (intercept = 1.95E-04, SE = 2.41E-04, P = 4.38E-01), indicating that no directional pleiotropy was observed. In addition, the funnel plot was symmetrical, suggesting no pleiotropy (Fig. 3B). In the leave-one-out sensitivity analysis, no single SNP strongly violated the overall effect of vegetables on CRC (Fig. 3C).

We found no potential causal association between vegetables (female) (OR = 1.003, 95% CI = 0.981–1.026, p = 0.788), vegetables (male) (OR = 1.006, 95% CI = 0.995–1.017, p = 0.320), fruit (OR = 1.007, 95% CI = 0.986–1.029, p = 0.512), red beef intake (OR = 1.002, 95% CI = 0.992–1.013, p = 0.692), tea (OR = 1.002, 95% CI = 0.994–1.009, p = 0.672), meat (OR = 1.000, 95% CI = 0.987–1.013, p = 0.968), sweets (OR = 0.998, 95% CI = 0.991–1.004, p = 0.524) (Fig. 2A) on CRC risk. And there was no heterogeneity in the causal relationship between these exposure dietary factors and CRC risk (Table 1). The results of MR regression analysis, funnel plots and leave-one-out sensitivity analysis can be found in Supplementary Figs. 1–7.

We also found no potential causal effect on CRC risk for alcohol drinker status (current) (OR = 1.012, 95% CI = 0.974–1.051, p = 0.556), alcohol drinker status (never) (OR = 1.01., 95% CI = 0.957–1.067, p = 0.715), alcohol drinker status (previous) (OR = 1.001, 95% CI = 0.935–1.072, p = 0.971), alcohol (female) (OR = 1.004, 95% CI = 0.998–1.011, p = 0.184), alcohol (male) (OR = 1.001, 95% CI = 0.993–1.008, p = 0.870), alcohol intake frequency (OR = 0.999, 95% CI = 0.997–1.001, p = 0.154), white wine (OR = 1.001, 95% CI = 0.997–1.005, p = 0.708), fortified wine (OR = 0.997, 95% CI = 0.981–1.014, p = 0.764), and red wine (OR = 1.000, 95% CI = 0.997–1.002, p = 0.893) (Fig. 2B). And there was no heterogeneity in the causal relationship between these exposure dietary factors and CRC risk (Table 1). The results of MR regression analysis, funnel plots, and leave-one-out sensitivity analysis can be found in Supplementary Figs. 8–16.

Our findings suggest no potential causal link between coffee (Fig. 2C) and milk (Fig. 2D) and CRC. And there was no heterogeneity and pleiotropy in the causal relationship between these exposure factors and CRC (Table 1). The results of MR regression analysis, funnel plots and leave-one-out sensitivity analysis can be found in Supplementary Figs. 17–25.

Due to the large directional pleiotropy (P < 0.05) for the two instrumental variables of rose wine yogurt/ice cream, we deemed it of little significance to further analyze the causal association with CRC.

### Causal effects of different dietary habits on potential CRC risk factors

Our study aimed to investigate whether the association between 9 genetically determined dietary habits and CRC is influenced by pleiotropic pathways related to CRC. To achieve this, we employed the IVW approach to analyze the association between these dietary habits and various CRC risk factors, including family history of digestive organ malignancies, history of tobacco, diabetes, and inflammatory bowel disease. Our analysis revealed no causal effect of the nine dietary habits on these potential risk factors for CRC, as presented in Table 2.

**Table 2:** Mendelian randomization estimates of the associations from dietary habits on common risk factors of colorectal cancer

Outcome	Causal effect (95% CI); p value								
	Alcohol	Coffee	Fruit	Meat	Milk	Sweets	Tea	Vegetables	Yogurt-ice
DM	1.32 (0.80-2.20); 0.278	1.05 (0.86-1.28); 0.649	2.44 (0.53-11.34); 0.255	0.95 (0.59-1.51); 0.819	1.19 (0.52-2.72); 0.673	0.79 (0.50-1.26); 0.330	1.14 (0.83-1.57); 0.406	0.91 (0.11-7.62); 0.931	0.98 (0.41-2.38); 0.972
Family history	1.00 (0.99-1.00); 0.608	1.00 (0.99-1.01); 0.897	1.02 (0.99-1.06); 0.060	1.00 (0.99-1.01); 0.483	1.00 (0.99-1.01); 0.806	1.01 (0.99-1.01); 0.196	1.00 (0.99-1.01); 0.823	0.99 (0.98-1.01); 0.902	0.99 (0.98-1.00); 0.140
IBD	1.22 (0.82-1.82); 0.331	1.06 (0.90-1.24); 0.491	0.69 (0.16-3.03); 0.626	1.33 (0.90-1.97); 0.150	0.77 (0.36-1.63); 0.487	1.12 (0.79-1.60); 0.524	1.02 (0.60-1.74); 0.929	1.13 (0.40-3.23); 0.816	1.09 (0.54-2.18); 0.820
Tobacco	0.99 (0.20-4.99); 0.993	1.67 (0.80-3.48); 0.170	0.01 (0.02-3.36); 0.110	0.50 (0.09-2.68); 0.414	0.93 (0.03-33.61); 0.969	0.98 (0.13-7.14); 0.982	2.67 (0.63-11.28); 0.182	0.17 (0.01-9.68); 0.397	0.18 (0.01-136.28); 0.286

*Abbreviation*: DM, Diabetes; IBD, Inflammatory Bowel Disease

## Discussion

We employed a multi-sample MR approach to comprehensively evaluate the potential causal effect of various dietary habits on the incidence of CRC. According to our findings, no conclusive evidence supports a causal relationship between the genetic prediction of certain food habits, such as tea and coffee, and CRC risk. We did observe a causal effect of the genetic prediction of vegetables on CRC risk. However, the results for vegetable did not survive correction for multiple comparisons.

It is widely accepted that dietary fiber have chemotherapeutic potential for treating cancer through direct action in the gastrointestinal tract, such as by reducing transport time and contact of carcinogens with the colonic mucosa, increasing carcinogen binding, and production of short-chain fatty acids [25]. However, previous cohort studies and meta-analyses have shown no significant association between vegetable consumers and reduced risk of CRC [14, 15]. Interestingly, the large cohort study of the European Prospective Investigation into Cancer and Nutrition (EPIC-Oxford) showed a higher incidence of CRC in vegetarians than in meat-eaters [26].

A long-term cohort study by Gilsing et al. showed that vegetarians did not have a significantly lower risk of colorectal cancer compared to 6–7 days/week meat consumers [14]. Interestingly, our findings also suggest a positive causal link between vegetables and CRC. Therefore, we disagree that eliminating animal protein sources from the diet is beneficial to human health [15]. It is recommended following the Mediterranean dietary pattern for CRC prevention, which involves a high intake of olive oil and plant foods (fruits, vegetables, legumes, nuts, and whole grains), moderate consumption of fish, poultry, dairy products, and alcohol, and a low intake of red meat, processed foods, and confectionery [27, 28].

The causal relationship between dietary habits, particularly alcohol consumption, and CRC has attracted growing attention. A MR analysis conducted on a Japanese population suggests a potential causal link between alcohol consumption and CRC risk in Asians [29]. Another MR analysis examining alcohol consumption and CRC risk found that genetically predicted alcohol use and consumption is a risk factor for CRC, while genetically predicted coffee consumption is protective [30].However, these studies on alcohol consumption were conducted on Asian populations. Interestingly, epidemiological evidence suggests that increased alcohol consumption is not significantly associated with CRC risk in the UK Dietary Cohort Consortium [31]. A cohort study by Song-Yi Park et al. [32] indicates that not all alcohol is associated with CRC and that the relationship between alcohol and CRC varies by race/ethnicity. Another MR study [33] involving participants from the UK Biobank and the International Genetic Alliance found no evidence to support a causal association between alcohol consumption and site-specific cancers (lung, breast, ovarian, and prostate).

Thus, we believe that the causal association between alcohol consumption and other dietary habits and the risk of CRC in European populations requires further investigation. To address this issue, we conducted a MR estimation using patterns of consumption of different genders and 10 alcohol subtypes in predominant European populations. The results revealed no causal association between alcohol consumption and CRC in predominant European populations.

In general, alcohol consumption is believed to potentially increase cancer risk through the production of its oxidative metabolite acetaldehyde, which is a known human carcinogen [34]. However, there may be other mechanisms through which alcohol consumption can reduce cancer risk, such as increased insulin sensitivity through increased lipocalin levels [35]. In particular, red wine contains flavonoids and polyphenolic compounds. Furthermore, it has been demonstrated that these compounds have chemotherapeutic potential for treating cancer and inflammation [36, 37]. Moderate alcohol consumption has been empirically shown to reduce inflammatory markers and C-peptides [38–40], and a basic study on alcohol rat models found that moderate alcohol consumption does not increase biological risk factors for CRC development and may even provide beneficial effects by reducing inflammation and decreasing DNA damage [41]. Interestingly, a large meta-analysis showed a protective association for light/moderate alcohol consumption at proximal colon, distal colon, and rectal cancer sites [42]. However, the results of the current MR study are consistent with previous MR studies in which no evidence supported an association between alcohol consumption and overall or site-specific cancer risk [33]. Nevertheless, further larger MR studies are required to confirm the genetically predicted association between high-dose, frequent alcohol consumption and CRC.

While high consumption of fruits has been suggested to reduce the risk of colon cancer, our study does not support this claim. This conclusion aligns with a 10-year follow-up cohort study conducted in the European population, which also found that fruit consumption alone did not provide protection against CRC [43]. Similarly, a cohort study of Asian populations found that fruit intake was not associated with CRC morbidity and mortality in either sex [44, 45].

A recent MR analysis study demonstrated that processed meat intake increases the risk of CRC, whereas no causal association was found between red and white meat intake and CRC [46]. In contrast, our study did not establish any causal association between meat consumption and CRC, including red meat. The ratio of red meat to white meat in the meat instrumental variable in this study could not be determined. Therefore, this conclusion needs to be further confirmed in the future.

Tea is one of the most commonly consumed beverages worldwide, and drinking tea has been hypothesized to reduce the risk of CRC. Antioxidants, such as polyphenols, in tea protect colon epithelial cells from oxidative DNA damage caused by free radicals [47, 48]. However, we are skeptical about the conclusion. most cohort studies and Meta-analyses do not support the conclusion that tea consumption reduces CRC risk [49–54]. Second, our MR analysis similarly found no evidence for a negative causal association between tea consumption (both black and green tea) and CRC. Our explanation for this finding is that, first, tea has been reported to have mutagenic and genotoxic compounds, such as tannins and caffeine [55, 56], which may increase the risk of colon cancer. Second, the brewing method and type of tea may also affect the amount of tea polyphenols [57], which cannot be fully captured using tea addition alone.

Our analysis also indicates no causal link between coffee and CRC. Complex compounds in coffee with opposing effects may account for the observed results. Coffee consumption may increase colonic peristalsis, reducing the exposure of colonic epithelial cells to potential carcinogens [47]. It may also reduce the synthesis and secretion of bile acids, which are potential colon carcinogens [58]. In contrast, caffeine in high concentrations has genotoxic and mutagenic properties, which may increase the risk of colon cancer [47]. In addition, caffeine has been shown to decrease insulin sensitivity, which may increase the risk of CRC [59, 60].

Given the marginal association between vegetable consumption and CRC, gender-specific subgroup analyses were conducted. However, the gender subgroup analysis did not support this conclusion. Based on this, we believe that future validation will require additional quantitative data. Existing studies provide evidence of a dose-response relationship between different dietary habits and CRC. For example, the meta-analysis by Wu et al. [61]showed a non-linear dose-response relationship between only citrus intake and CRC risk, with the risk being minimized when intake reached 120 g/d (OR = 0.85) and no significant dose-response relationship was observed with continued increases in intake. Chen et al. [62]showed that the linear curve of red and processed meat and colorectal cancer approached its plateau at high intakes of up to about 100 g/day. Ken Horisaki et al. [63] found that the higher the coffee consumption, the higher the value of the relative risk of CRC, although there was no statistically significant.

Based on the current relevant literature, the relationship between different dietary habits and CRC is likelier to exhibit a non-linear relationship, which may only exist at a certain dose interval. The lack of consideration of the dose-response relationship in the MR analysis may be one of the reasons of our negative results. In the meantime, this conclusion needs to be further verified with MR analysis, in the future, with the existence of data on relevant dose SNPs.

The use of MR in our study allowed us to minimize the effects of confounding bias and reverse causation. The random assignment of SNPs at conception adds strength to our findings, making them more compelling than those of observational studies. Our results emphasize the importance of establishing causal relationships between dietary habits and CRC in order to inform public health policies for early prevention and timely intervention.

CRC arises through three major pathways, including the adenoma-carcinoma sequence, the serrated pathway, and the inflammatory pathway. It is an etiologically heterogeneous disease based on the anatomical location of the tumor or the overall molecular subtype alteration [64]. Genetic factors have an etiologic role in predisposing individuals to CRC. However, the majority of CRC is disseminated and is primarily attributable to a range of modifiable environmental risk factors (e.g., obesity, physical inactivity, and smoking). This confounding may have contributed to the negative causal association between dietary factors and CRC based on the patients with CRC included in our study.

However, our study has several limitations. First, the genetic information in this study’s data was insufficient to determine whether CRC is a germline or somatic mutation. The confounding effect of the two mutations may compromise the results. Second, our study data lacked specific data on the high and low doses and frequency of dietary habits, which may have affected the results. Thirdly, the source population of our data is predominantly European, and further research is needed to determine the generalizability of our findings to other populations. Fourthly, MR analysis has its own limitations, such as statistical power. In MR studies, statistical power is determined by the frequency of genetic variables used, the magnitude of the effect of the variables on risk factors, and the study sample size.

## Conclusions

Our findings suggest that there is no causal association between genetically predicted alcohol, meat, milk, sweets, tea and fruit consumption and CRC. However, we did observe a positive causal association between vegetable and CRC. The identification of causal relationships between dietary habits and CRC is crucial for designing effective preventive strategies. Our study highlights the need for a more nuanced approach to dietary recommendations for CRC prevention, with greater emphasis on adherence to the Mediterranean dietary pattern.

### Electronic supplementary material

Below is the link to the electronic supplementary material.

**Supplementary Material 1 (STables):** Harmonized SNP data related with dietary habits and colorectal cancer

**Supplementary Material 2 (SFigures):** The results of MR regression analysis, funnel plots, and leave-one-out sensitivity analysis for other dietary habits

## Data Availability

All data used in the current study are publicly available GWAS summary data (https://gwas.mrcieu.ac.uk/) and (https://www.nealelab.is/uk-biobank).
