# Supplementary material for: Nine dietary habits and risk of colorectal cancer: a Mendelian randomization study
Source: BMC Med Genomics. 2024 Jan 17;17:21. doi: 10.1186/s12920-023-01782-7 (PMC10795375; doi:10.1186/s12920-023-01782-7)
Supplement: Supplementary file 1 — Supplementary Material 1 (STables): Harmonized SNP data related with dietary habits and colorectal cancer [file 12920_2023_1782_MOESM1_ESM.docx]

STable 1. Harmonized SNP data related with dietary habits and colorectal cancer.

| **Outcome** | **Exposure** | **SNP** | **Exposure** | **SNP** | **Exposure** | **SNP** | **Exposure** | **SNP** | **Exposure** | **SNP** | **Exposure** | **SNP** | **Exposure** | **SNP** | **Exposure** | **SNP** | **Exposure** | **SNP** |
| --- | --- | --- | --- | --- | --- | --- | --- | --- | --- | --- | --- | --- | --- | --- | --- | --- | --- | --- |
| **Colorectal cancer** | Vegetables | rs10168151 | Vegetables (Female) | rs12150960 | Vegetables (Male) | rs11203152 | Fruit | rs10494621 | Beef intake | rs143363049 | Tea | rs11022751 | Tea | rs4817505 | Meat | rs113989970 | Sweets | rs111324333 |
|  |  | rs143172390 |  | rs147600757 |  | rs115208148 |  | rs10777934 |  | rs184723666 |  | rs11204711 |  | rs57292194 |  | rs150680068 |  | rs111664160 |
|  |  | rs148907641 |  | rs192382215 |  | rs11987083 |  | rs13209071 |  | rs186161748 |  | rs11487328 |  | rs6462899 |  | rs2344661 |  | rs116687379 |
|  |  | rs2006211 |  | rs694836 |  | rs12494705 |  | rs182241966 |  | rs328328 |  | rs1156588 |  | rs6467958 |  | rs34514934 |  | rs116978350 |
|  |  | rs28575649 |  | rs72805467 |  | rs12952673 |  | rs183484599 |  | rs72790248 |  | rs12591786 |  | rs713598 |  | rs374100127 |  | rs12613100 |
|  |  | rs35026266 |  | rs77087673 |  | rs2820716 |  | rs569278702 |  |  |  | rs1481012 |  |  |  | rs3901368 |  | rs141132601 |
|  |  | rs58182076 |  | rs78511055 |  | rs72811941 |  | rs79500551 |  |  |  | rs182050989 |  |  |  | rs72735268 |  | rs142705784 |
|  |  | rs6790029 |  |  |  | rs73929364 |  | rs9800 |  |  |  | rs185115295 |  |  |  | rs7533296 |  | rs143244685 |
|  |  | rs7110211 |  |  |  | rs78011426 |  |  |  |  |  | rs2071207 |  |  |  | rs76551256 |  | rs1454265 |
|  |  | rs74156022 |  |  |  | rs916260 |  |  |  |  |  | rs2117137 |  |  |  |  |  | rs149930778 |
|  |  | rs76924179 |  |  |  |  |  |  |  |  |  | rs2273447 |  |  |  |  |  | rs192934965 |
|  |  | rs80241055 |  |  |  |  |  |  |  |  |  | rs2465018 |  |  |  |  |  | rs2834065 |
|  |  |  |  |  |  |  |  |  |  |  |  | rs2472297 |  |  |  |  |  | rs3813452 |
|  |  |  |  |  |  |  |  |  |  |  |  | rs4410790 |  |  |  |  |  | rs536475645 |
|  |  |  |  |  |  |  |  |  |  |  |  | rs4808940 |  |  |  |  |  | rs56171198 |
|  |  |  |  |  |  |  |  |  |  |  |  | rs977474 |  |  |  |  |  | rs72791190 |
|  |  |  |  |  |  |  |  |  |  |  |  | rs9624470 |  |  |  |  |  | rs74543812 |
|  |  |  |  |  |  |  |  |  |  |  |  | rs73424602 |  |  |  |  |  | rs79632740 |

STable 1 (continued). Harmonized SNP data related with dietary habits and colorectal cancer.

| **Outcome** | **Exposure** | **SNP** | **Exposure** | **SNP** | **Exposure** | **SNP** | **Exposure** | **SNP** | **Exposure** | **SNP** | **Exposure** | **SNP** |
| --- | --- | --- | --- | --- | --- | --- | --- | --- | --- | --- | --- | --- |
| **Colorectal cancer** | Decaffeinated coffee | rs112308424 | Ground coffee | rs1014174 | Ground coffee | rs143924702 | Ground coffee | rs4479040 | Instant coffee | rs1014174 | Other type of coffee | rs142028932 |
|  |  | rs116237319 |  | rs10162425 |  | rs145204273 |  | rs4525525 |  | rs10989557 |  | rs181147464 |
|  |  | rs116266375 |  | rs10217192 |  | rs1489786 |  | rs4869748 |  | rs11191266 |  | rs4899463 |
|  |  | rs11966489 |  | rs10236939 |  | rs150051650 |  | rs543714 |  | rs114831445 |  | rs73173688 |
|  |  | rs12615894 |  | rs10434249 |  | rs1637365 |  | rs56950501 |  | rs11690828 |  | rs75994344 |
|  |  | rs13107325 |  | rs10477887 |  | rs16954055 |  | rs61810288 |  | rs1192831 |  | rs889910 |
|  |  | rs145736159 |  | rs10499014 |  | rs16958414 |  | rs62168767 |  | rs1291818 |  |  |
|  |  | rs17496153 |  | rs10508669 |  | rs17031272 |  | rs62373023 |  | rs13400338 |  |  |
|  |  | rs28472599 |  | rs10842702 |  | rs181934624 |  | rs6434604 |  | rs1421085 |  |  |
|  |  | rs34504554 |  | rs10854853 |  | rs182823 |  | rs6450140 |  | rs1440755 |  |  |
|  |  | rs55969935 |  | rs10959073 |  | rs190114911 |  | rs6493266 |  | rs201145892 |  |  |
|  |  | rs57603836 |  | rs11068503 |  | rs197392 |  | rs6740512 |  | rs2012697 |  |  |
|  |  | rs61902817 |  | rs11124812 |  | rs198459 |  | rs701819 |  | rs2352749 |  |  |
|  |  | rs7189450 |  | rs112755386 |  | rs2080727 |  | rs7174499 |  | rs2393986 |  |  |
|  |  | rs7532549 |  | rs113782948 |  | rs2106727 |  | rs72645432 |  | rs2466826 |  |  |
|  |  | rs7857828 |  | rs113909781 |  | rs2143974 |  | rs72745349 |  | rs35107470 |  |  |
|  |  |  |  | rs11598047 |  | rs2399510 |  | rs7373404 |  | rs3789044 |  |  |
|  |  |  |  | rs11661878 |  | rs241206 |  | rs75014231 |  | rs3827811 |  |  |
|  |  |  |  | rs11672923 |  | rs2472297 |  | rs7572185 |  | rs4410790 |  |  |
|  |  |  |  | rs11724252 |  | rs2478842 |  | rs7596971 |  | rs56391050 |  |  |
|  |  |  |  | rs11735256 |  | rs28445226 |  | rs7603550 |  | rs61084367 |  |  |
| **Colorectal cancer** |  |  |  | rs11765366 |  | rs28661781 |  | rs76112426 |  | rs61783464 |  |  |
|  |  |  |  | rs11782665 |  | rs309137 |  | rs7613360 |  | rs6736768 |  |  |
|  |  |  |  | rs12323725 |  | rs3113236 |  | rs7897379 |  | rs6810182 |  |  |
|  |  |  |  | rs12500482 |  | rs324416 |  | rs7948839 |  | rs6892116 |  |  |
|  |  |  |  | rs12681375 |  | rs339101 |  | rs7963859 |  | rs7034200 |  |  |
|  |  |  |  | rs12735232 |  | rs34607402 |  | rs822506 |  | rs7274597 |  |  |
|  |  |  |  | rs13070166 |  | rs34860389 |  | rs834233 |  | rs72803914 |  |  |
|  |  |  |  | rs13135092 |  | rs35073649 |  | rs853966 |  | rs75734647 |  |  |
|  |  |  |  | rs13650 |  | rs35626515 |  | rs9269931 |  | rs76462340 |  |  |
|  |  |  |  | rs136803 |  | rs3747631 |  | rs9570035 |  | rs77458514 |  |  |
|  |  |  |  | rs142899040 |  | rs377337104 |  | rs9597588 |  | rs7779393 |  |  |
|  |  |  |  | rs143677168 |  | rs3816054 |  | rs975924 |  | rs834233 |  |  |
|  |  |  |  | rs1437971 |  | rs4324899 |  | rs986408 |  | rs9402795 |  |  |
|  |  |  |  |  |  |  |  | rs989134 |  |  |  |  |
|  |  |  |  |  |  |  |  | rs9938265 |  |  |  |  |
|  |  |  |  |  |  |  |  | rs993992 |  |  |  |  |

STable 1 (continued). Harmonized SNP data related with both different alcohol intake and colorectal cancer.

| **Outcome** | **Exposure** | **SNP** | **Exposure** | **SNP** | **Exposure** | **SNP** | **Exposure** | **SNP** | **Exposure** | **SNP** | **Exposure** | **SNP** |
| --- | --- | --- | --- | --- | --- | --- | --- | --- | --- | --- | --- | --- |
| **Colorectal cancer** | **Alcohol status (current)** | rs10860862 | **Alcohol status (never)** | rs11099302 | **Alcohol status (previous)** | rs114604150 | **Fortified wine** | rs10481384 | **Red wine** | rs11075236 | **White wine** | rs10766353 |
|  |  | rs11580470 |  | rs11634582 |  | rs114699376 |  | rs11489423 |  | rs113115416 |  | rs115731259 |
|  |  | rs1229984 |  | rs11681722 |  | rs115362331 |  | rs116739091 |  | rs115220335 |  | rs11646751 |
|  |  | rs13060312 |  | rs117974750 |  | rs138924627 |  | rs117013190 |  | rs11678890 |  | rs140194742 |
|  |  | rs13138393 |  | rs1229984 |  | rs144702203 |  | rs117981079 |  | rs1229984 |  | rs141408641 |
|  |  | rs138418035 |  | rs13270016 |  | rs146202013 |  | rs12205305 |  | rs2055235 |  | rs17145750 |
|  |  | rs140861020 |  | rs149751420 |  | rs1634504 |  | rs139280707 |  | rs3764045 |  | rs181317153 |
|  |  | rs146011154 |  | rs16931717 |  | rs17623606 |  | rs141213927 |  | rs402236 |  | rs181652319 |
|  |  | rs17623606 |  | rs1963781 |  | rs2126332 |  | rs142913342 |  | rs4682355 |  | rs2964333 |
|  |  | rs184186434 |  | rs2461837 |  | rs2231806 |  | rs146062714 |  | rs475583 |  | rs339866 |
|  |  | rs2158731 |  | rs2875908 |  | rs2714402 |  | rs56081915 |  | rs61826931 |  | rs559116390 |
|  |  | rs2277387 |  | rs371663005 |  | rs62126507 |  | rs56187709 |  | rs7223270 |  | rs59696398 |
|  |  | rs270207 |  | rs42850 |  | rs7506790 |  | rs74960175 |  | rs73304863 |  | rs62337162 |
|  |  | rs2807961 |  | rs56070209 |  | rs75939947 |  | rs77781660 |  | rs7475525 |  | rs6588138 |
|  |  | rs2941630 |  | rs59920895 |  | rs75958443 |  | rs78204215 |  |  |  | rs73137883 |
|  |  | rs34433511 |  | rs62243855 |  | rs76613534 |  | rs79984760 |  |  |  | rs73234113 |
|  |  | rs4786820 |  | rs6586775 |  | rs7726871 |  |  |  |  |  | rs74546476 |
|  |  | rs4982052 |  | rs6936873 |  | rs78527453 |  |  |  |  |  | rs7546286 |
|  |  | rs61887784 |  | rs72692353 |  | rs79416575 |  |  |  |  |  | rs7644783 |
|  |  | rs6837215 |  | rs72789201 |  | rs865177 |  |  |  |  |  | rs77552344 |
|  |  | rs71327134 |  | rs7584949 |  | rs9828831 |  |  |  |  |  |  |
|  |  | rs75511183 |  | rs76278098 |  |  |  |  |  |  |  |  |
|  |  | rs7564399 |  | rs9393 |  |  |  |  |  |  |  |  |
|  |  | rs76284048 |  |  |  |  |  |  |  |  |  |  |
|  |  | rs78652746 |  |  |  |  |  |  |  |  |  |  |
|  |  | rs80225984 |  |  |  |  |  |  |  |  |  |  |
|  |  | rs896809 |  |  |  |  |  |  |  |  |  |  |
|  |  | rs9909256 |  |  |  |  |  |  |  |  |  |  |

STable 1 (continued). Harmonized SNP data related with both different alcohol intake and colorectal cancer.

| **Outcome** | **Exposure** | **SNP** | **Exposure** | **SNP** | **Exposure** | **SNP** | **Exposure** | **SNP** | **Exposure** | **SNP** | **Exposure** | **SNP** | **Exposure** | **SNP** | **Exposure** | **SNP** | **Exposure** | **SNP** |
| --- | --- | --- | --- | --- | --- | --- | --- | --- | --- | --- | --- | --- | --- | --- | --- | --- | --- | --- |
| **Colorectal cancer** | **Alcohol (Female)** | rs113892220 | **Alcohol (Male)** | rs111654831 | **Alcohol intake frequency** | rs1004787 | **Alcohol intake frequency**  **（continued）** | rs12517851 | **Alcohol intake frequency**  **（continued）** | rs1602837 | **Alcohol intake frequency**  **（continued）** | rs3002288 | **Alcohol intake frequency**  **（continued）** | rs56119718 | **Alcohol intake frequency**  **（continued）** | rs7198842 | **Alcohol intake frequency**  **（continued）** | rs838146 |
|  |  | rs117439094 |  | rs11651533 |  | rs10184004 |  | rs12531287 |  | rs1622638 |  | rs33705 |  | rs561201635 |  | rs72642323 |  | rs854782 |
|  |  | rs12031543 |  | rs1229984 |  | rs1022666 |  | rs12531914 |  | rs16853589 |  | rs34121753 |  | rs56194430 |  | rs728538 |  | rs8614 |
|  |  | rs1229984 |  | rs181032983 |  | rs10278679 |  | rs12672945 |  | rs17097556 |  | rs34405762 |  | rs56228311 |  | rs72890684 |  | rs874296 |
|  |  | rs13038353 |  | rs185914244 |  | rs10454798 |  | rs12718064 |  | rs1763620 |  | rs34876113 |  | rs57281063 |  | rs72905060 |  | rs898063 |
|  |  | rs144509531 |  | rs61782448 |  | rs1078345 |  | rs12770757 |  | rs17662759 |  | rs35105141 |  | rs5750673 |  | rs7298932 |  | rs9310429 |
|  |  | rs144795637 |  | rs61869862 |  | rs10787760 |  | rs12873905 |  | rs17690703 |  | rs35277691 |  | rs5758969 |  | rs73050128 |  | rs9349379 |
|  |  | rs150659334 |  | rs75065798 |  | rs10792669 |  | rs12943365 |  | rs17698314 |  | rs35840311 |  | rs602370 |  | rs7306710 |  | rs9372625 |
|  |  | rs187350395 |  | rs79791749 |  | rs10934980 |  | rs12967878 |  | rs1788030 |  | rs35886351 |  | rs60815182 |  | rs73193340 |  | rs9395645 |
|  |  | rs62028453 |  |  |  | rs1104608 |  | rs13014929 |  | rs185799410 |  | rs4057919 |  | rs61873510 |  | rs7331794 |  | rs9542291 |
|  |  | rs73009788 |  |  |  | rs111633004 |  | rs13097366 |  | rs192724460 |  | rs4135294 |  | rs62159866 |  | rs73543300 |  | rs9646538 |
|  |  | rs76601814 |  |  |  | rs111984164 |  | rs13102973 |  | rs194868 |  | rs4239694 |  | rs62216061 |  | rs748919 |  | rs9652857 |
|  |  |  |  |  |  | rs11221547 |  | rs13135092 |  | rs1984584 |  | rs4241258 |  | rs62305780 |  | rs7495430 |  | rs9690021 |
|  |  |  |  |  |  | rs113011189 |  | rs1315005 |  | rs1995558 |  | rs4242715 |  | rs62339673 |  | rs7514579 |  | rs9814516 |
|  |  |  |  |  |  | rs113377562 |  | rs13233706 |  | rs201440 |  | rs4417025 |  | rs62466318 |  | rs76225816 |  | rs9829192 |
|  |  |  |  |  |  | rs113464391 |  | rs13241159 |  | rs202209188 |  | rs4500930 |  | rs62477431 |  | rs76749787 |  | rs9842406 |
|  |  |  |  |  |  | rs113591830 |  | rs13311425 |  | rs2055581 |  | rs4726481 |  | rs6459520 |  | rs780094 |  | rs9876389 |
|  |  |  |  |  |  | rs113695602 |  | rs13390019 |  | rs2111861 |  | rs4739105 |  | rs650558 |  | rs780569 |  | rs9880967 |
|  |  |  |  |  |  | rs1158620 |  | rs13433863 |  | rs2159935 |  | rs4739279 |  | rs6750325 |  | rs78910298 |  | rs9923768 |
|  |  |  |  |  |  | rs116212148 |  | rs1403225 |  | rs2244598 |  | rs4757589 |  | rs6773485 |  | rs7919624 |  | rs9959195 |
|  |  |  |  |  |  | rs11646721 |  | rs1421085 |  | rs2367546 |  | rs4800487 |  | rs68024891 |  | rs7991335 |  |  |
|  |  |  |  |  |  | rs11712750 |  | rs1468967 |  | rs248558 |  | rs4887069 |  | rs6947820 |  | rs79942197 |  |  |
|  |  |  |  |  |  | rs11775 |  | rs1490492 |  | rs2589029 |  | rs489062 |  | rs7000542 |  | rs80051160 |  |  |
|  |  |  |  |  |  | rs11886864 |  | rs149726000 |  | rs2590996 |  | rs4962458 |  | rs7117115 |  | rs801742 |  |  |
|  |  |  |  |  |  | rs11940694 |  | rs149971103 |  | rs2717063 |  | rs4970394 |  | rs7143137 |  | rs80292319 |  |  |
|  |  |  |  |  |  | rs12139282 |  | rs150620544 |  | rs28525613 |  | rs4982052 |  | rs7151136 |  | rs8049739 |  |  |
|  |  |  |  |  |  | rs1229984 |  | rs1515591 |  | rs28625019 |  | rs551998 |  | rs7155669 |  | rs8089704 |  |  |
|  |  |  |  |  |  | rs12436192 |  | rs1597959 |  | rs3001426 |  | rs55670412 |  | rs7192193 |  | rs8133767 |  |  |

STable 1 (continued). Harmonized SNP data related with both different alcohol intake and colorectal cancer.

| **Outcome** | **Exposure** | **SNP** | **Exposure** | **SNP** | **Exposure** | **SNP** | **Exposure** | **SNP** | **Exposure** | **SNP** |
| --- | --- | --- | --- | --- | --- | --- | --- | --- | --- | --- |
| **Colorectal cancer** | Full cream milk | rs10193212 | Skimmed milk | rs1029239 | Semi-skimmed milk | rs113871032 | Soya milk | rs10487060 | Other type of milk | rs142028932 |
|  |  | rs10224524 |  | rs1057890 |  | rs12212349 |  | rs10789340 |  | rs181147464 |
|  |  | rs10775256 |  | rs10777875 |  | rs12424485 |  | rs10981667 |  | rs4899463 |
|  |  | rs10874681 |  | rs11058139 |  | rs1426371 |  | rs111656330 |  | rs73173688 |
|  |  | rs10927060 |  | rs11209948 |  | rs2447091 |  | rs12583923 |  | rs75994344 |
|  |  | rs11055488 |  | rs114380217 |  | rs28384266 |  | rs13062337 |  | rs889910 |
|  |  | rs112472598 |  | rs11768481 |  | rs3864 |  | rs138300205 |  |  |
|  |  | rs11605348 |  | rs118173514 |  | rs427118 |  | rs143955465 |  |  |
|  |  | rs117910548 |  | rs136872 |  | rs4808973 |  | rs1923380 |  |  |
|  |  | rs118091905 |  | rs1424534 |  | rs7035530 |  | rs2066295 |  |  |
|  |  | rs12149832 |  | rs1426371 |  | rs71478073 |  | rs303752 |  |  |
|  |  | rs139296556 |  | rs16883833 |  | rs74549071 |  | rs36035061 |  |  |
|  |  | rs1992536 |  | rs184240 |  | rs76253868 |  | rs3744148 |  |  |
|  |  | rs2532681 |  | rs199974544 |  | rs79570398 |  | rs56072034 |  |  |
|  |  | rs2613500 |  | rs2685217 |  |  |  | rs6065487 |  |  |
|  |  | rs410426 |  | rs28384266 |  |  |  | rs62262054 |  |  |
|  |  | rs4953152 |  | rs3109885 |  |  |  | rs72637059 |  |  |
|  |  | rs4970836 |  | rs3817334 |  |  |  | rs726773 |  |  |
|  |  | rs55998228 |  | rs4235204 |  |  |  | rs72770858 |  |  |
|  |  | rs56985929 |  | rs427118 |  |  |  | rs72915559 |  |  |
|  |  | rs685268 |  | rs4347903 |  |  |  | rs73020958 |  |  |
|  |  | rs699534 |  | rs480110 |  |  |  | rs7430391 |  |  |
|  |  | rs7068491 |  | rs485859 |  |  |  | rs76924489 |  |  |
|  |  | rs72783775 |  | rs55872725 |  |  |  | rs78423063 |  |  |
|  |  | rs7287634 |  | rs57706252 |  |  |  | rs8085454 |  |  |
|  |  | rs7412 |  | rs7093239 |  |  |  |  |  |  |
|  |  | rs79868148 |  | rs7254892 |  |  |  |  |  |  |
|  |  |  |  | rs74849734 |  |  |  |  |  |  |
|  |  |  |  | rs75103157 |  |  |  |  |  |  |
|  |  |  |  | rs7929921 |  |  |  |  |  |  |
