# Supplementary material for: Nine dietary habits and risk of colorectal cancer: a Mendelian randomization study
Source: BMC Med Genomics. 2024 Jan 17;17:21. doi: 10.1186/s12920-023-01782-7 (PMC10795375; doi:10.1186/s12920-023-01782-7)
Supplement: Supplementary file 2 — Supplementary Material 2 (SFigures): The results of MR regression analysis, funnel plots, and leave-one-out sensitivity analysis for other dietary habits [file 12920_2023_1782_MOESM2_ESM.docx]

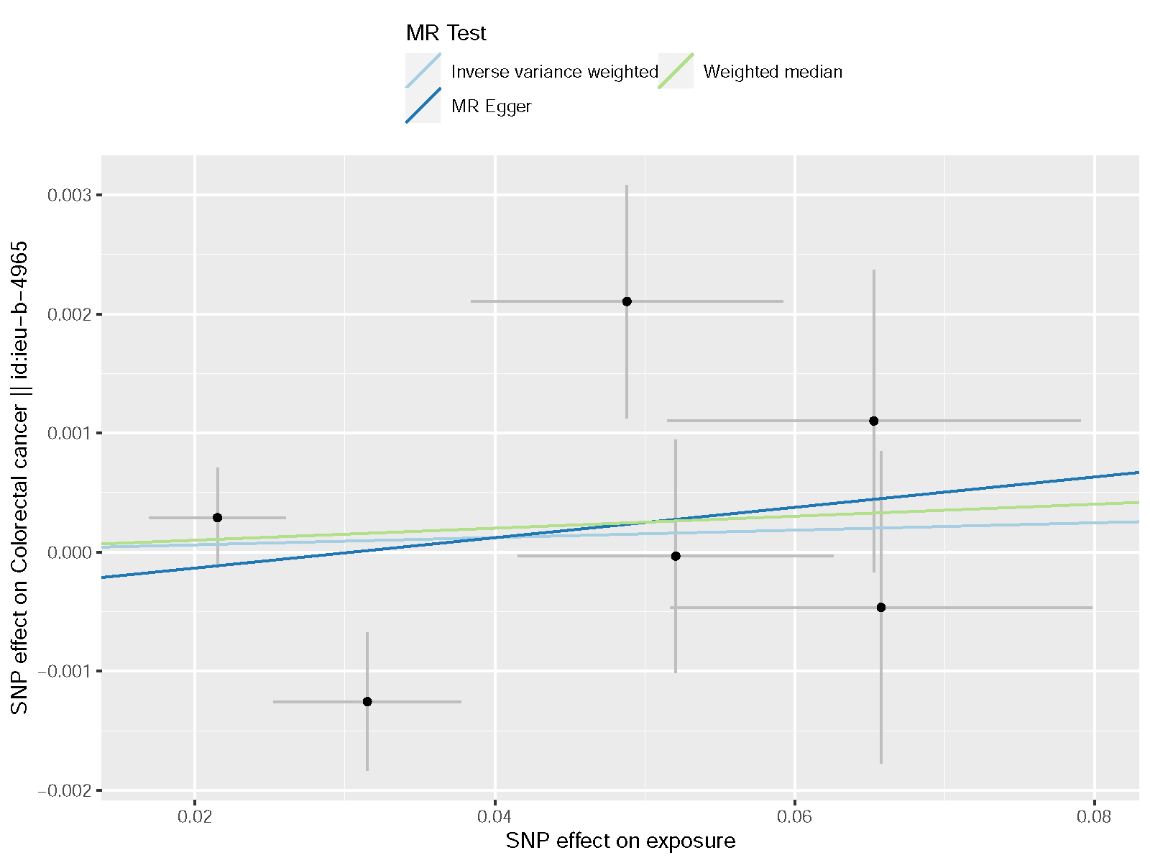

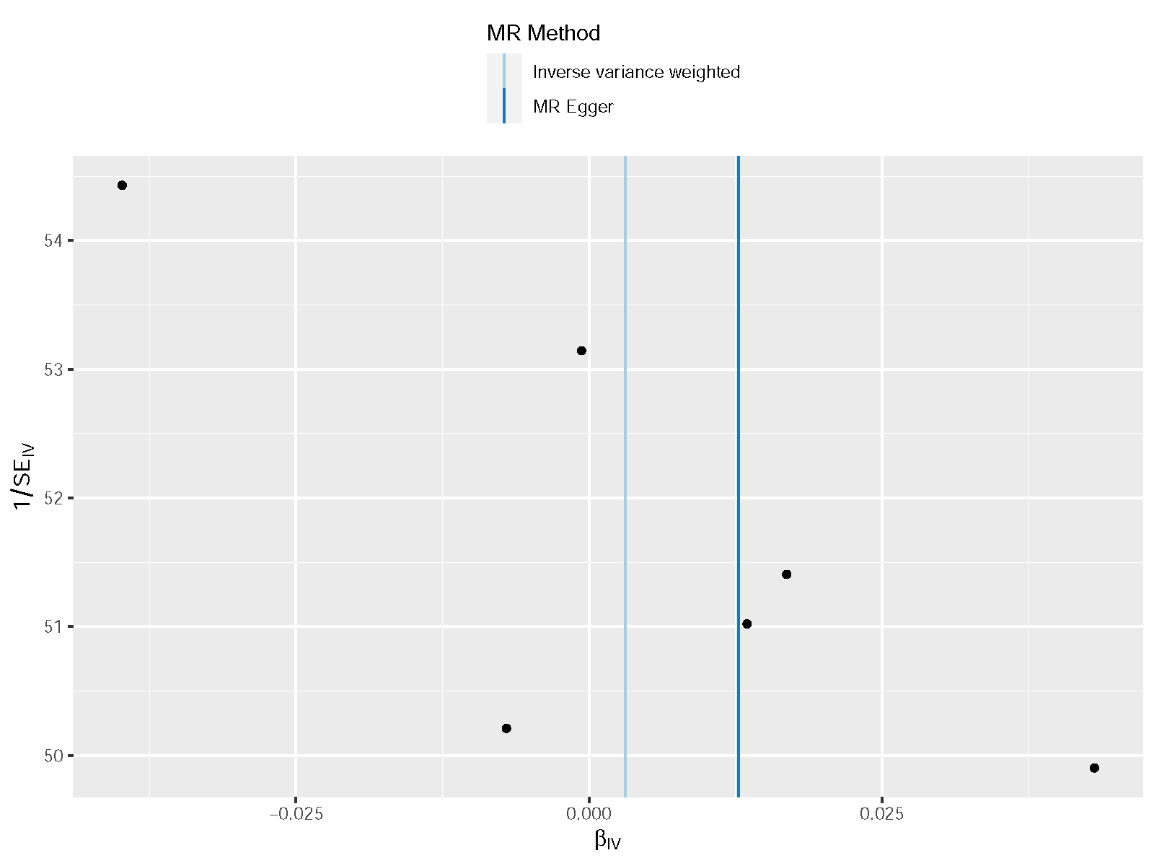

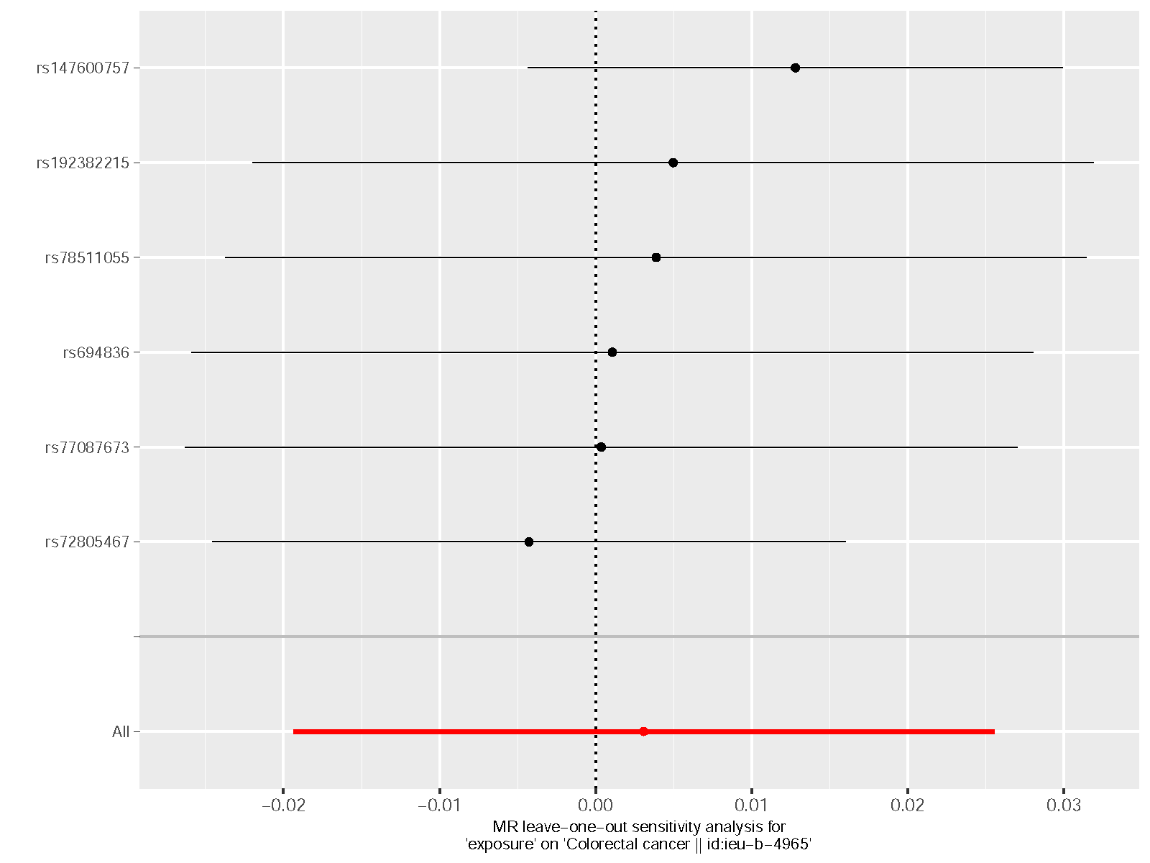


**C**

**B**

**A**

Figure S1.

A. Scatter plot of SNPs associated with Vegetable (Female) and their risk of colorectal cancer. B. Funnel plot of SNPs associated with Vegetable (Female) and their risk of colorectal cancer. C. Leave-one-out of SNPs associated with Vegetable (Female) and their risk of colorectal cancer.


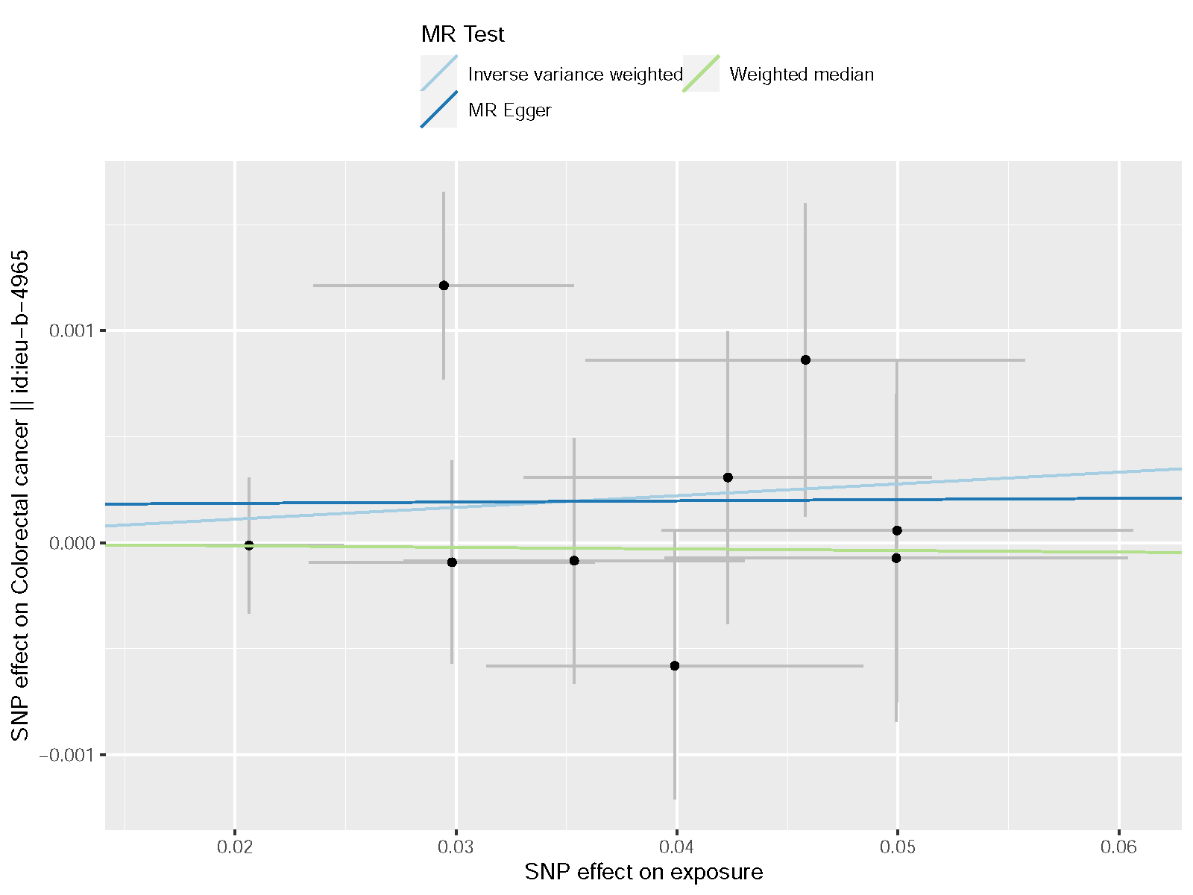

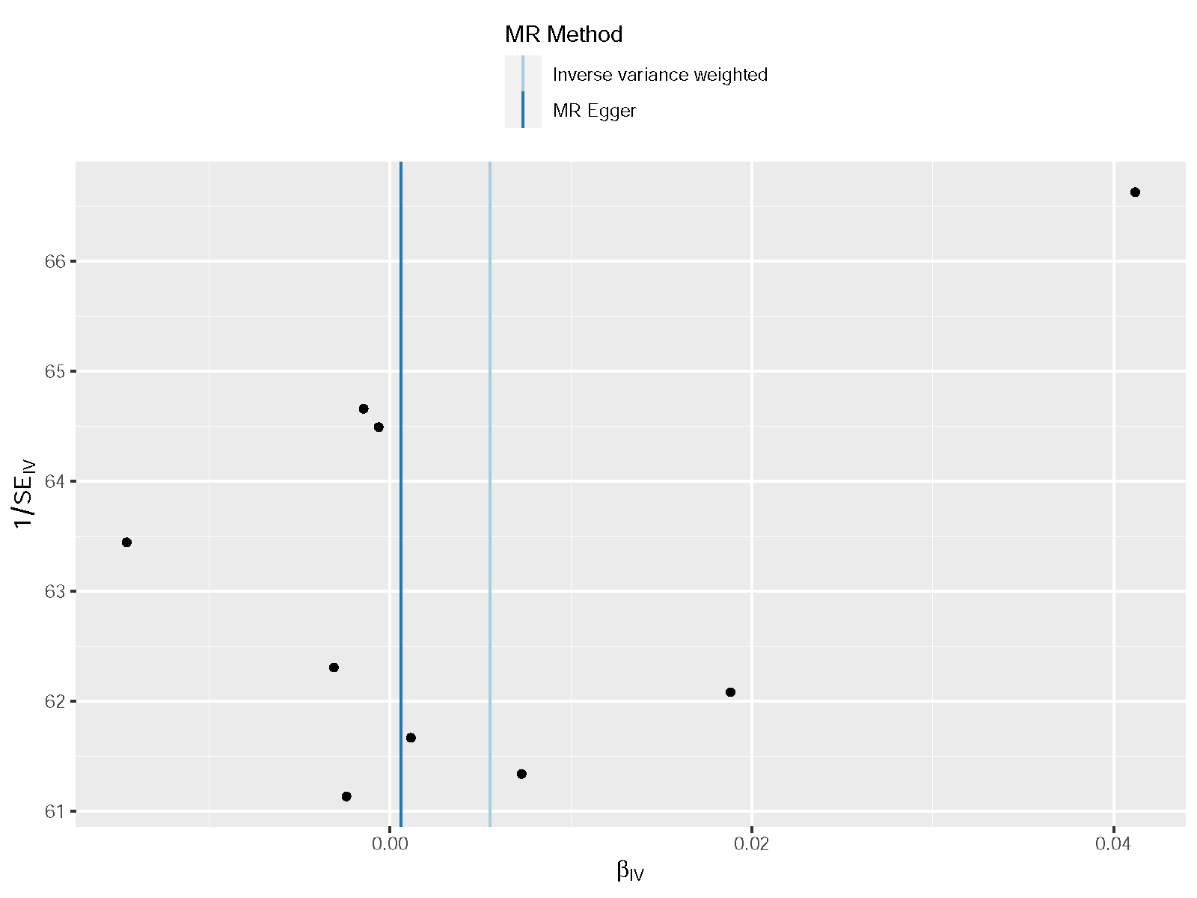

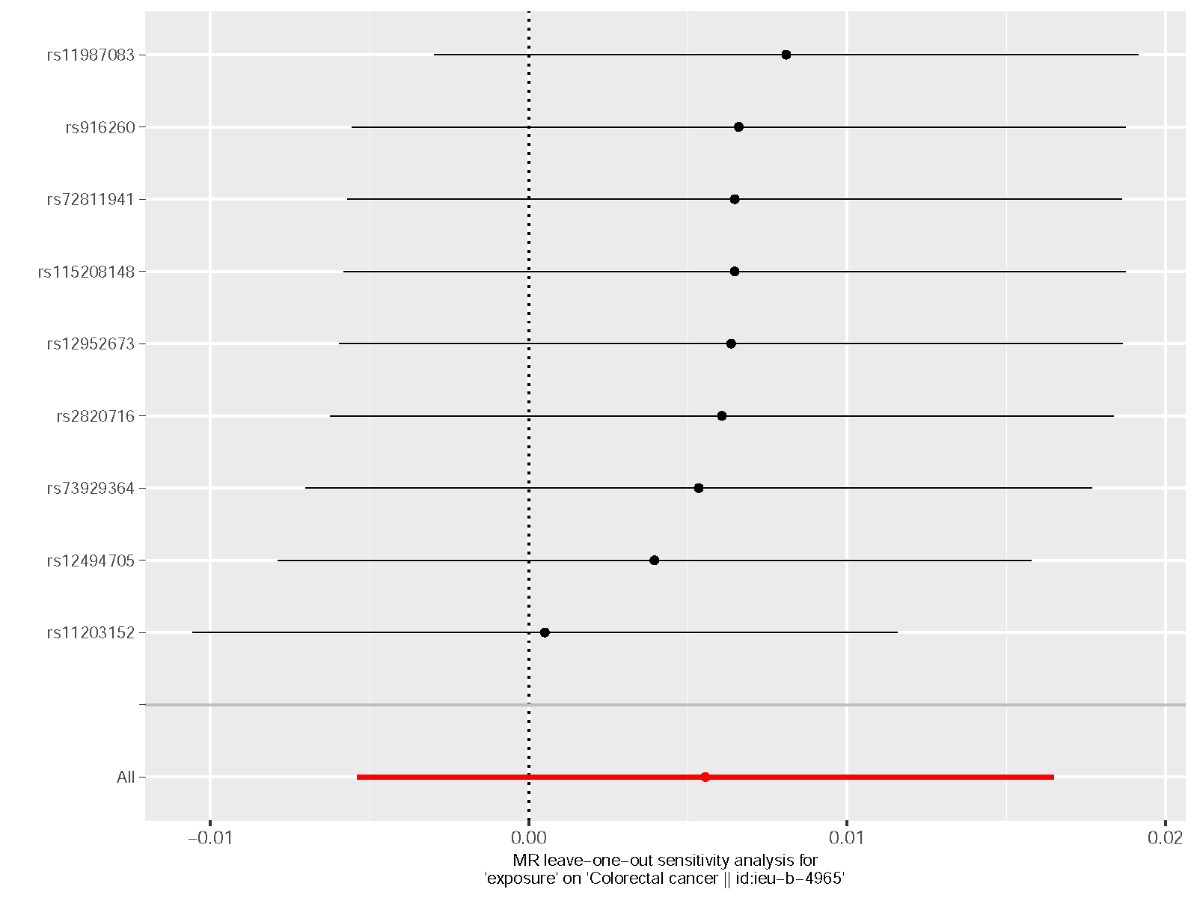


**C**

**A**

**B**

Figure S2.

A. Scatter plot of SNPs associated with Vegetable (Male) and their risk of colorectal cancer. B. Funnel plot of SNPs associated with Vegetable (Male) and their risk of colorectal cancer. C. Leave-one-out of SNPs associated with Vegetable (Male) and their risk of colorectal cancer.


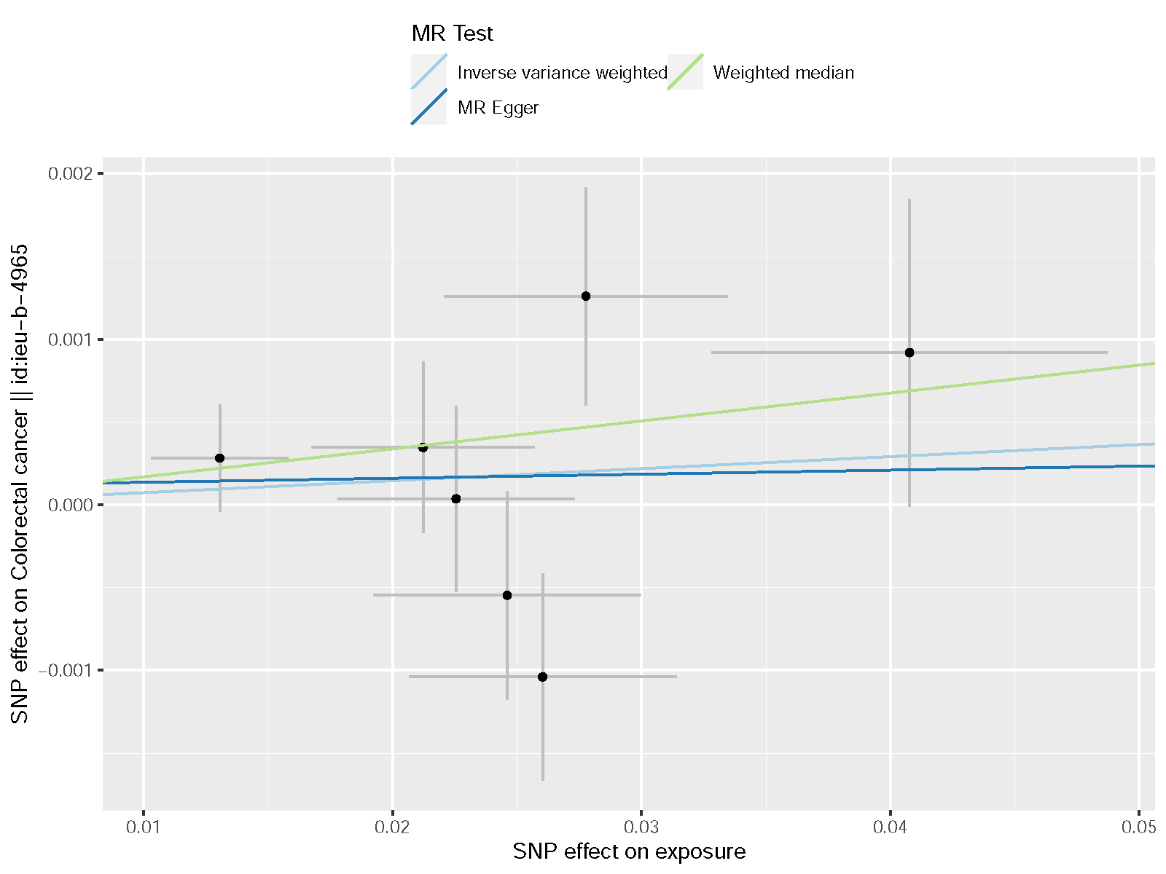

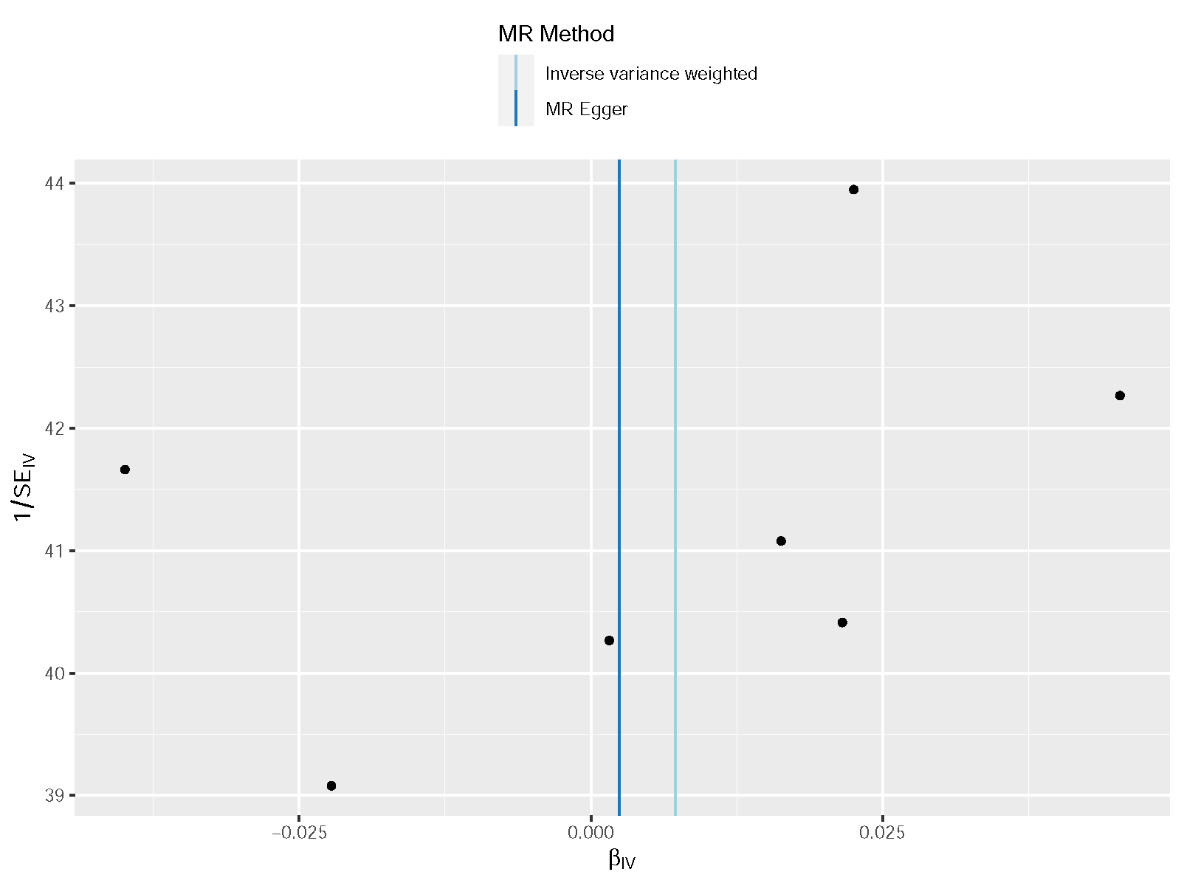

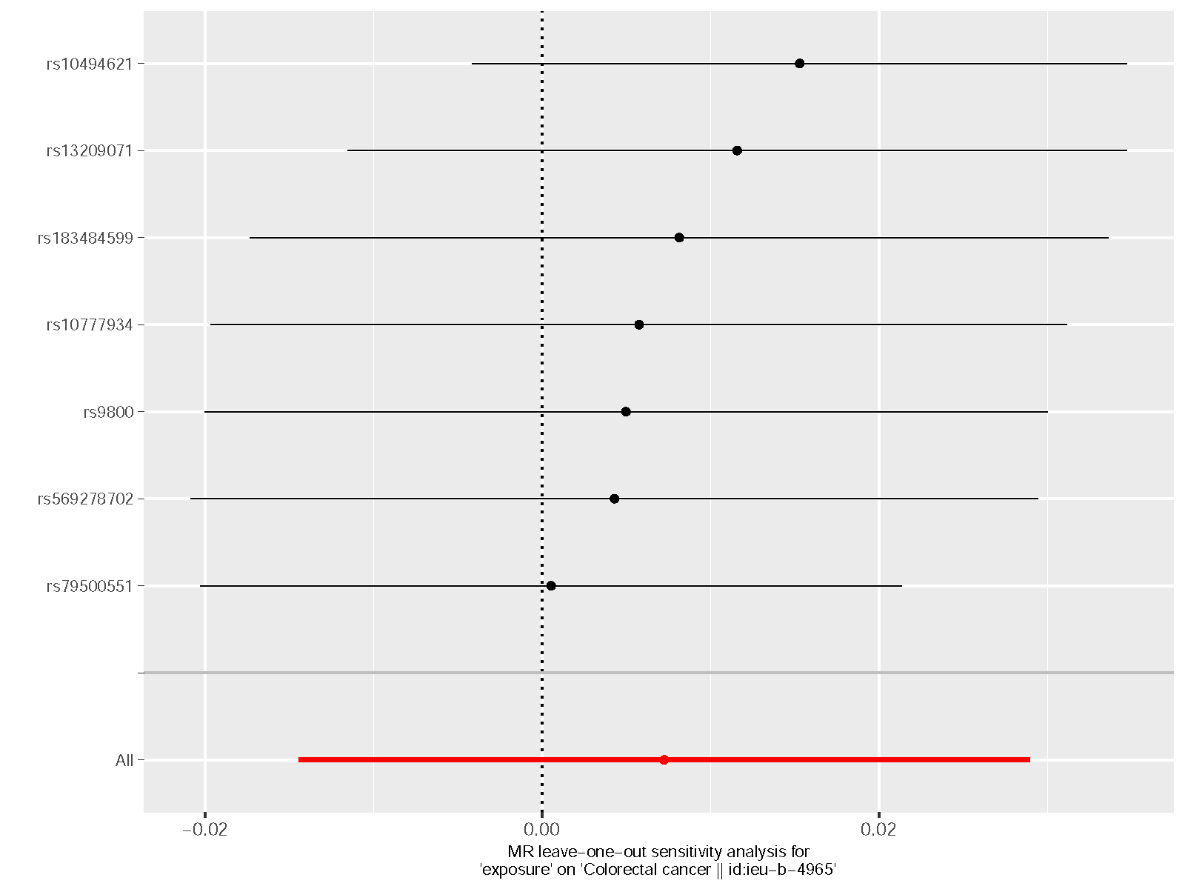


**A**

**C**

**B**

Figure S3.

A. Scatter plot of SNPs associated with Fruit and their risk of colorectal cancer. B. Funnel plot of SNPs associated with Fruit and their risk of colorectal cancer. C. Leave-one-out of SNPs associated with Fruit and their risk of colorectal cancer.


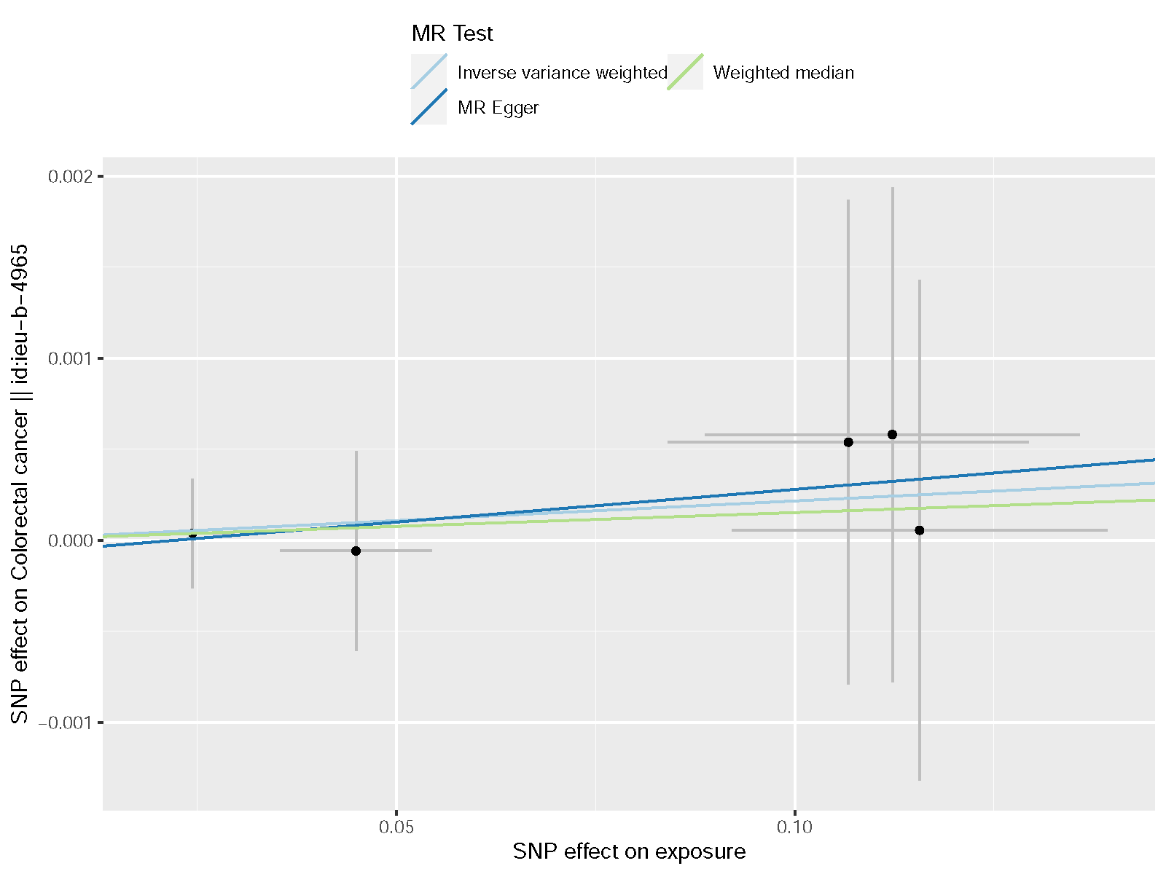

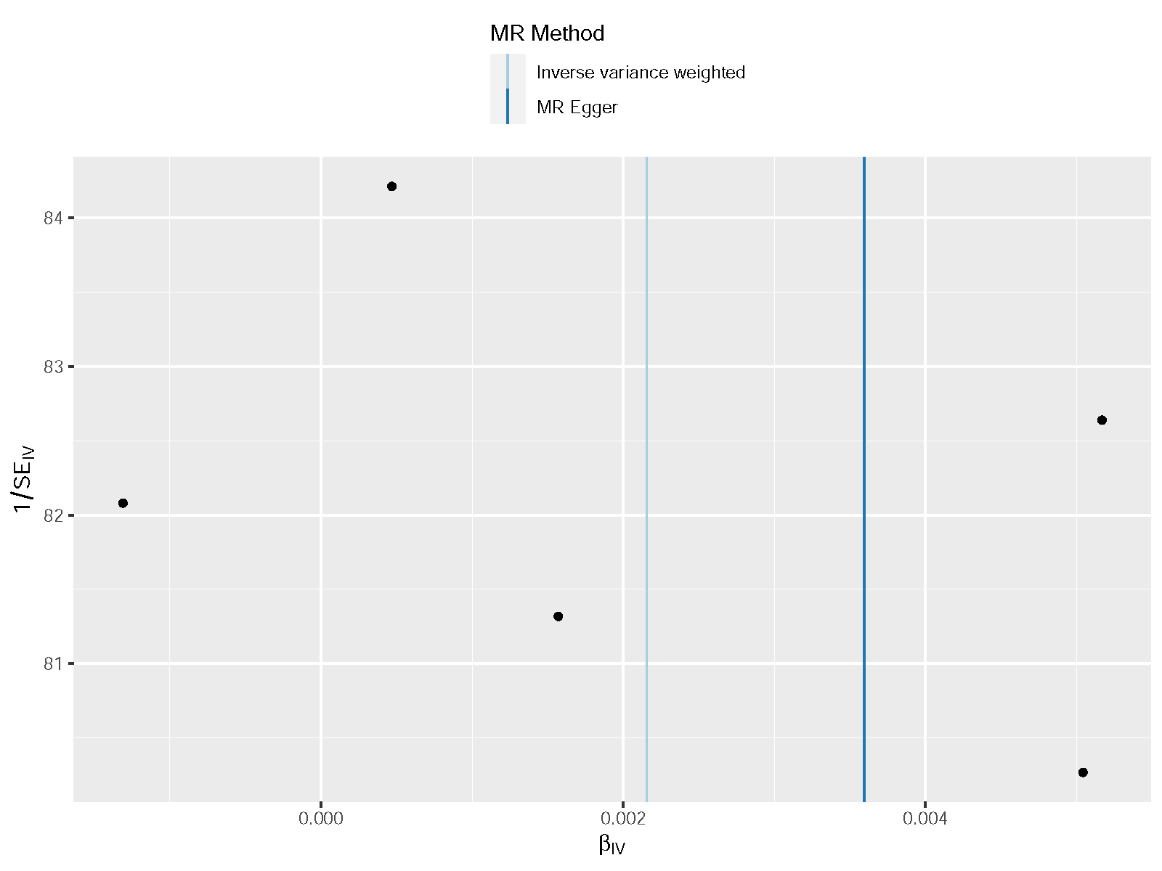

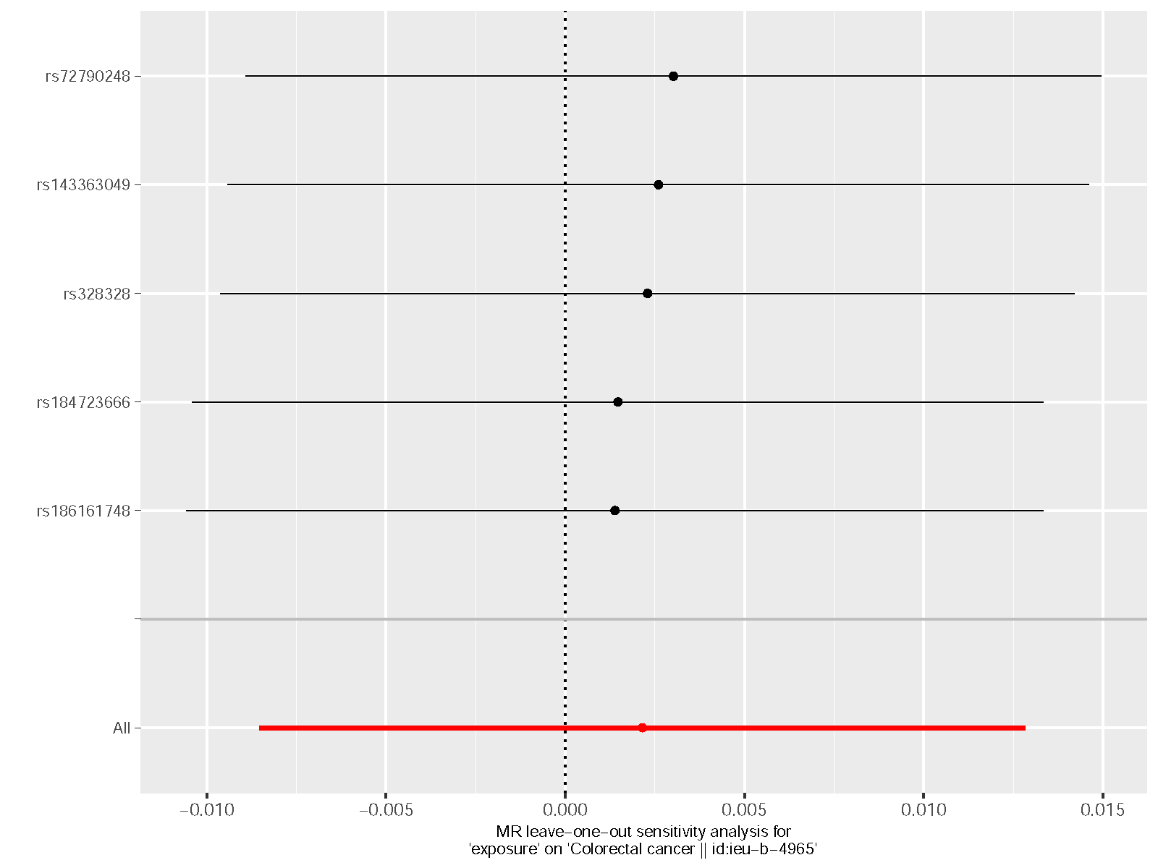


**C**

**A**

**B**

Figure S4.

A. Scatter plot of SNPs associated with Beef intake and their risk of colorectal cancer. B. Funnel plot of SNPs associated with Beef intake and their risk of colorectal cancer. C. Leave-one-out of SNPs associated with Beef intake and their risk of colorectal cancer.


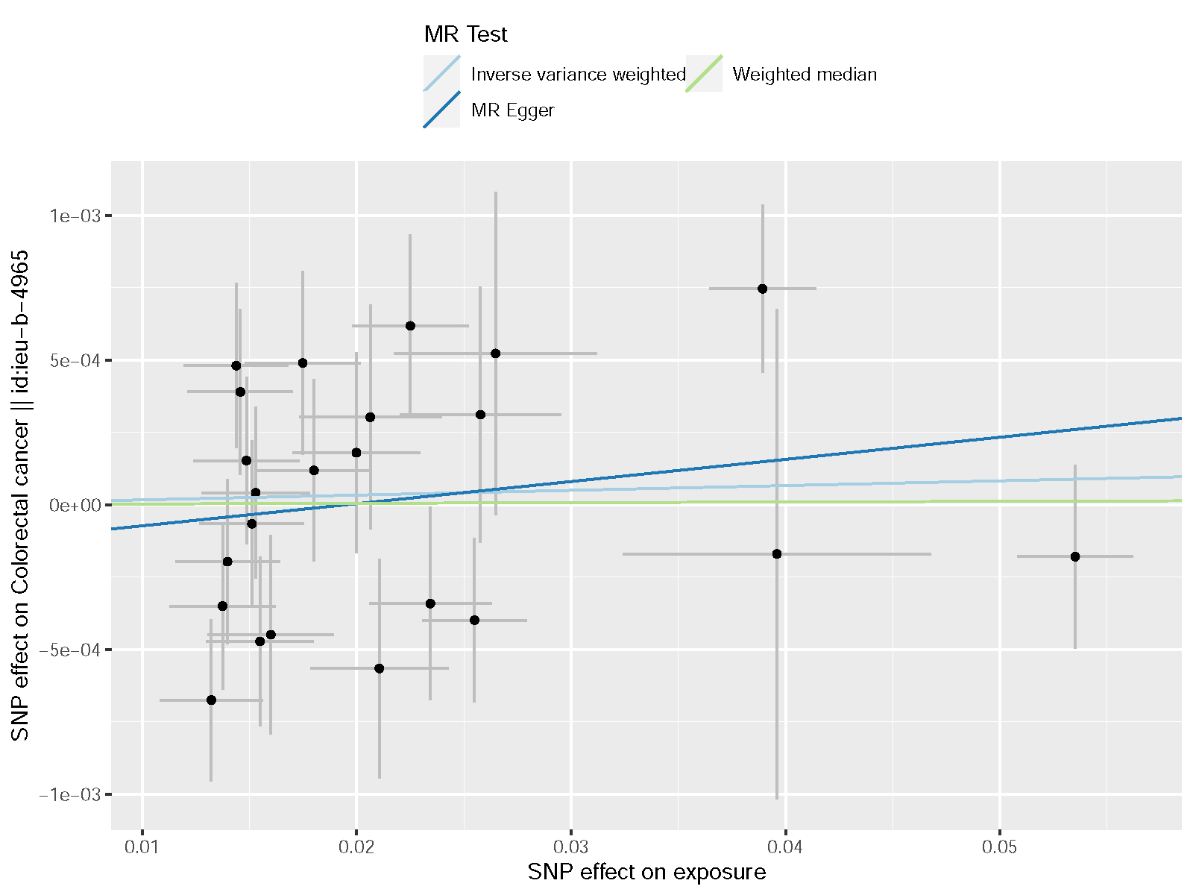

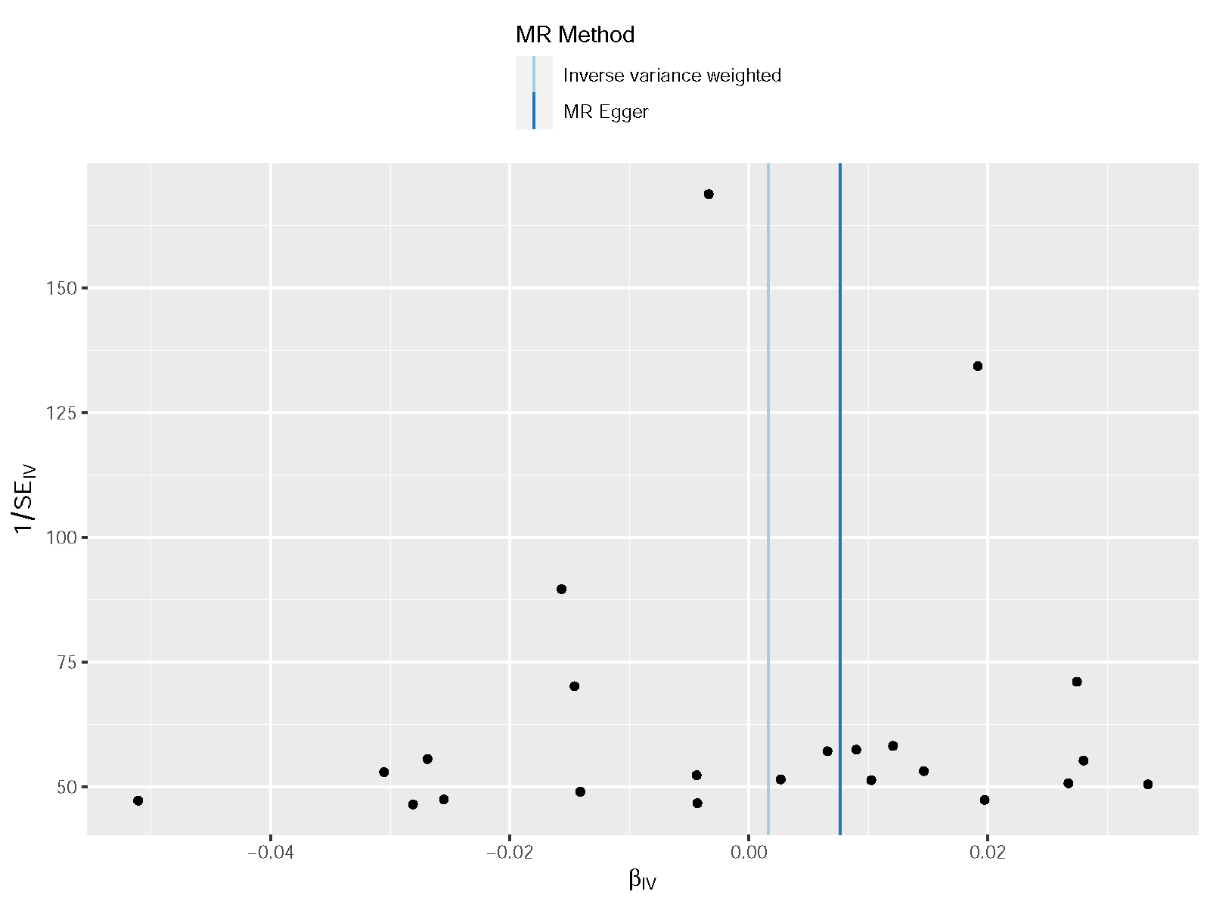

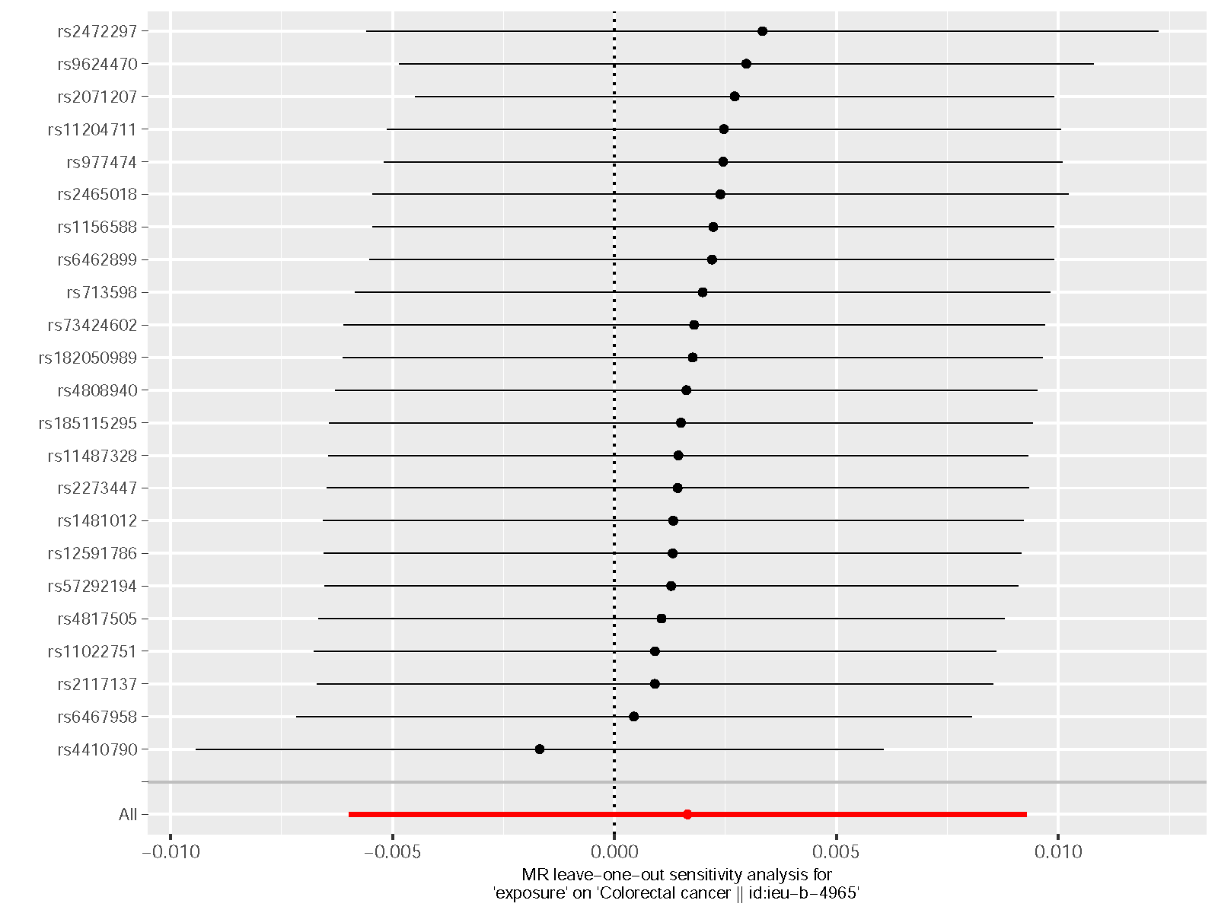


**A**

**C**

**B**

Figure S5.

A. Scatter plot of SNPs associated with Tea and their risk of colorectal cancer. B. Funnel plot of SNPs associated with Tea and their risk of colorectal cancer. C. Leave-one-out of SNPs associated with Tea and their risk of colorectal cancer.


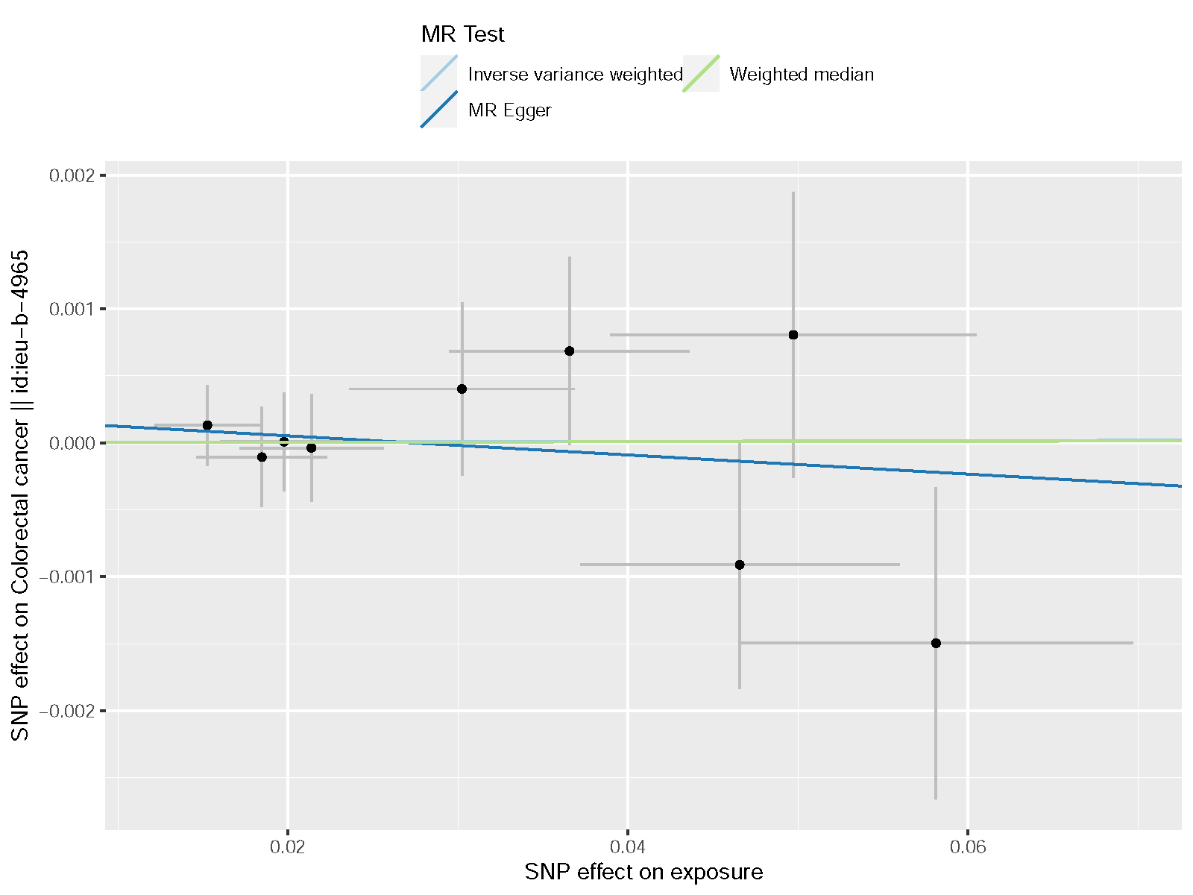

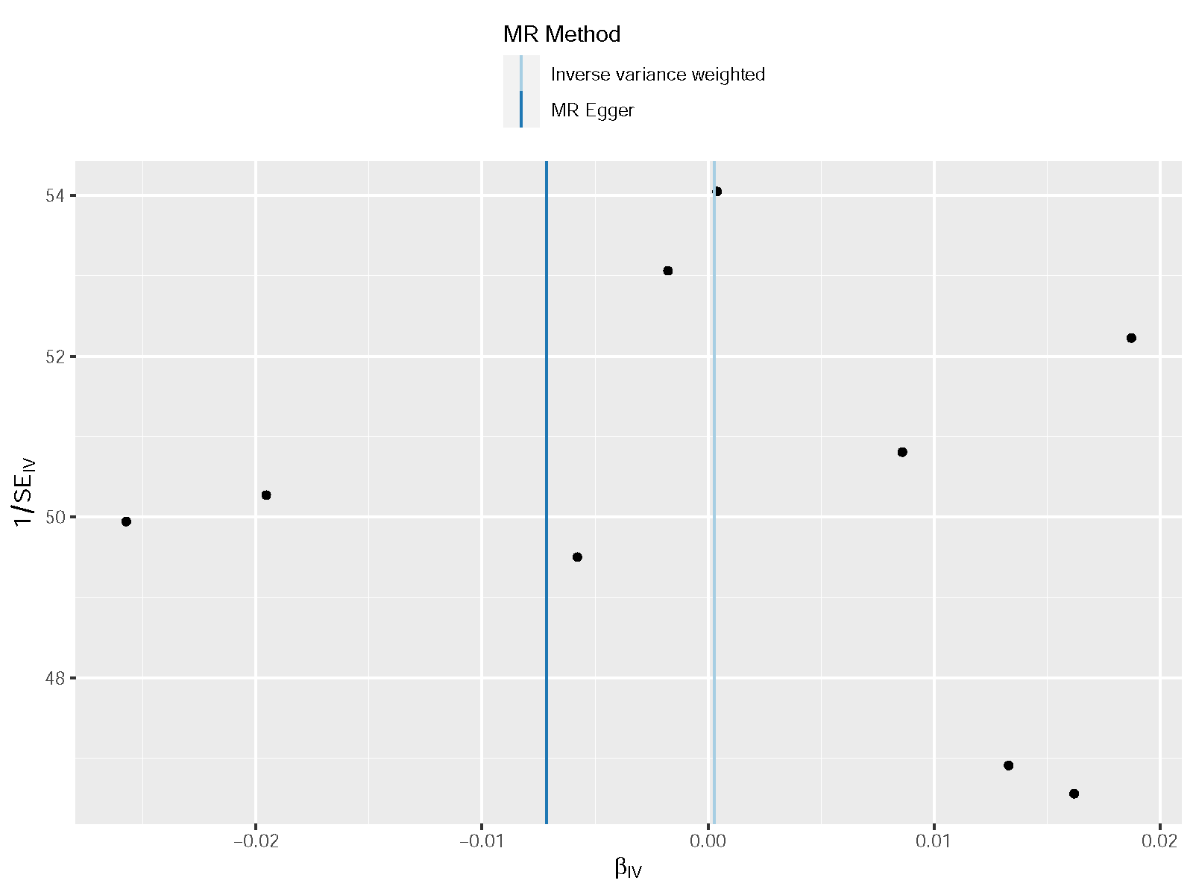

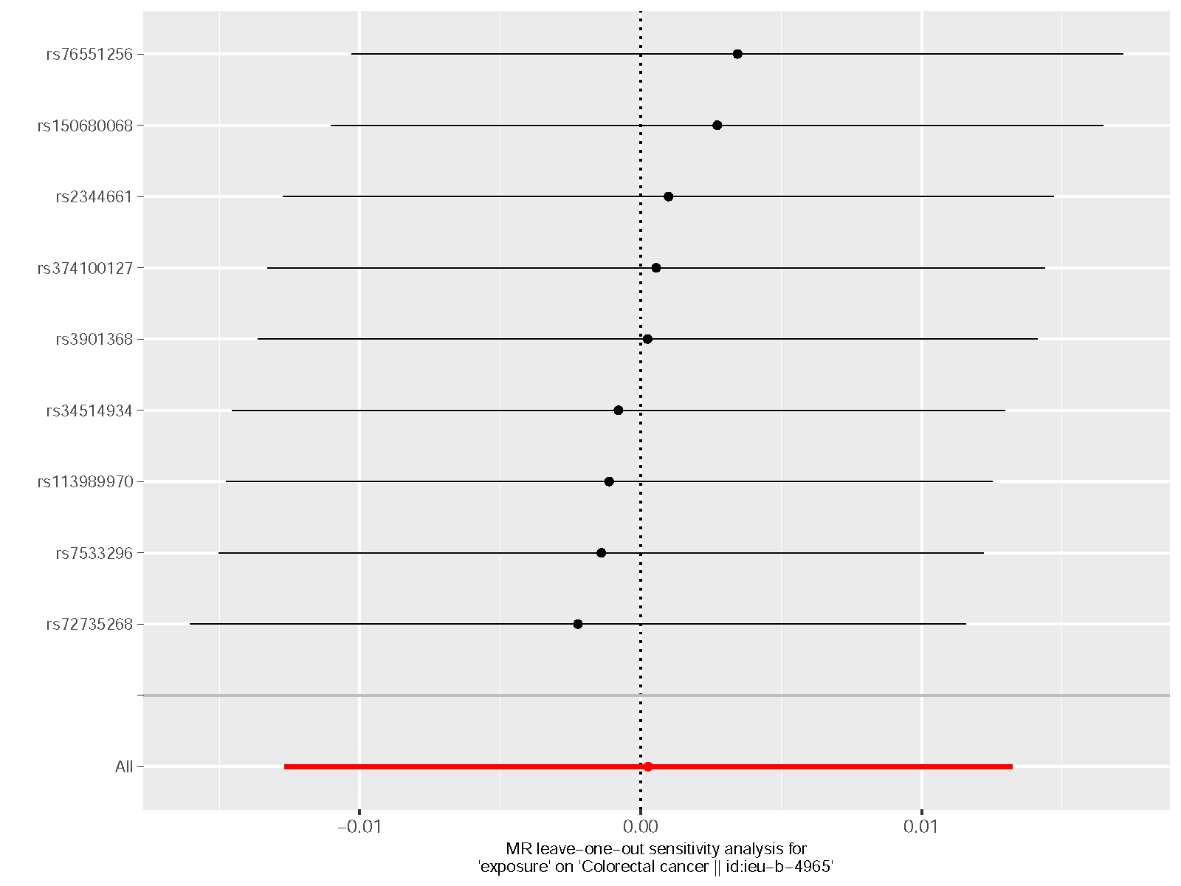


**A**

**B**

**C**

Figure S6.

A. Scatter plot of SNPs associated with Meat and their risk of colorectal cancer. B. Funnel plot of SNPs associated with Meat and their risk of colorectal cancer. C. Leave-one-out of SNPs associated with Meat and their risk of colorectal cancer.


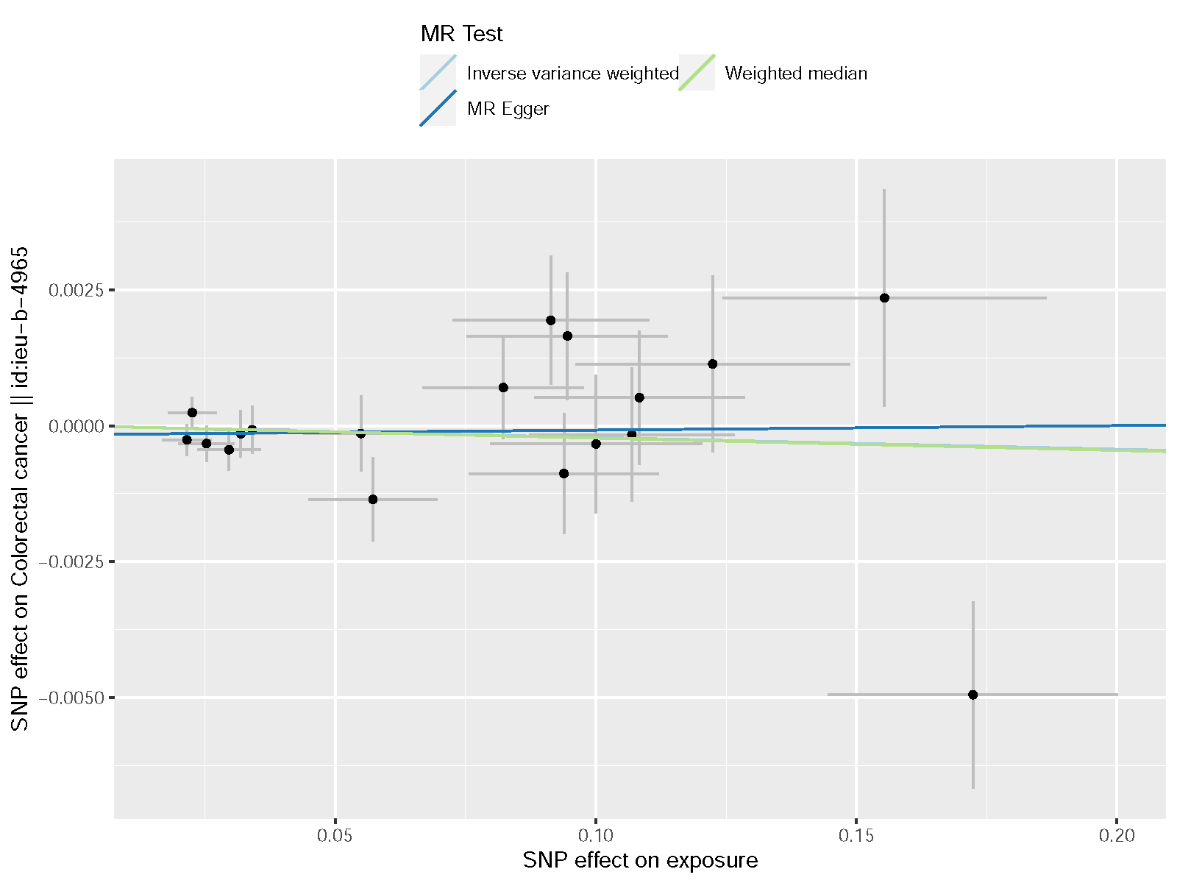

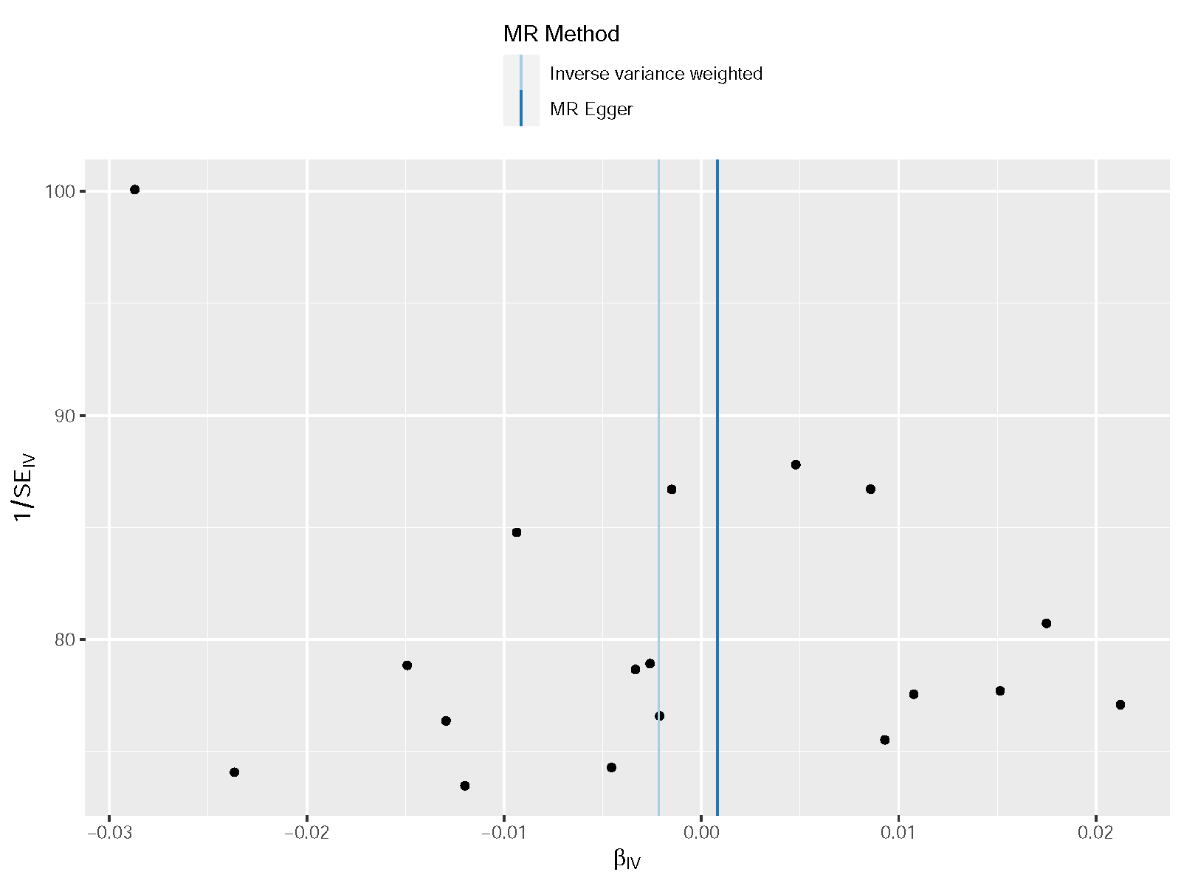

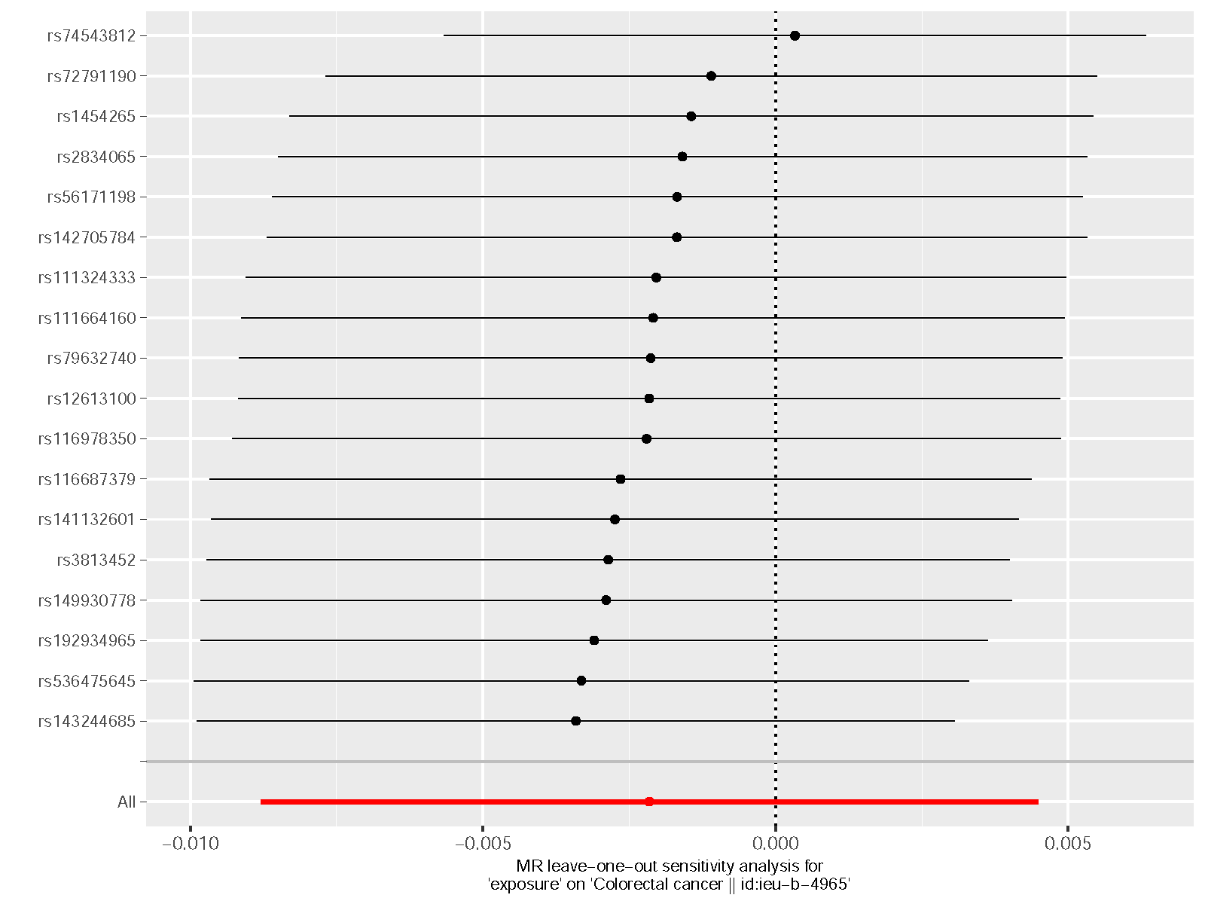


**C**

**B**

**A**

Figure S7

. A. Scatter plot of SNPs associated with Sweets and their risk of colorectal cancer. B. Funnel plot of SNPs associated with Sweets and their risk of colorectal cancer. C. Leave-one-out of SNPs associated with Sweets and their risk of colorectal cancer.


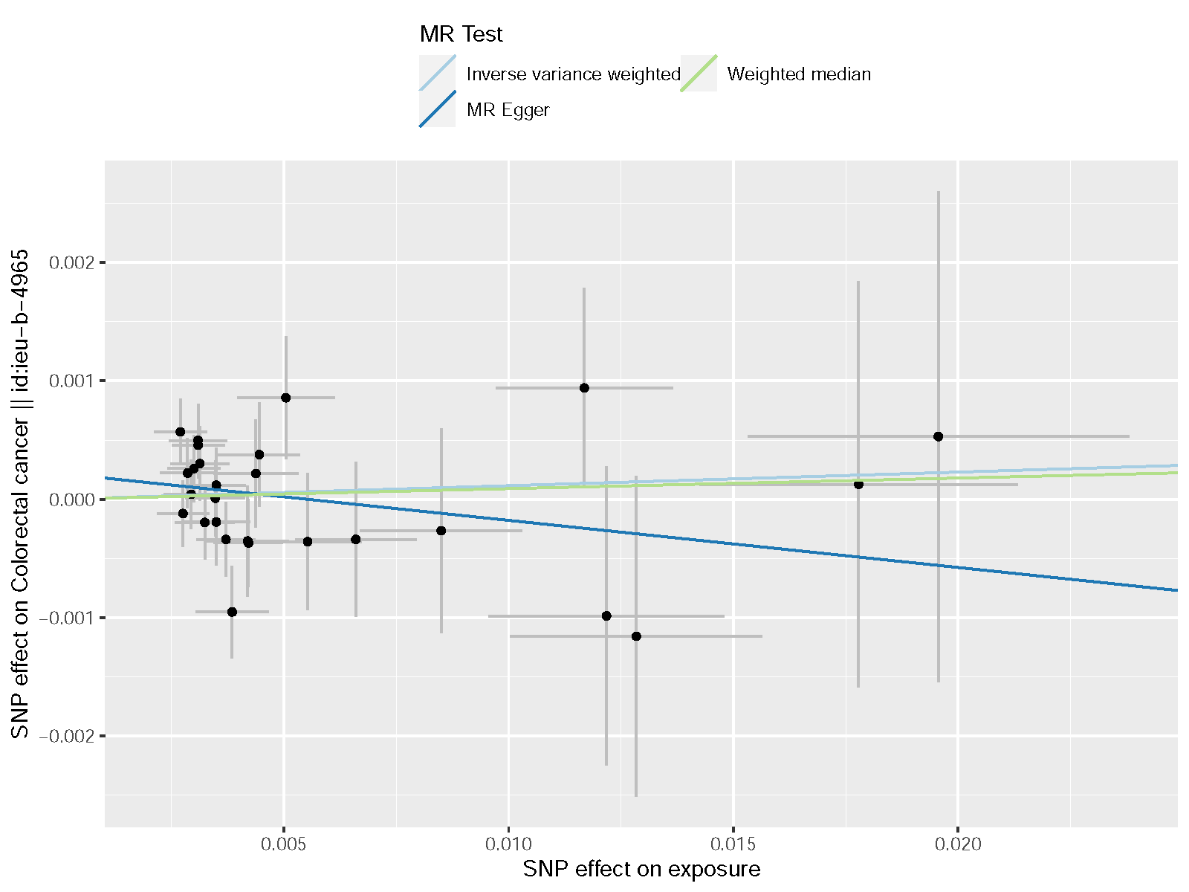

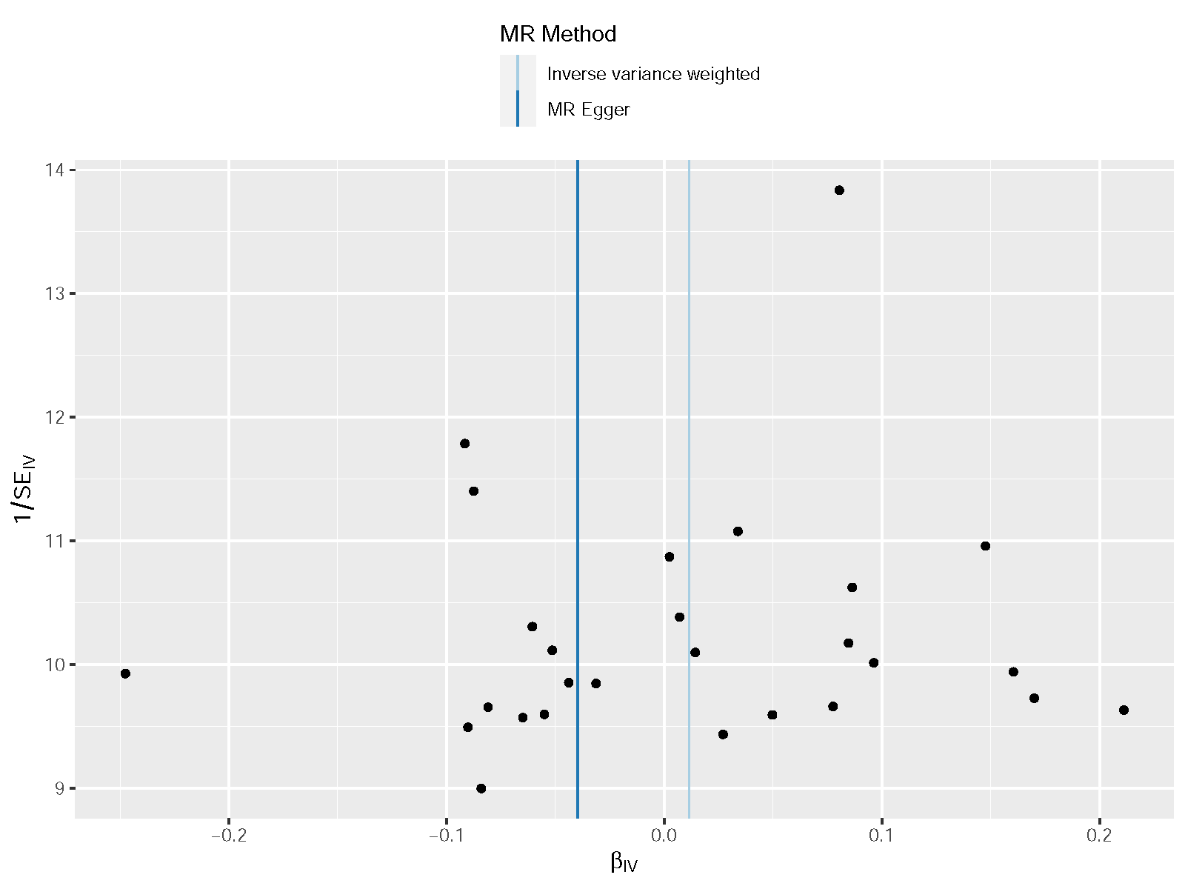

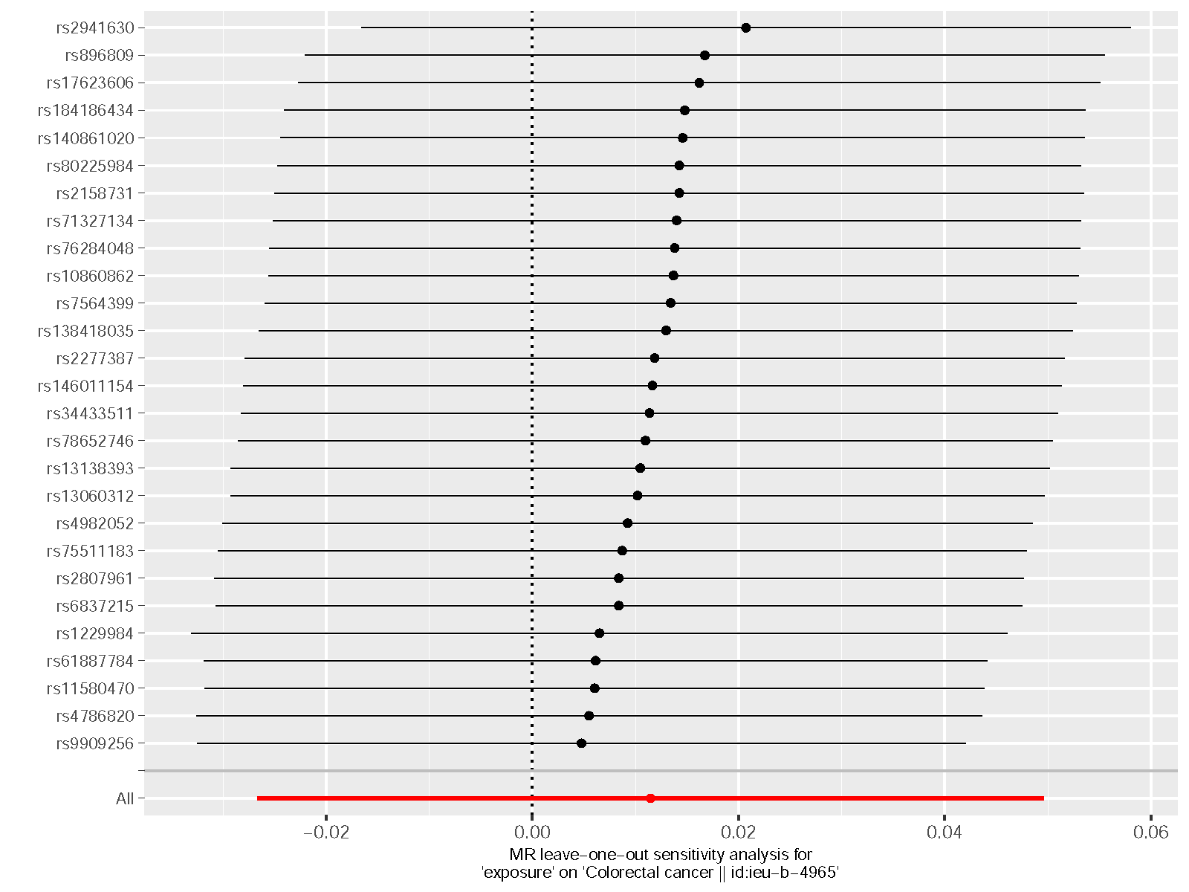


**A**

**B**

**C**

Figure S8.

A. Scatter plot of SNPs associated with Alcohol drinker status (current) and their risk of colorectal cancer. B. Funnel plot of SNPs associated with Alcohol drinker status (current) and their risk of colorectal cancer. C. Leave-one-out of SNPs associated with Alcohol drinker status (current) and their risk of colorectal cancer.


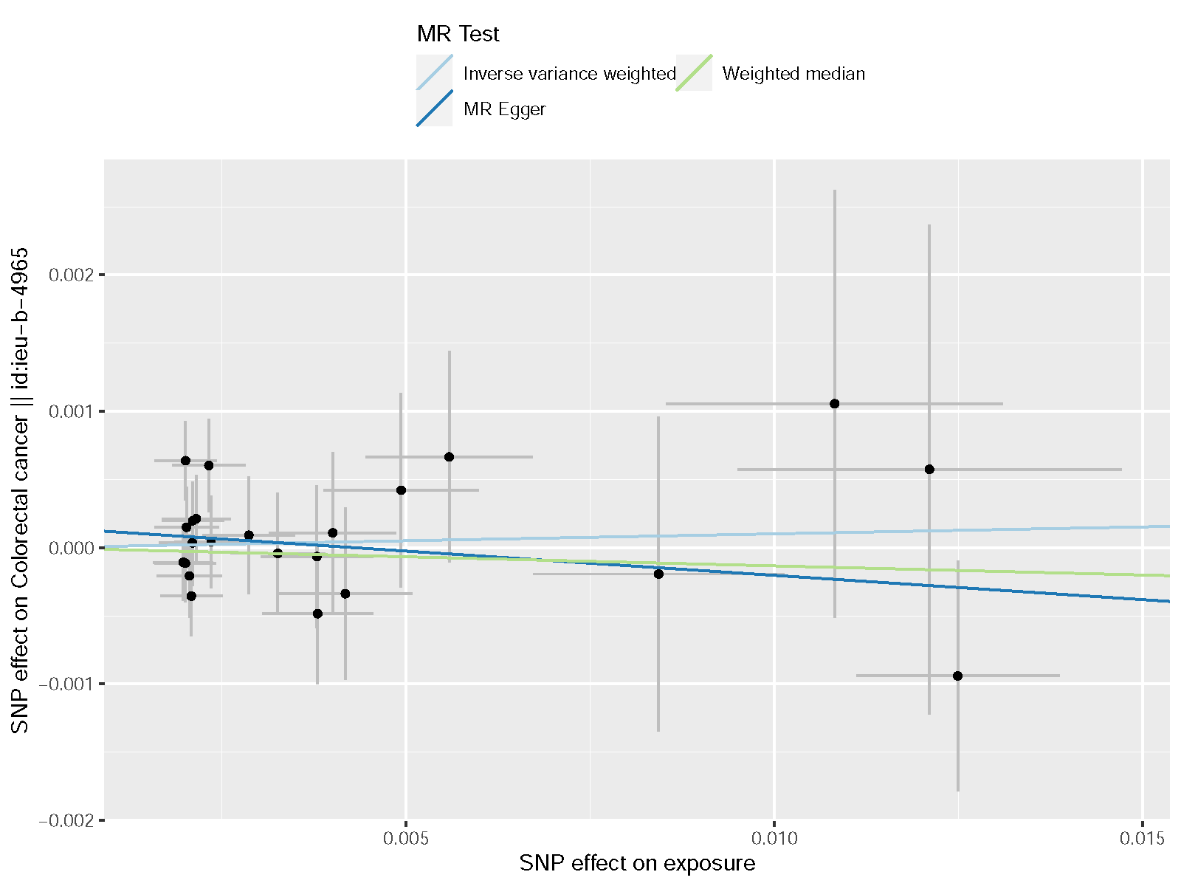

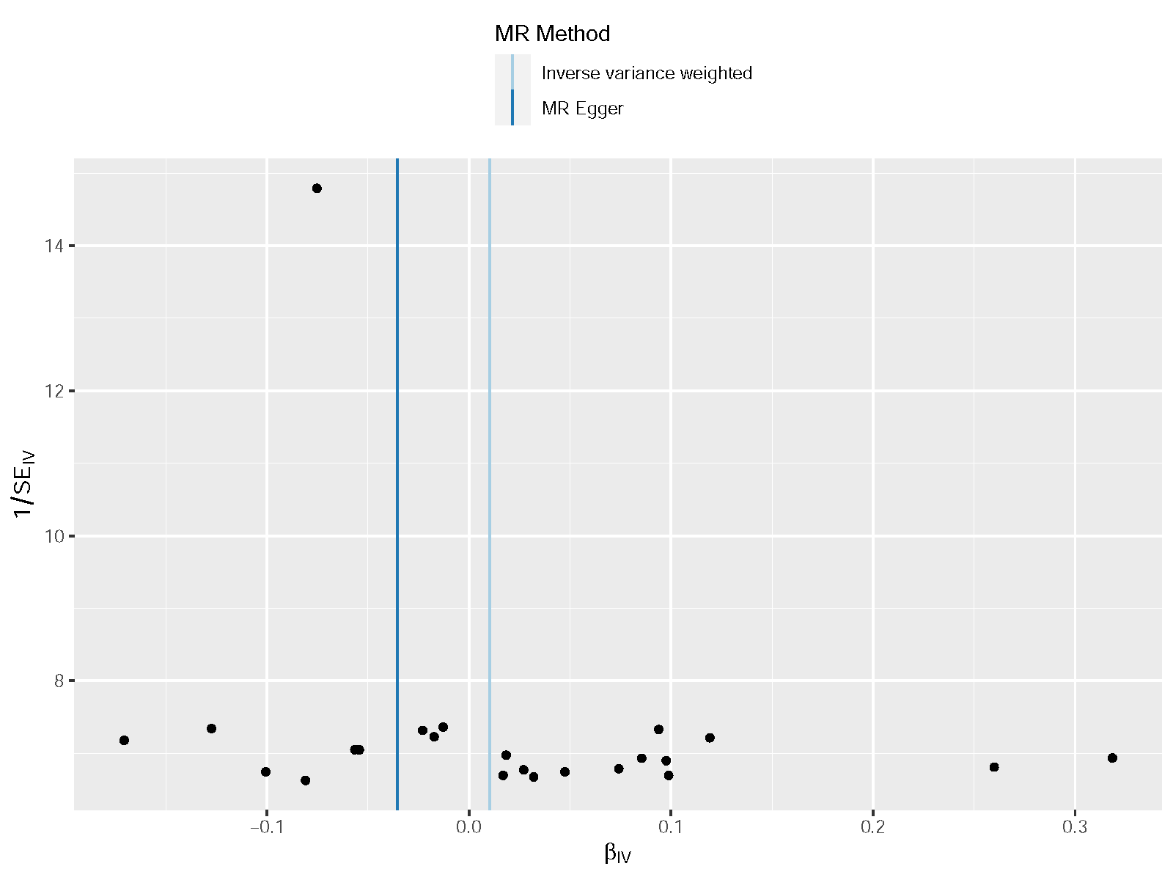

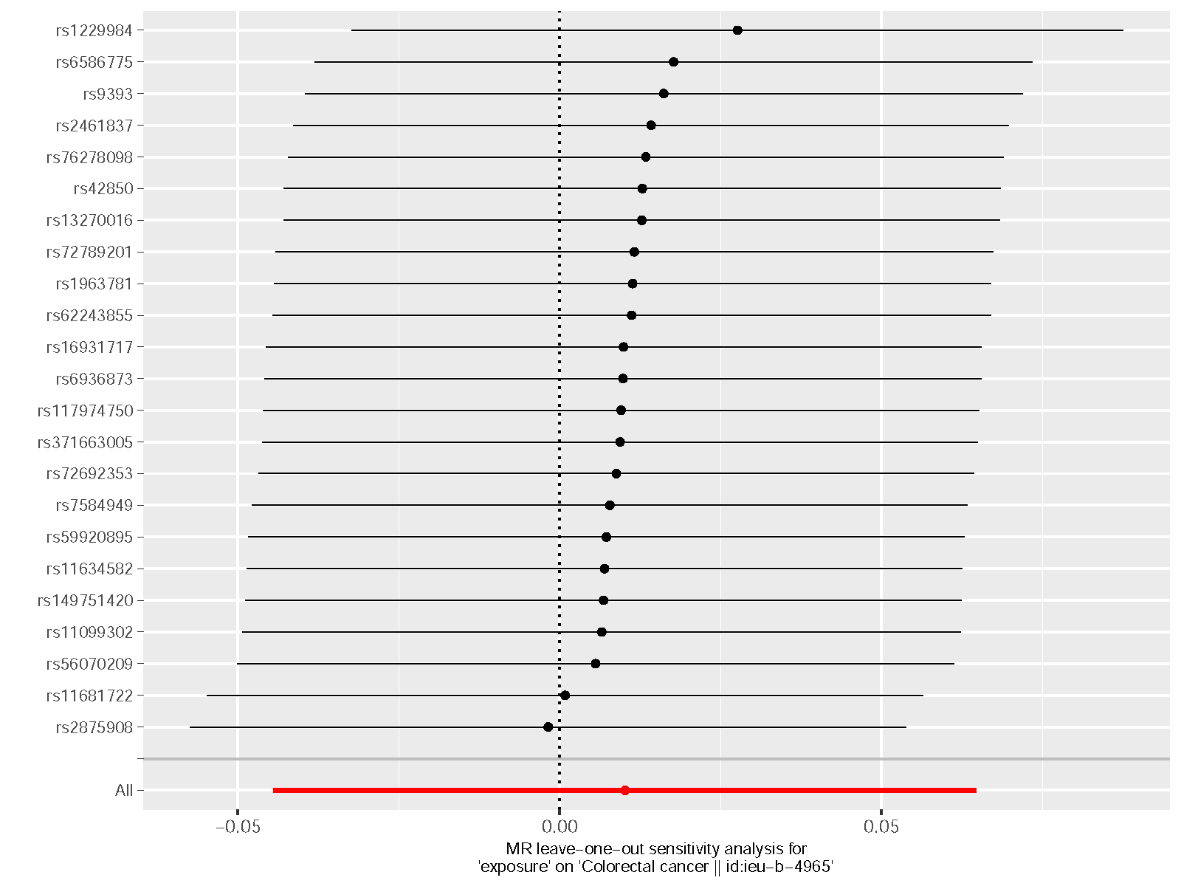


**C**

**A**

**B**

Figure S9.

A. Scatter plot of SNPs associated with Alcohol status (never) and their risk of colorectal cancer. B. Funnel plot of SNPs associated with Alcohol status (never) and their risk of colorectal cancer. C. Leave-one-out of SNPs associated with Alcohol status (never) and their risk of colorectal cancer.


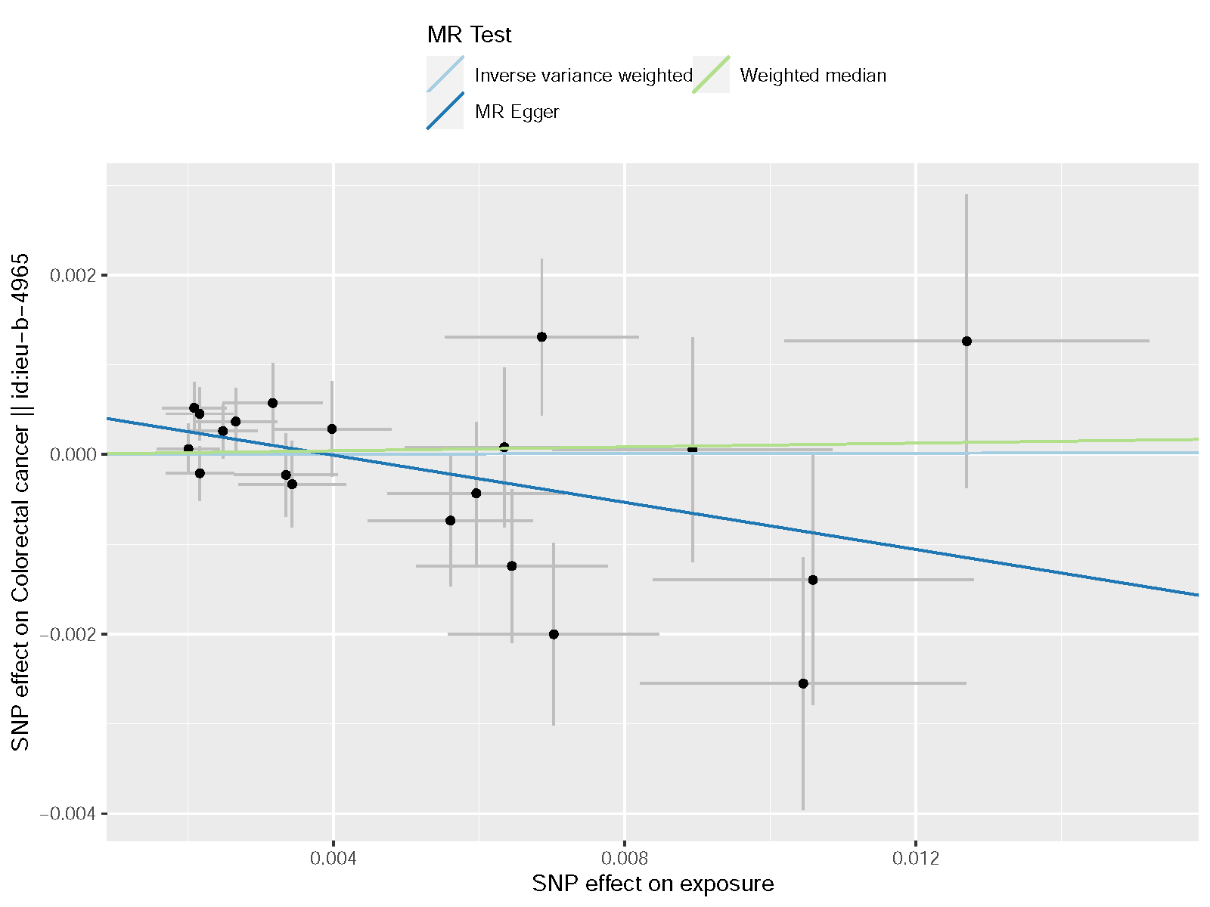

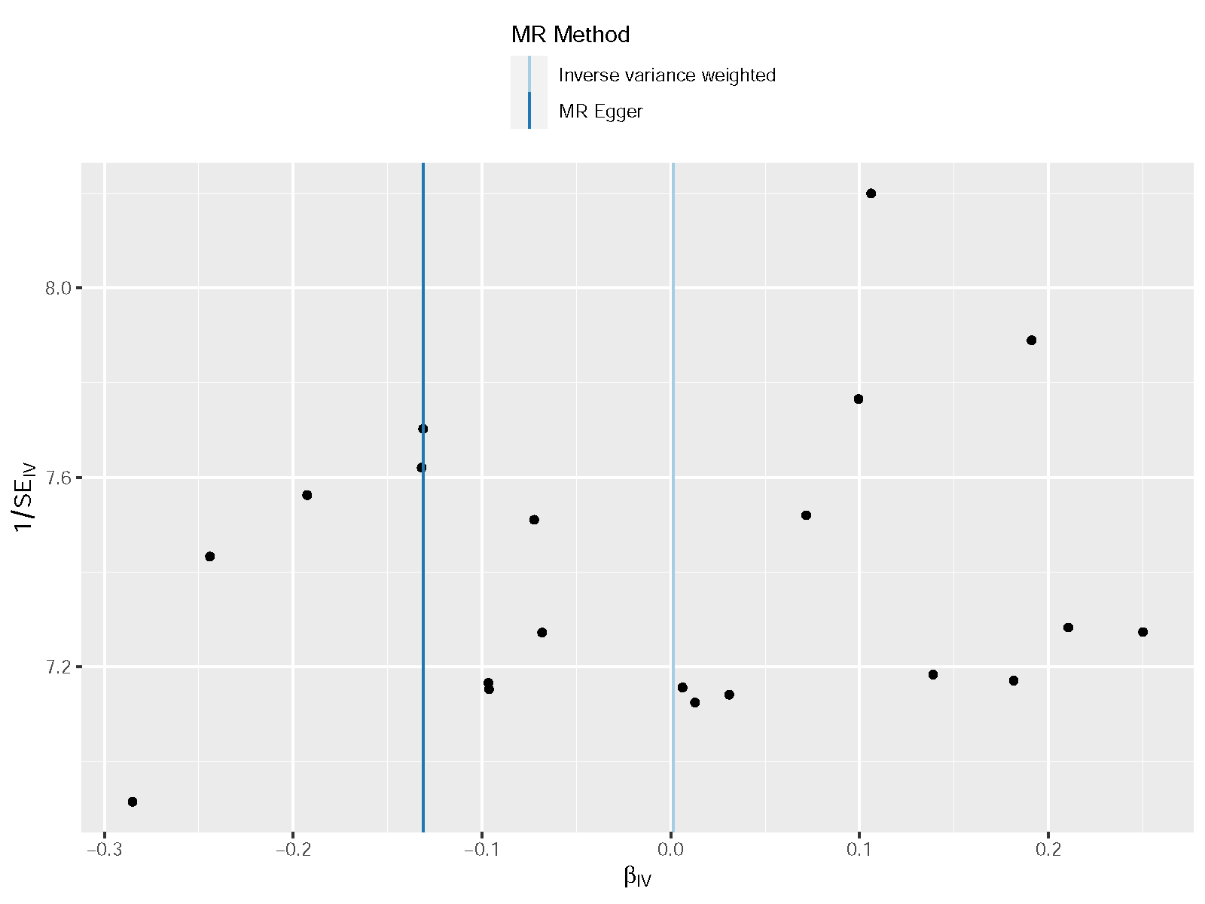

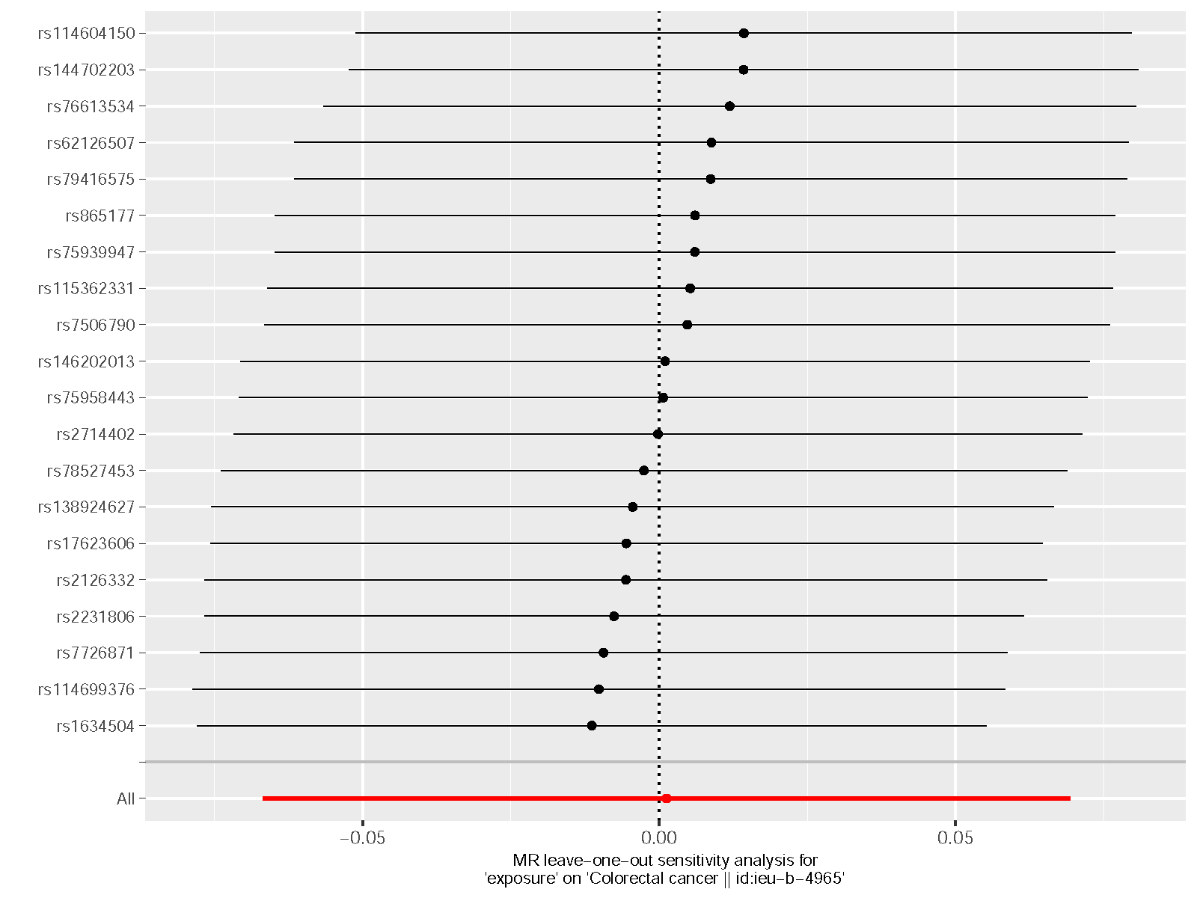


**C**

**B**

**A**

Figure S10.

A. Scatter plot of SNPs associated with Alcohol status (previous) and their risk of colorectal cancer. B. Funnel plot of SNPs associated with Alcohol status (previous) and their risk of colorectal cancer. C. Leave-one-out of SNPs associated with Alcohol status (previous) and their risk of colorectal cancer.


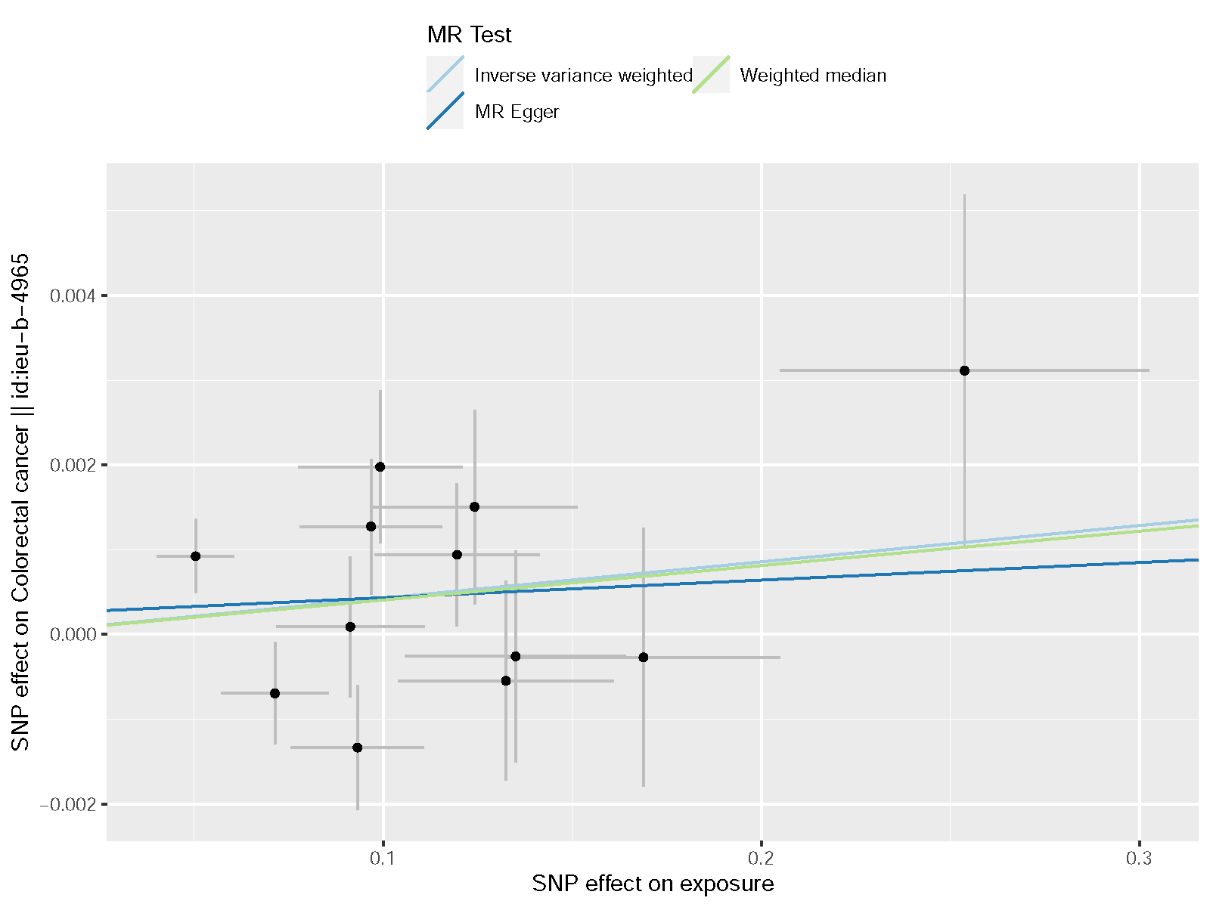

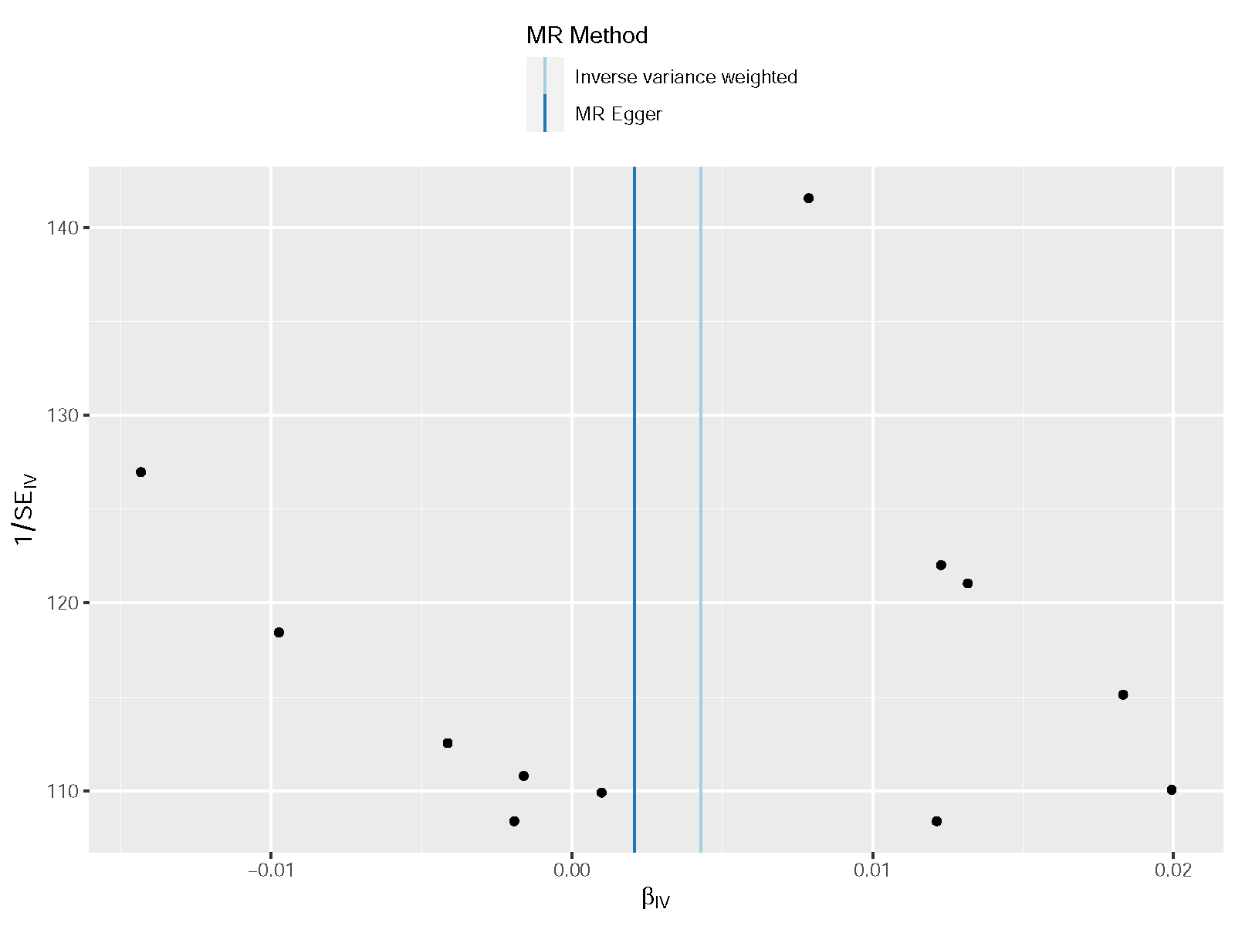

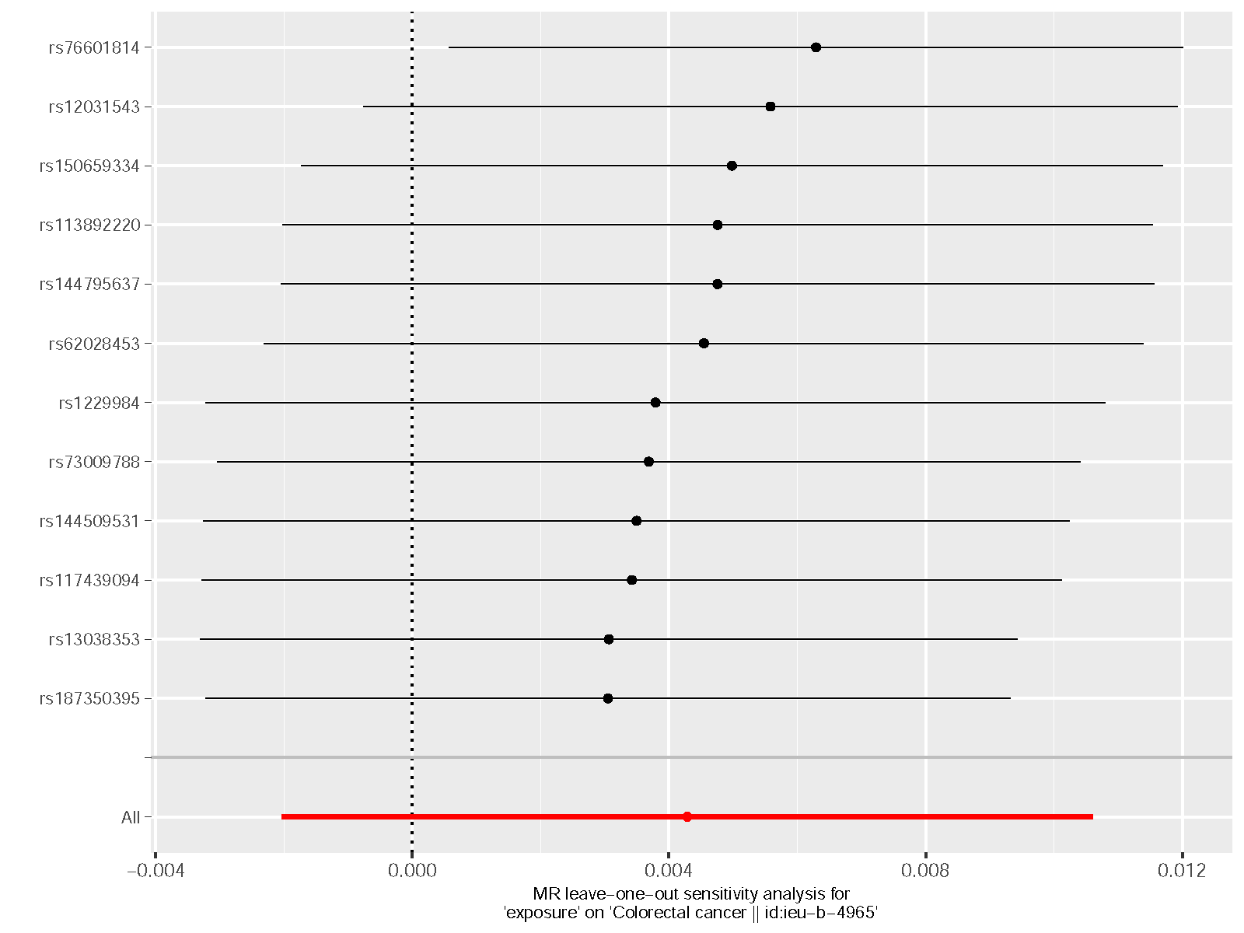


**B**

**A**

**C**

Figure S11.

A. Scatter plot of SNPs associated with Alcohol (female) and their risk of colorectal cancer. B. Funnel plot of SNPs associated with Alcohol (female) and their risk of colorectal cancer. C. Leave-one-out of SNPs associated with Alcohol (female) and their risk of colorectal cancer.


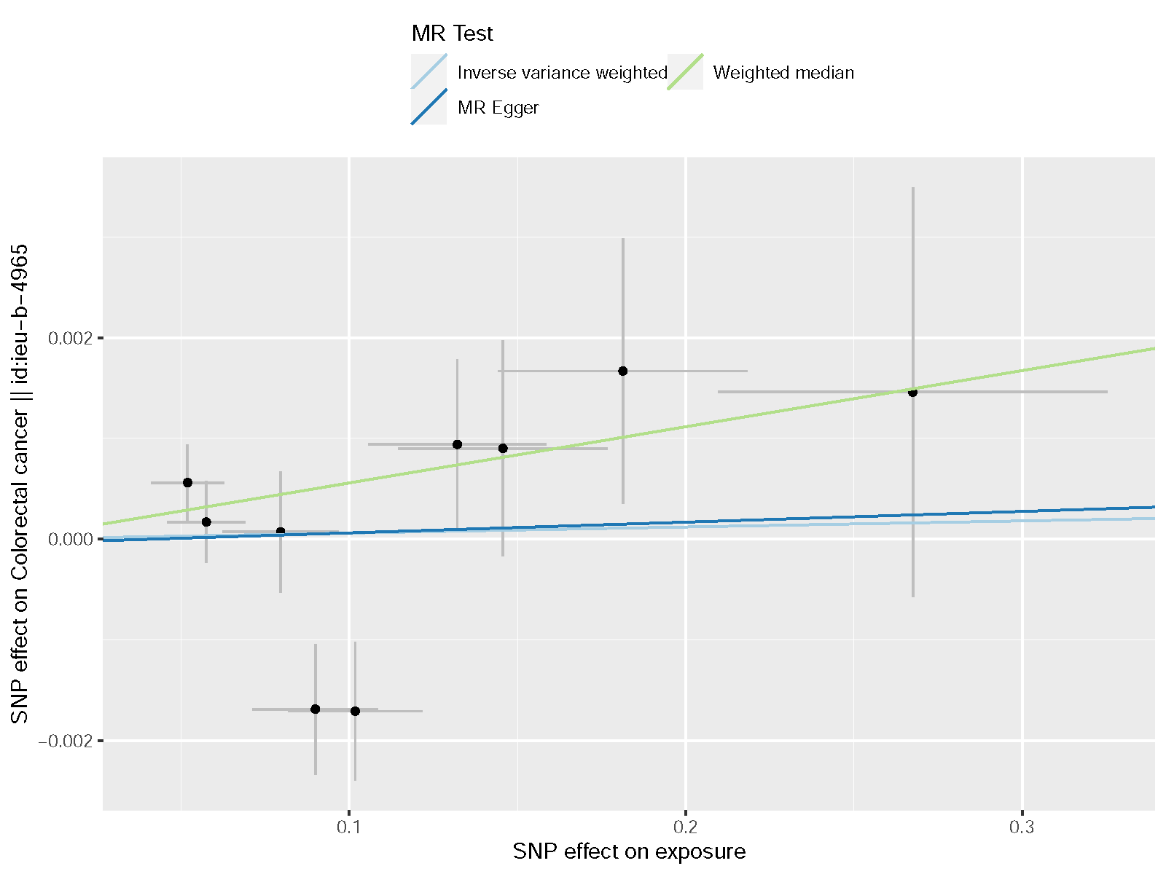

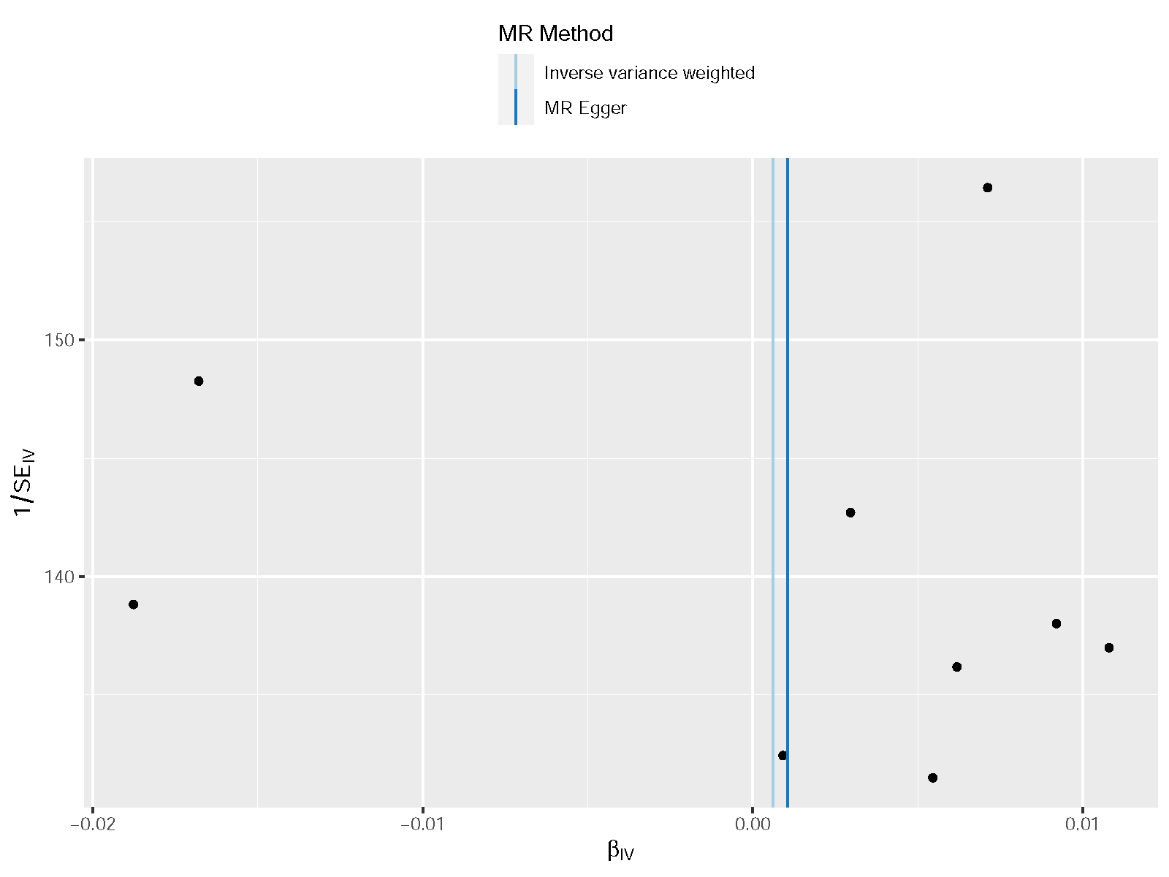

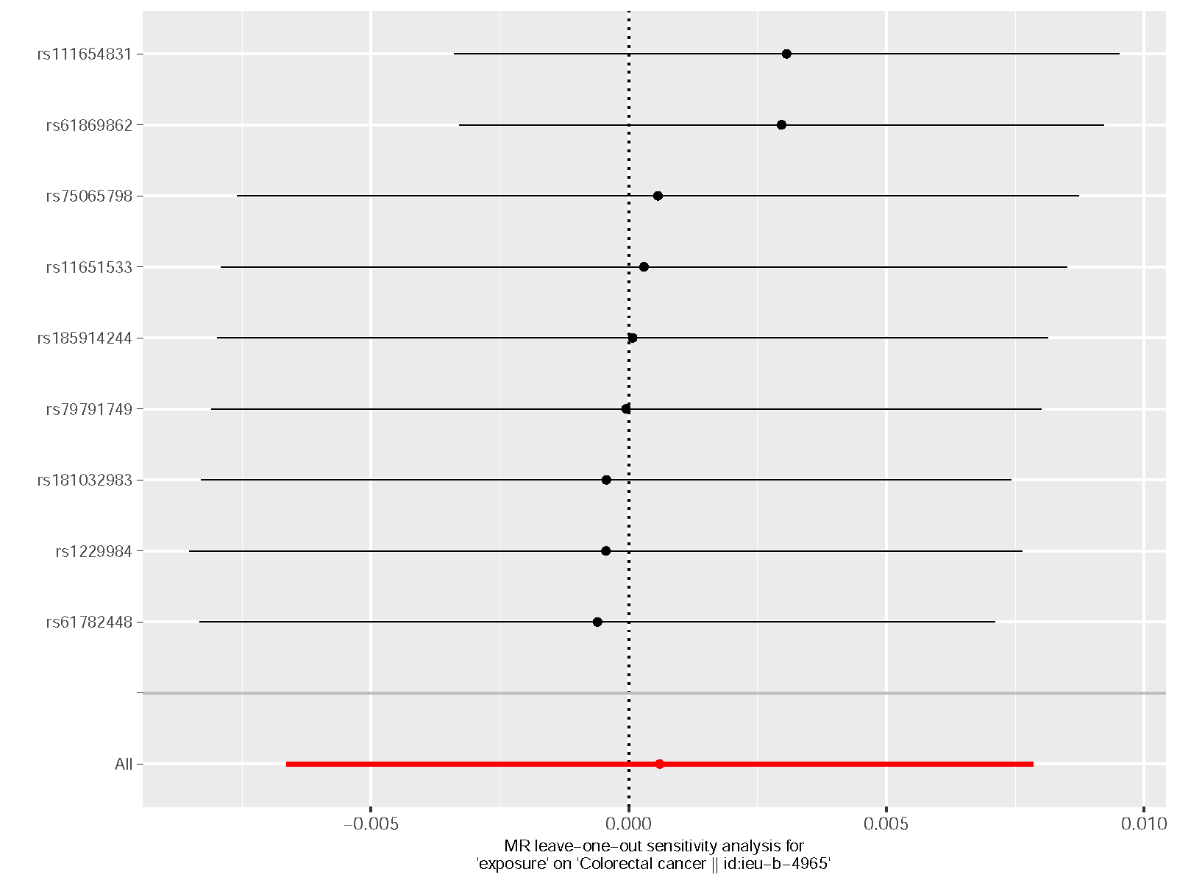


**A**

**B**

**C**

Figure S12.

A. Scatter plot of SNPs associated with Alcohol (male) and their risk of colorectal cancer. B. Funnel plot of SNPs associated with Alcohol (male) and their risk of colorectal cancer. C. Leave-one-out of SNPs associated with Alcohol (male) and their risk of colorectal cancer.


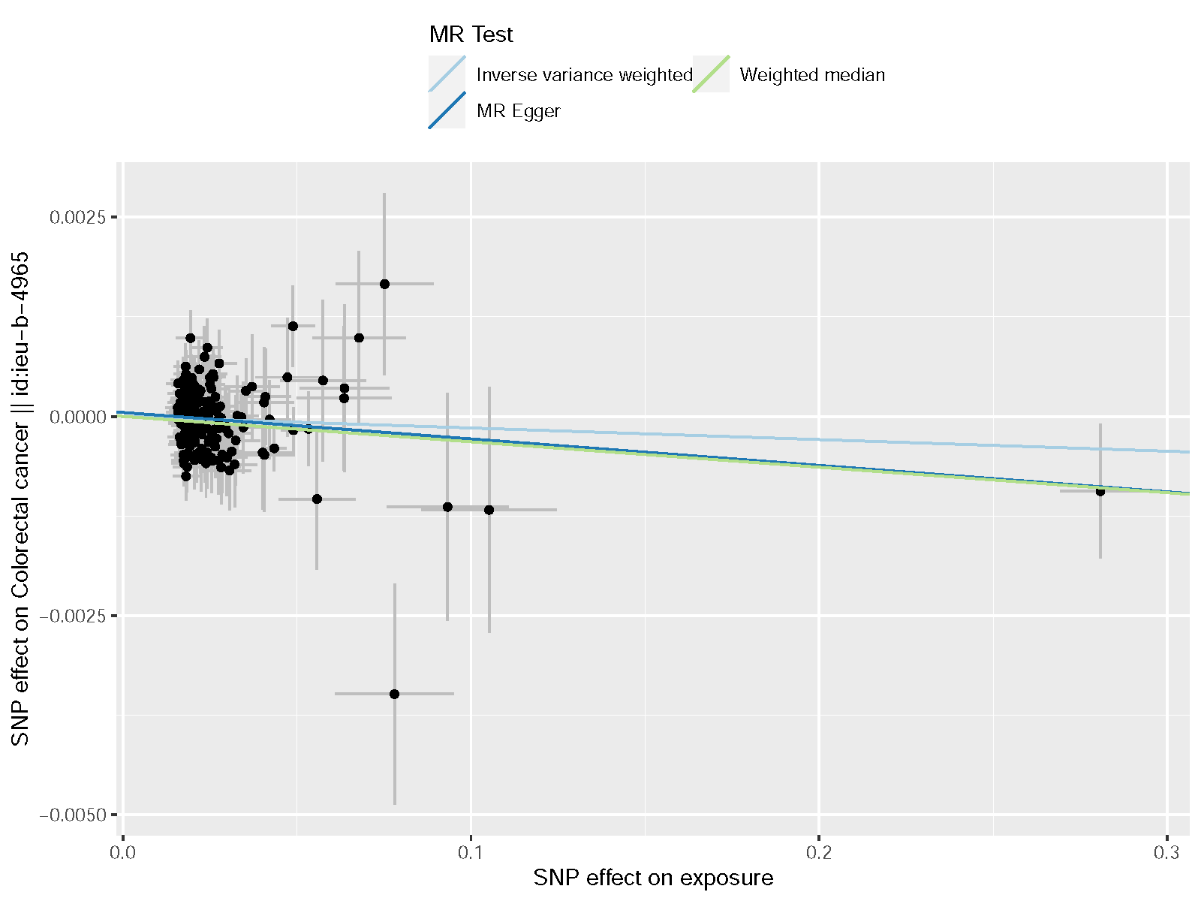

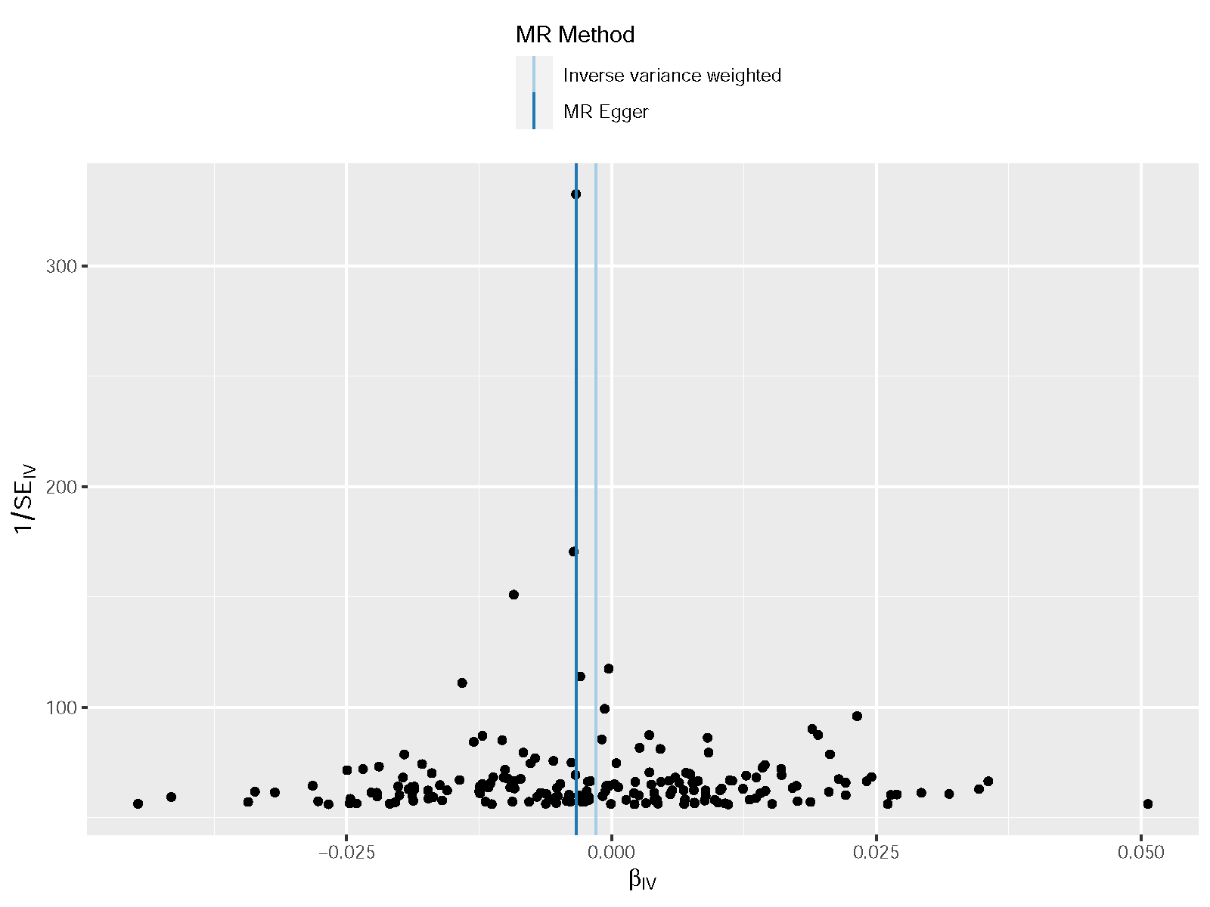

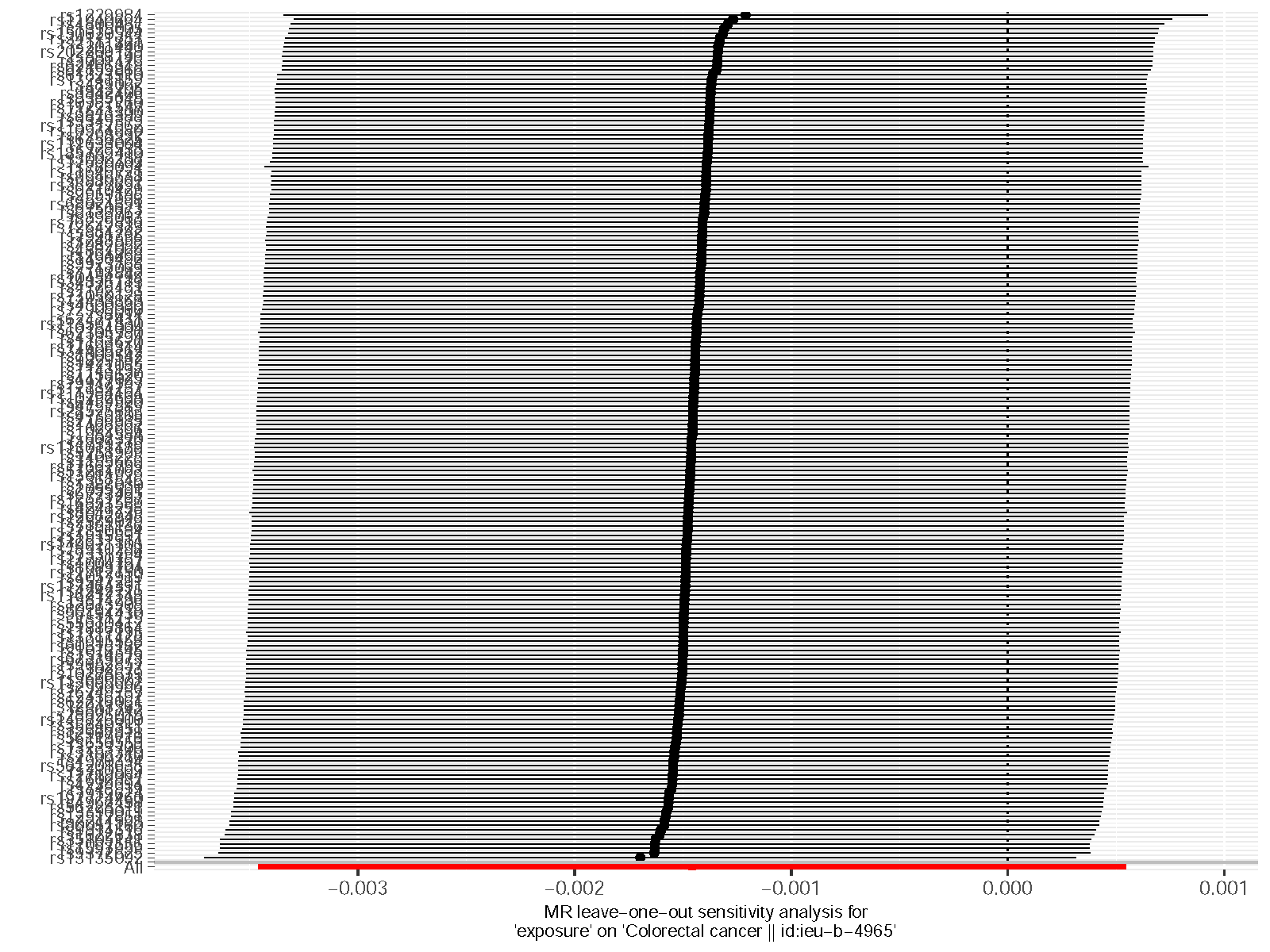


**A**

**B**

**C**

Figure S13.

A. Scatter plot of SNPs associated with Alcohol intake frequency and their risk of colorectal cancer. B. Funnel plot of SNPs associated with Alcohol intake frequency and their risk of colorectal cancer. C. Leave-one-out of SNPs associated with Alcohol intake frequency and their risk of colorectal cancer.


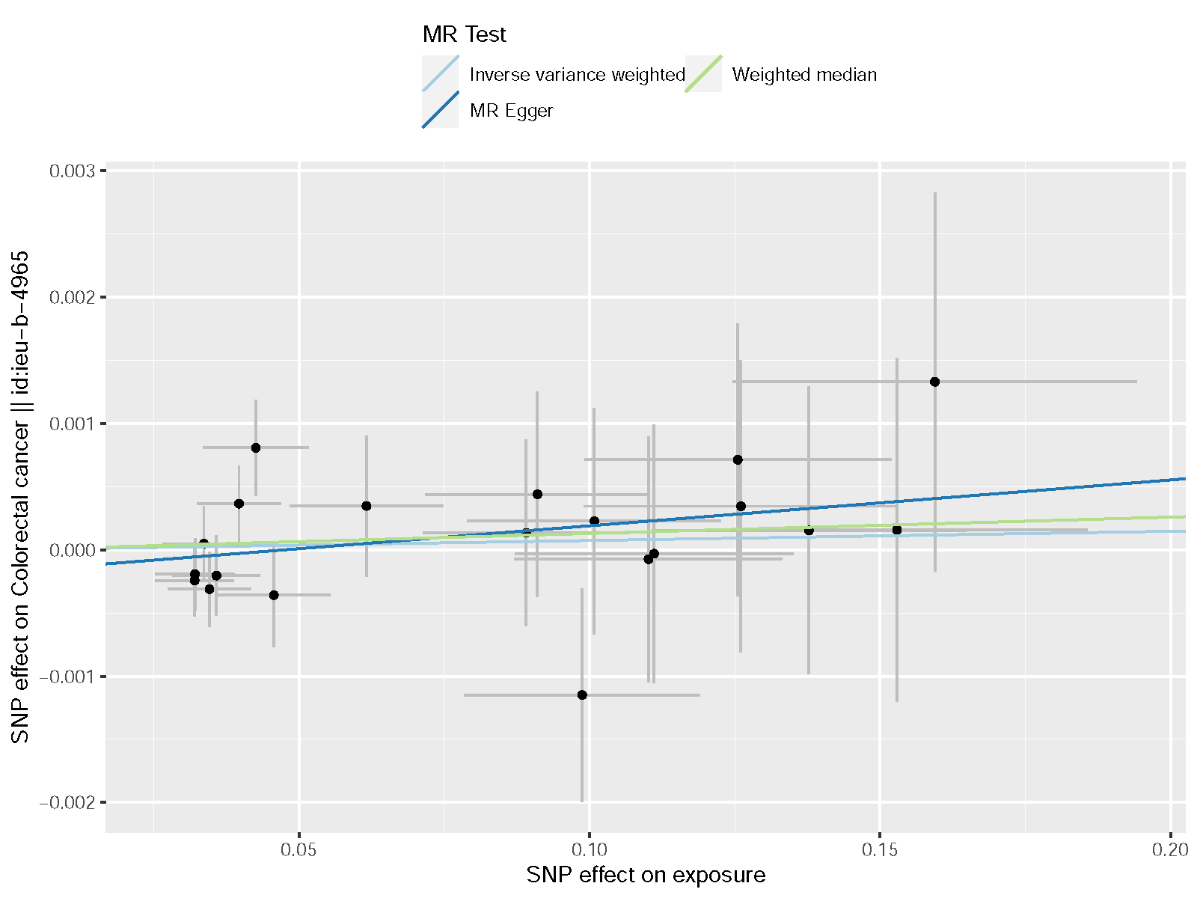

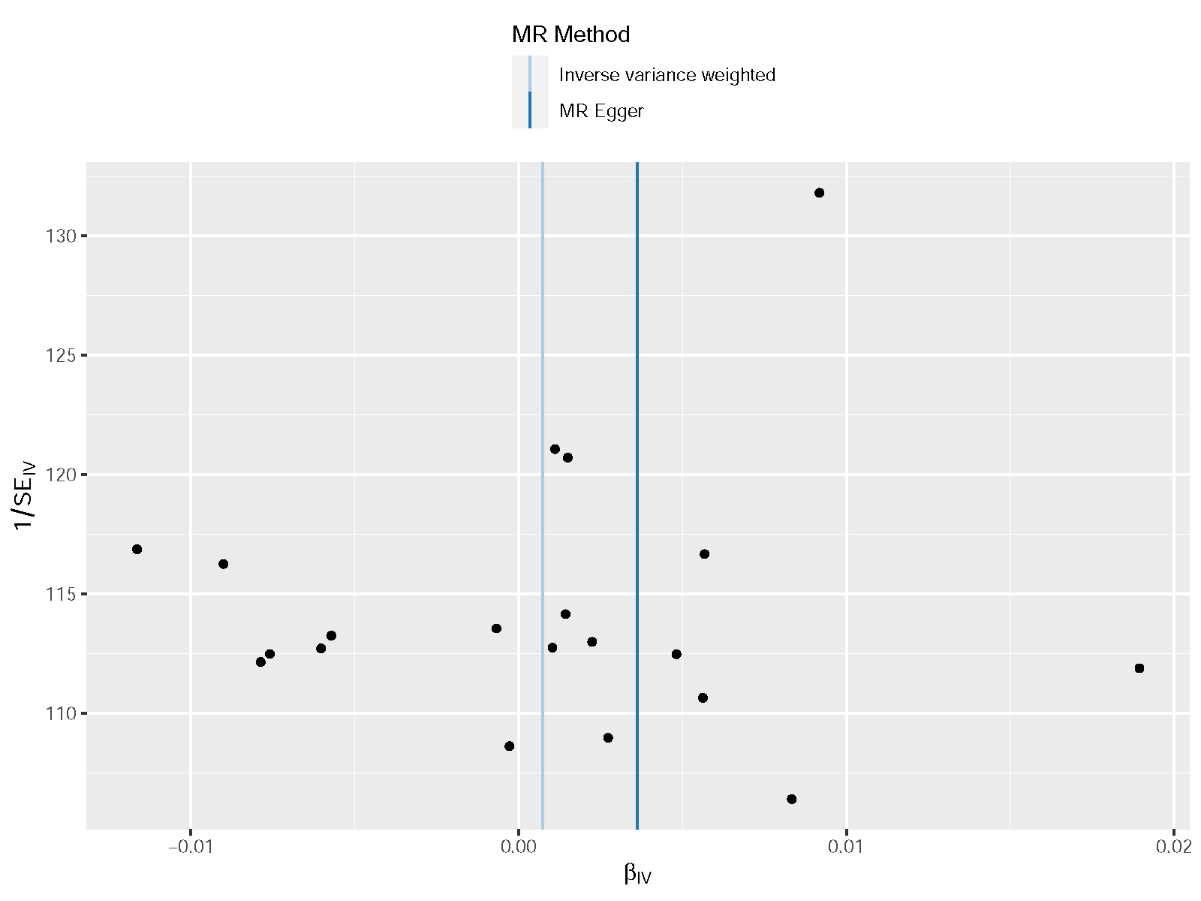

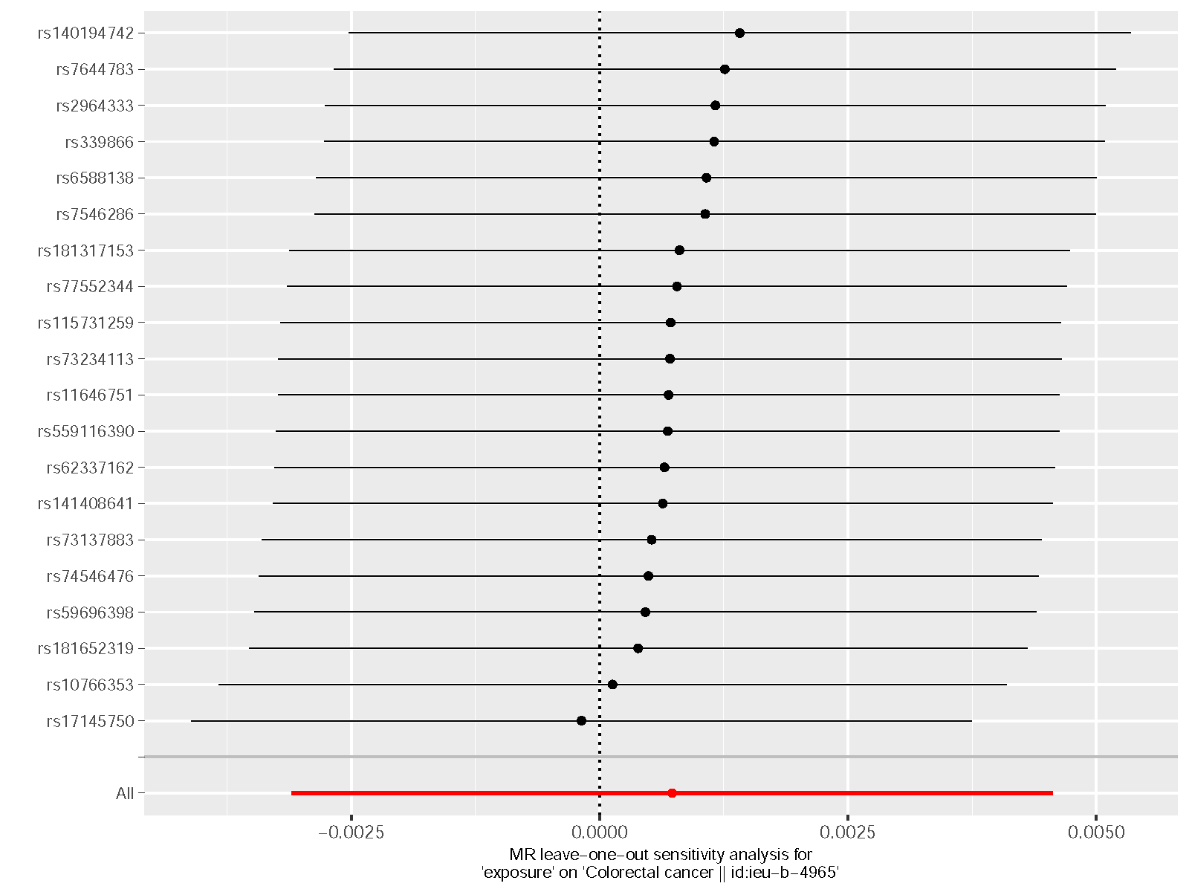


**C**

**B**

**A**

Figure S14.

A. Scatter plot of SNPs associated with White wine and their risk of colorectal cancer. B. Funnel plot of SNPs associated with White wine and their risk of colorectal cancer. C. Leave-one-out of SNPs associated with White wine and their risk of colorectal cancer.


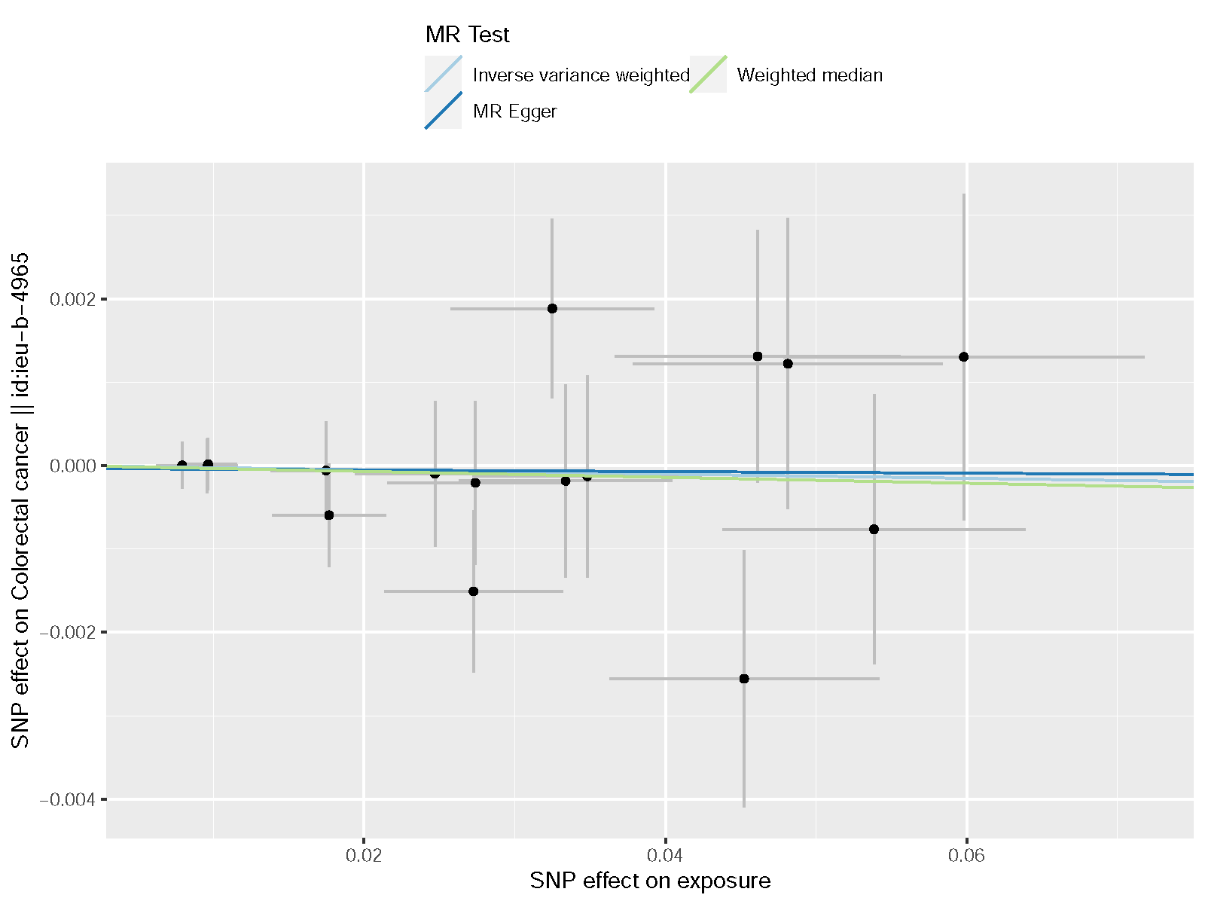

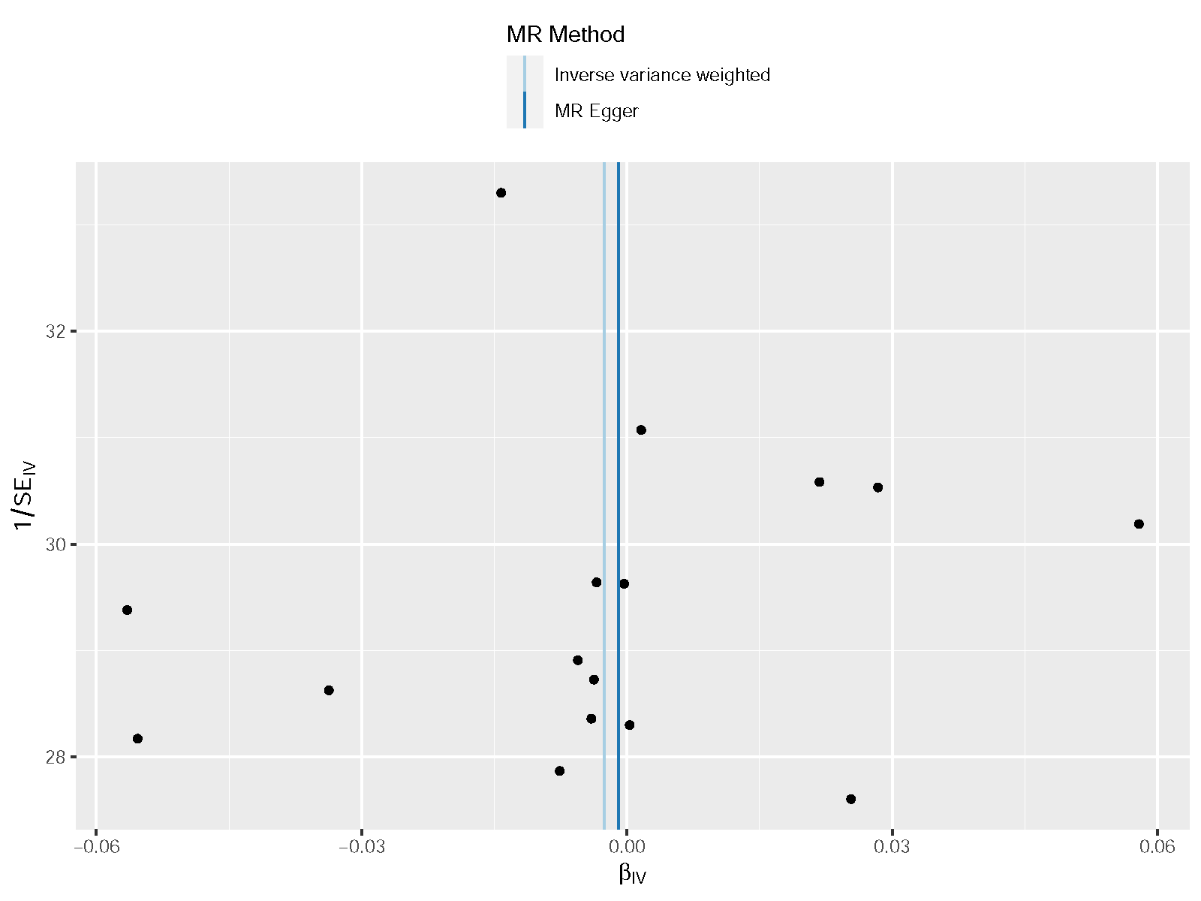

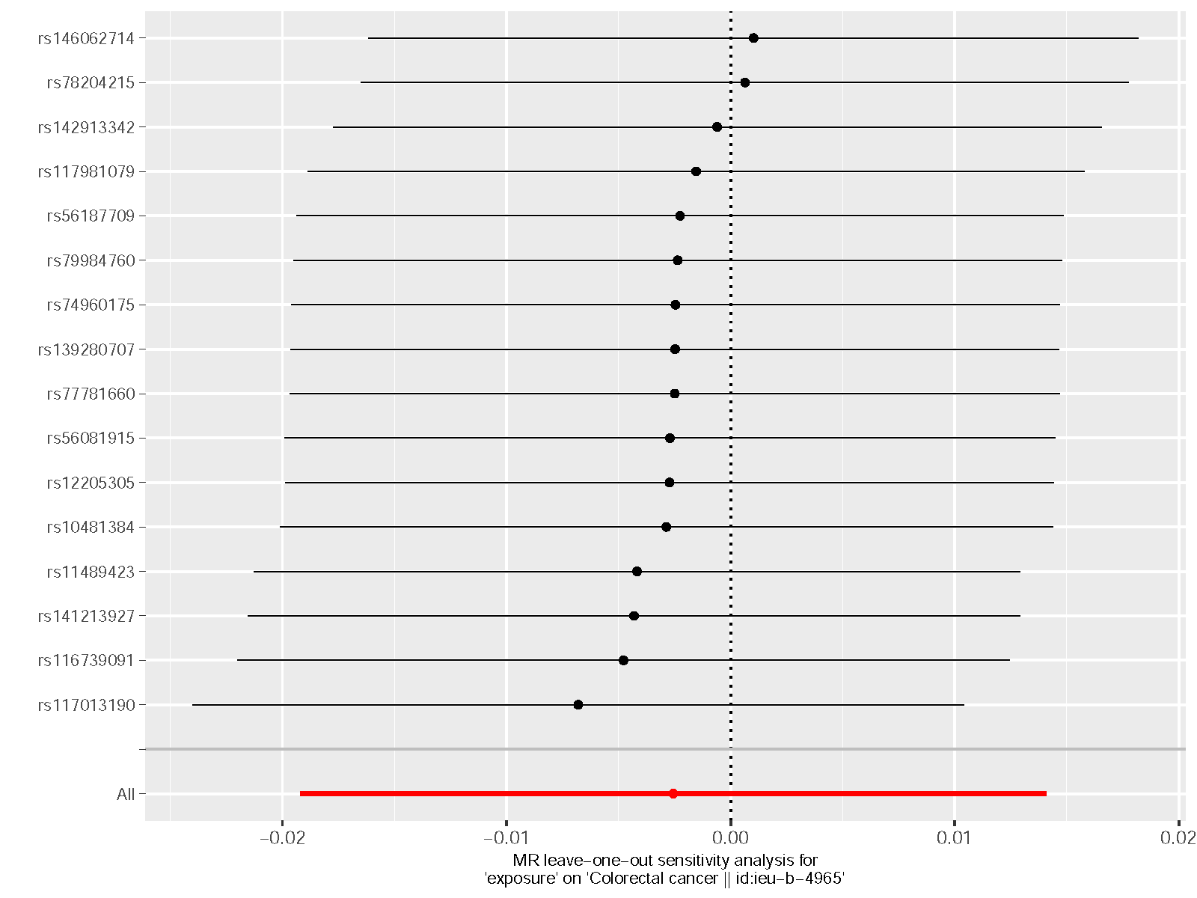


**C**

**A**

**B**

Figure S15.

A. Scatter plot of SNPs associated with Fortified wine and their risk of colorectal cancer. B. Funnel plot of SNPs associated with Fortified wine and their risk of colorectal cancer. C. Leave-one-out of SNPs associated with Fortified wine and their risk of colorectal cancer.


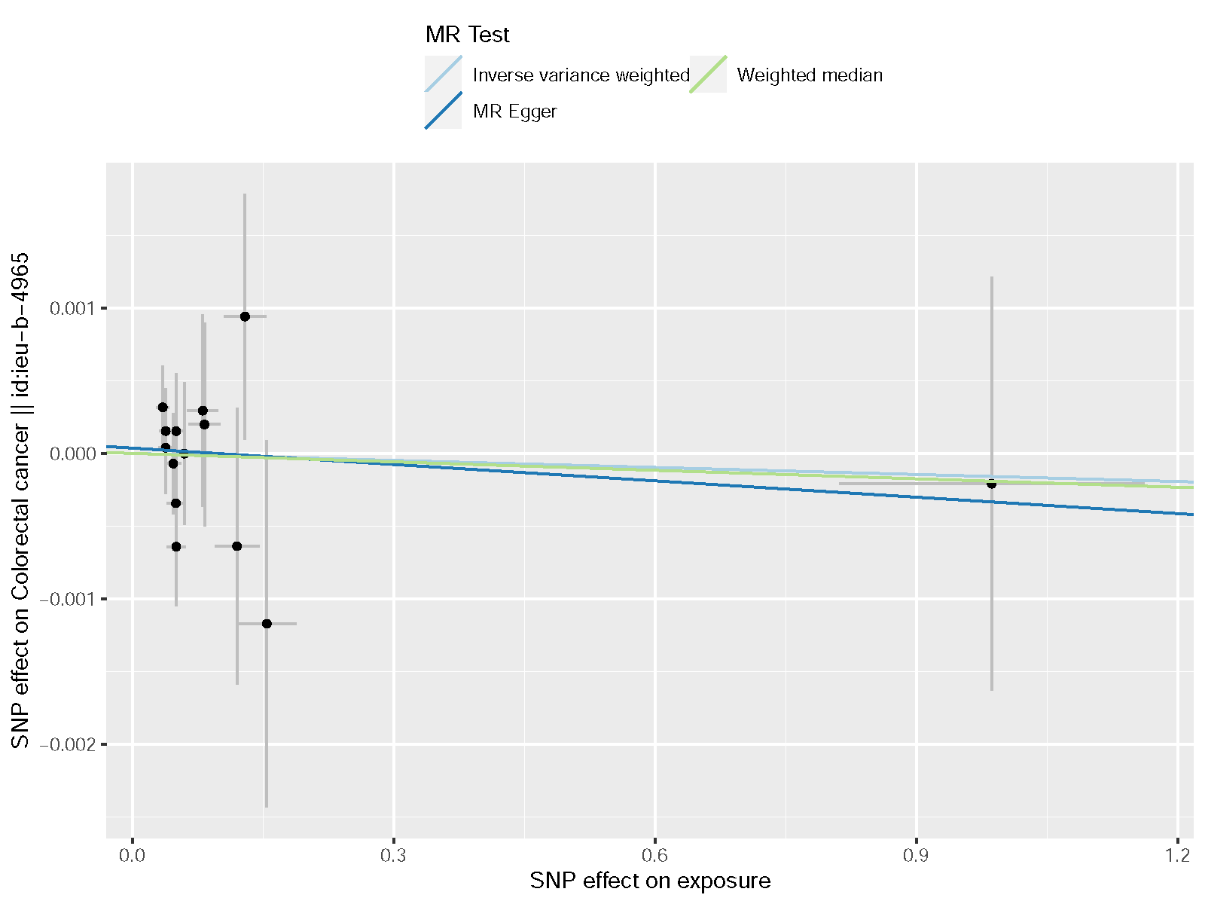

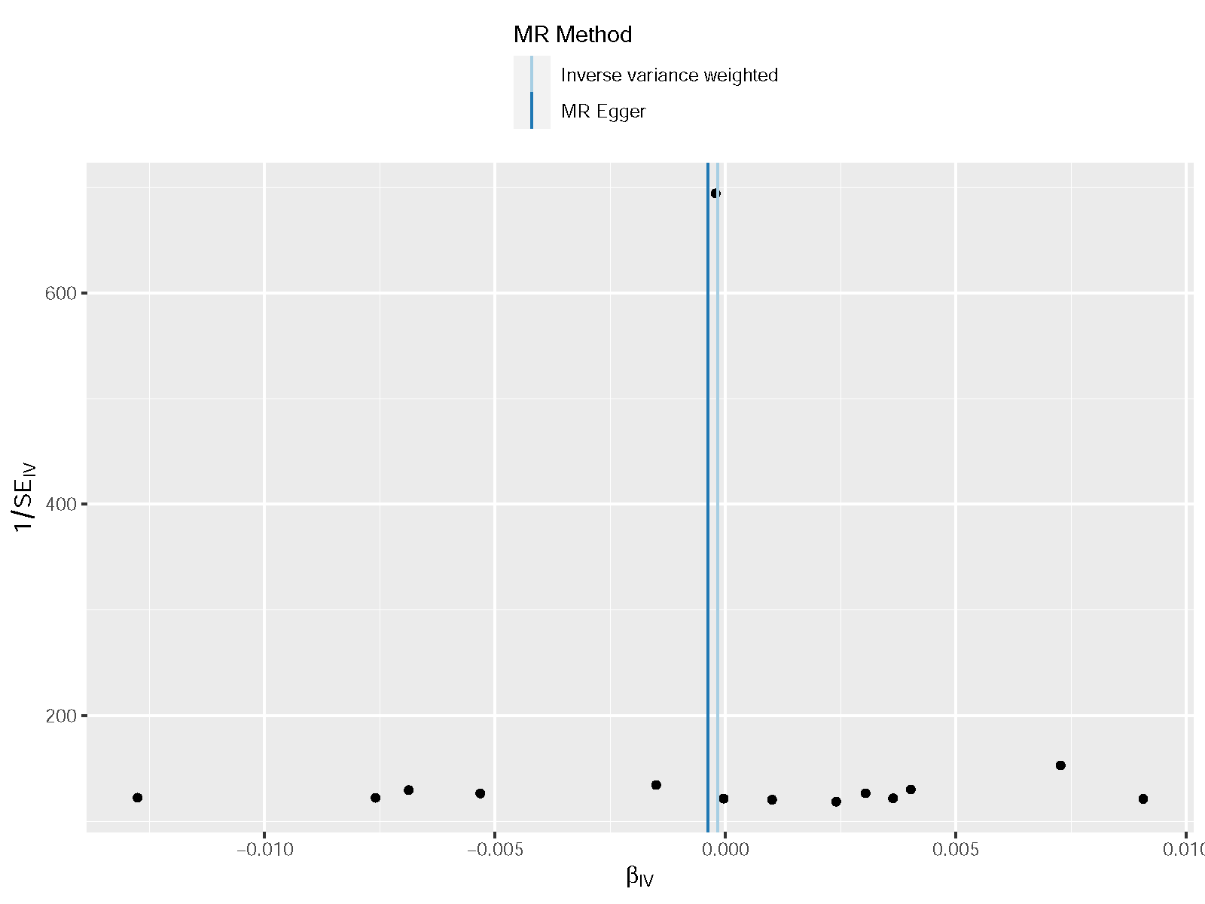

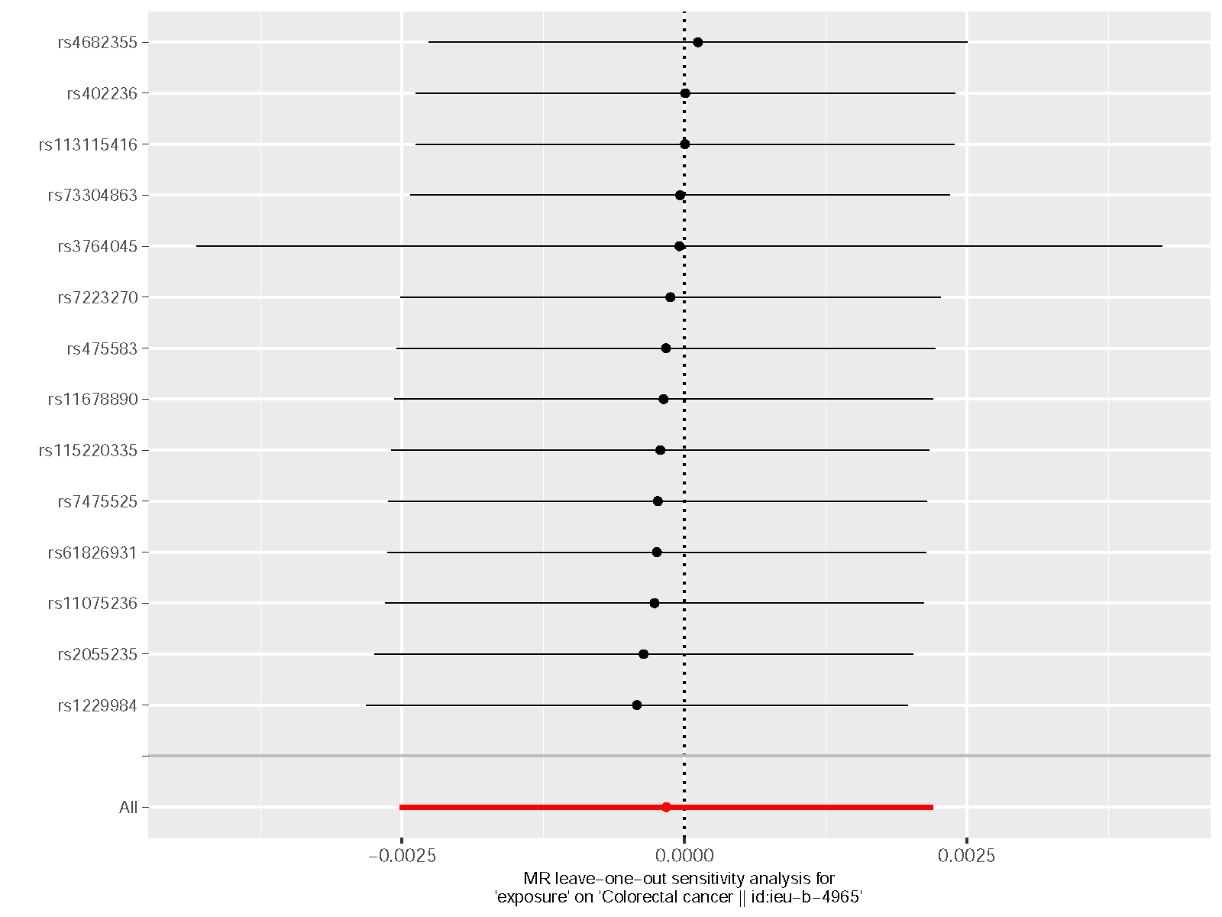


**C**

**A**

**B**

Figure S16.

A. Scatter plot of SNPs associated with Red wine and their risk of colorectal cancer. B. Funnel plot of SNPs associated with Red wine and their risk of colorectal cancer. C. Leave-one-out of SNPs associated with Red wine and their risk of colorectal cancer.


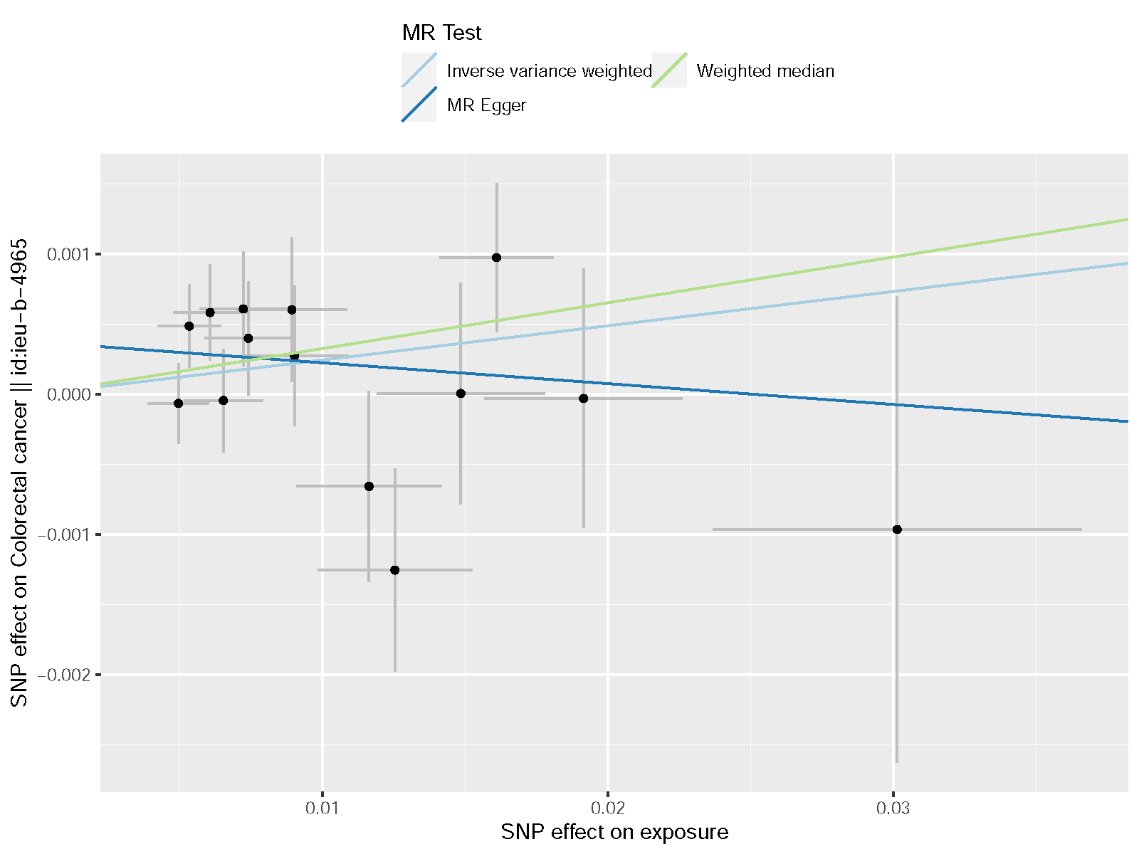

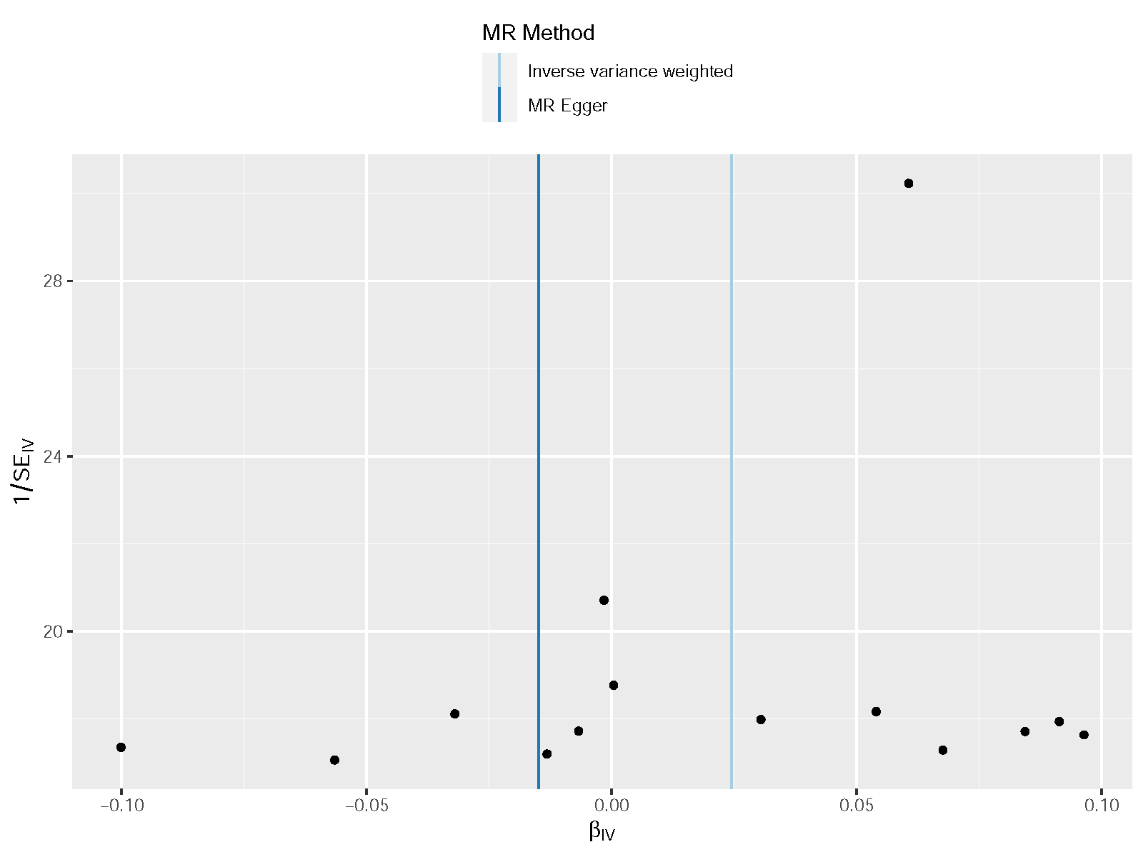

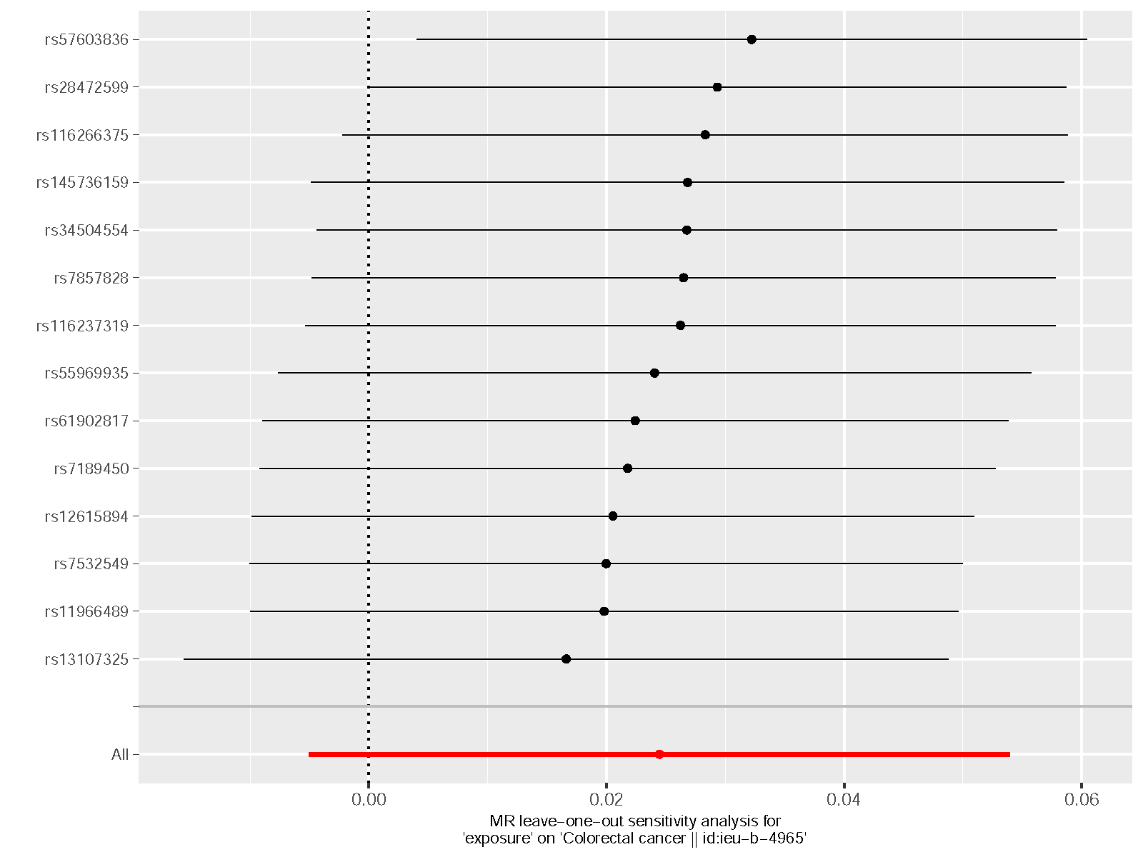


**C**

**B**

**A**

Figure S17.

A. Scatter plot of SNPs associated with Decaffeinated coffee and their risk of colorectal cancer. B. Funnel plot of SNPs associated with Decaffeinated coffee and their risk of colorectal cancer. C. Leave-one-out of SNPs associated with Decaffeinated coffee and their risk of colorectal cancer.


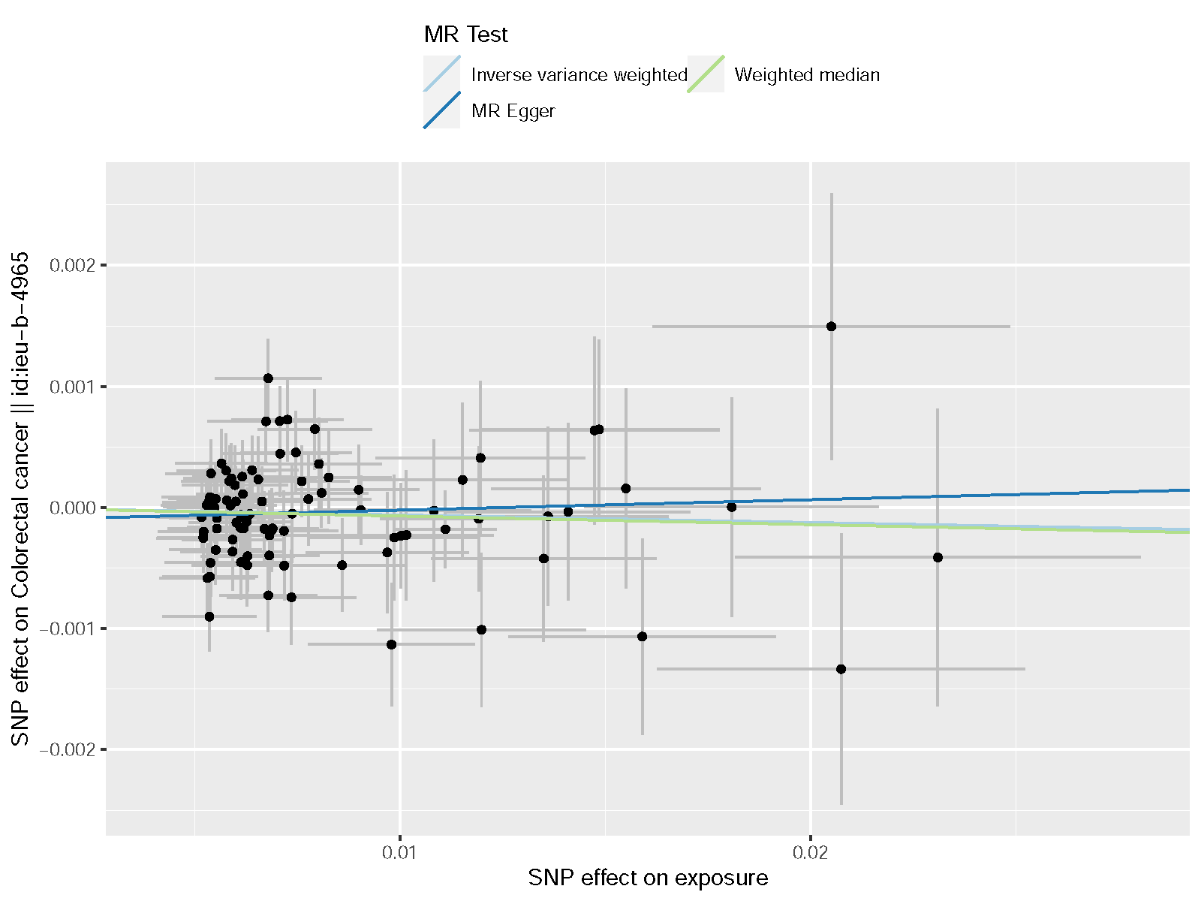

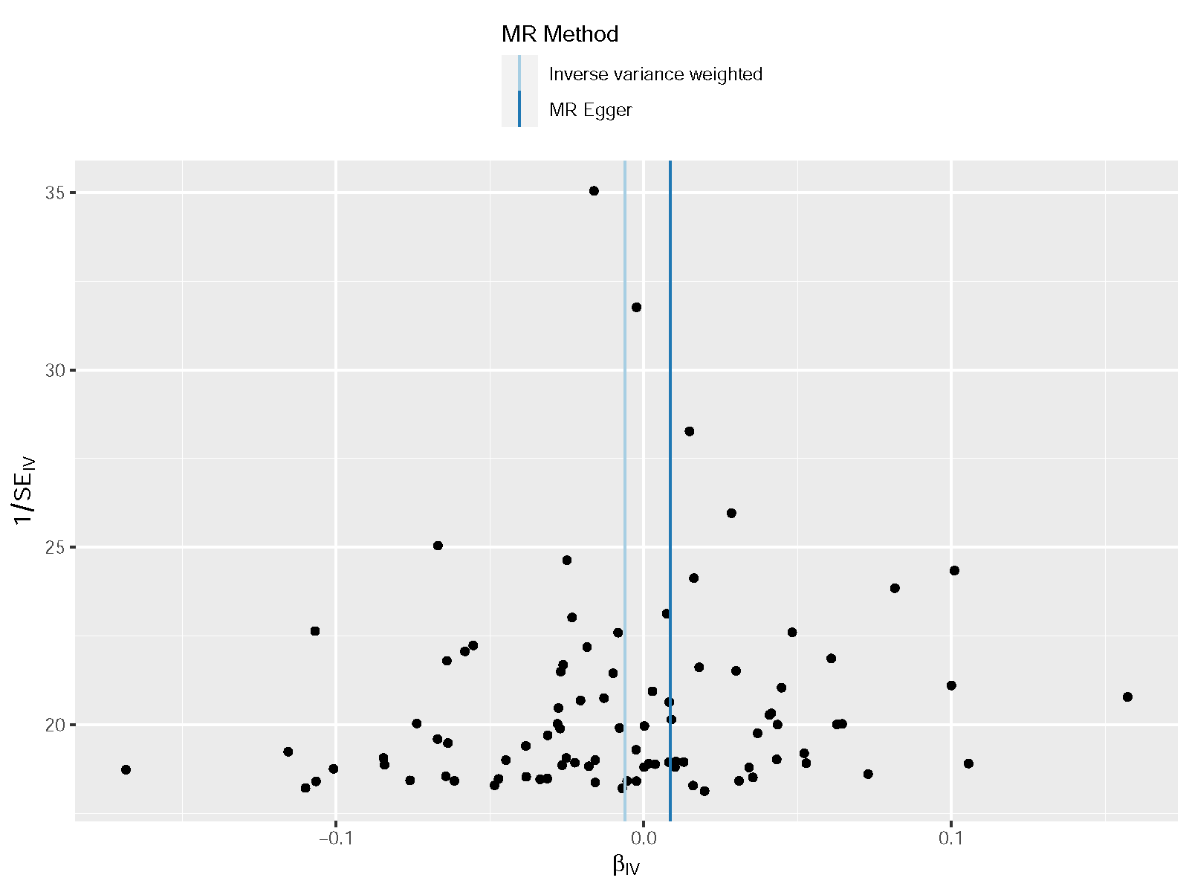

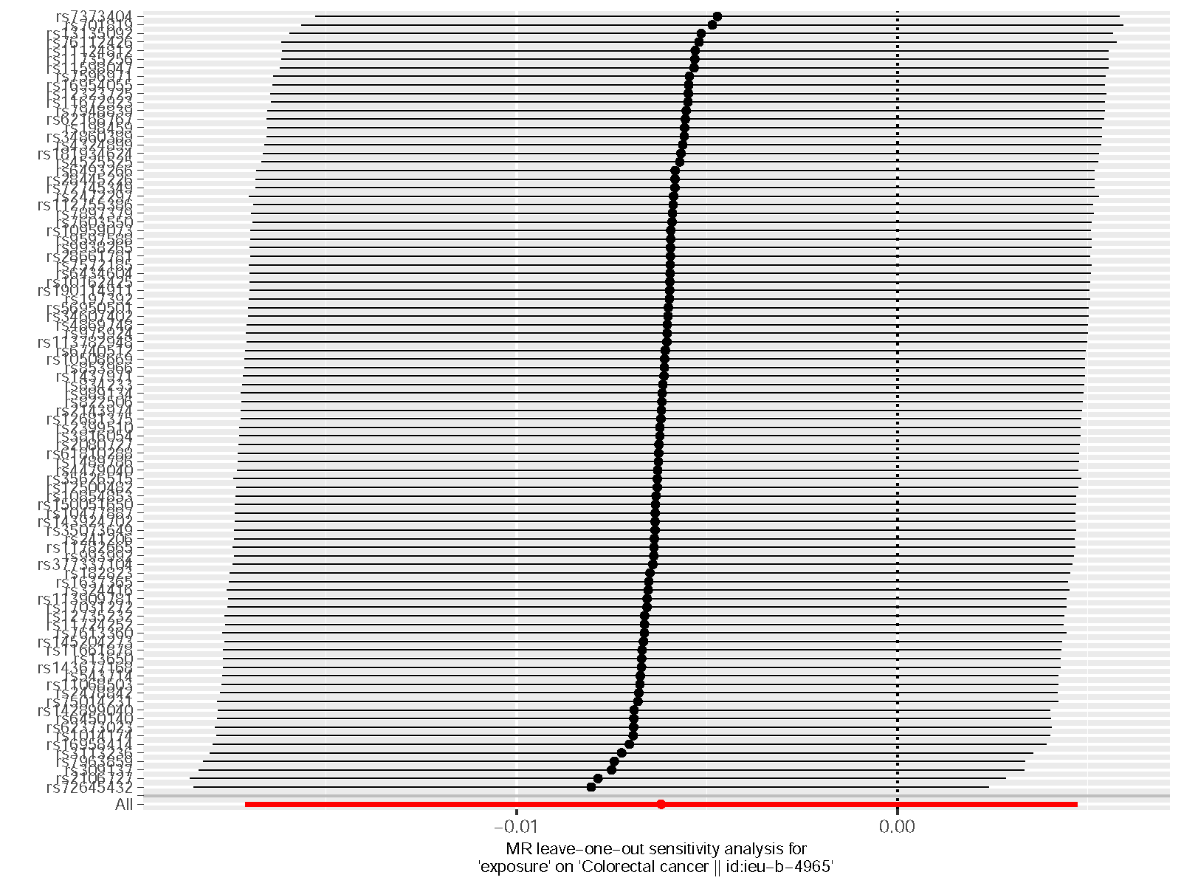


**C**

**B**

**A**

Figure S18.

A. Scatter plot of SNPs associated with Ground coffee and their risk of colorectal cancer. B. Funnel plot of SNPs associated with Ground coffee and their risk of colorectal cancer. C. Leave-one-out of SNPs associated with Ground coffee and their risk of colorectal cancer.


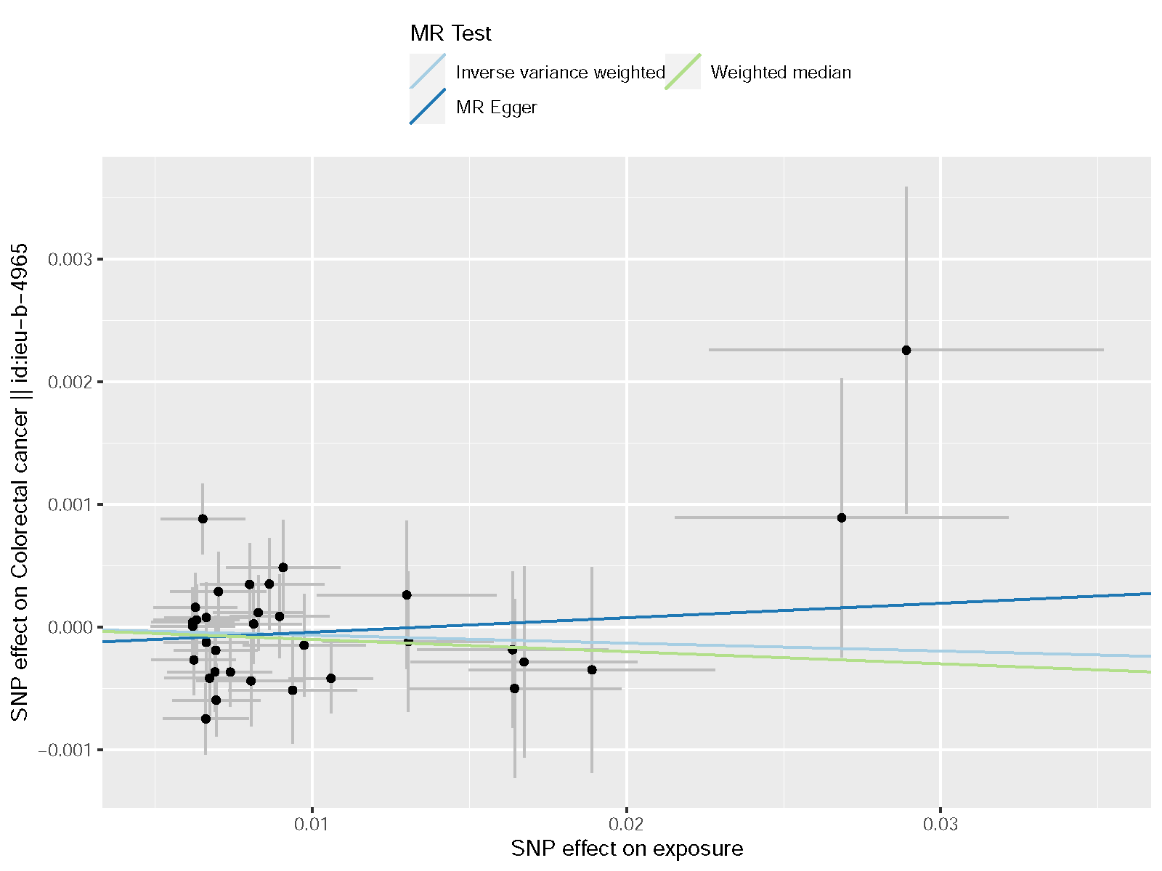

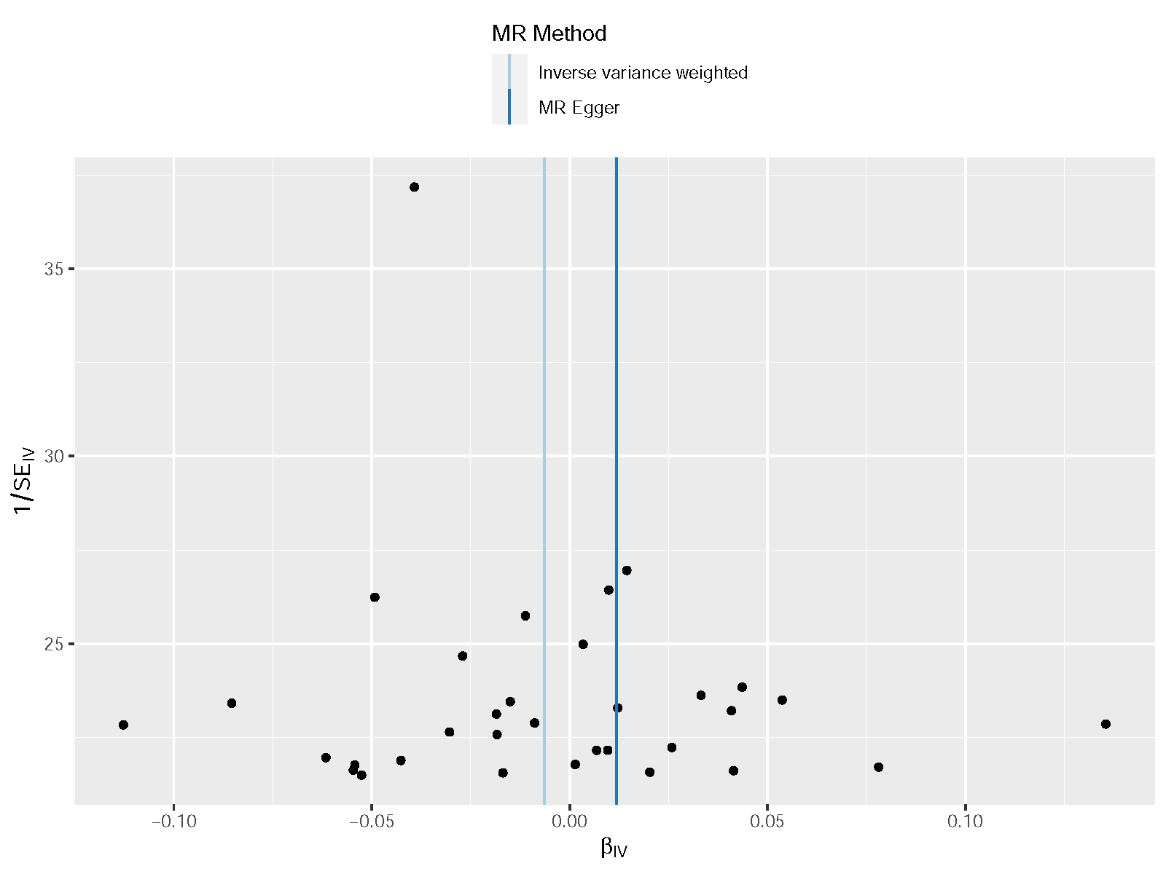

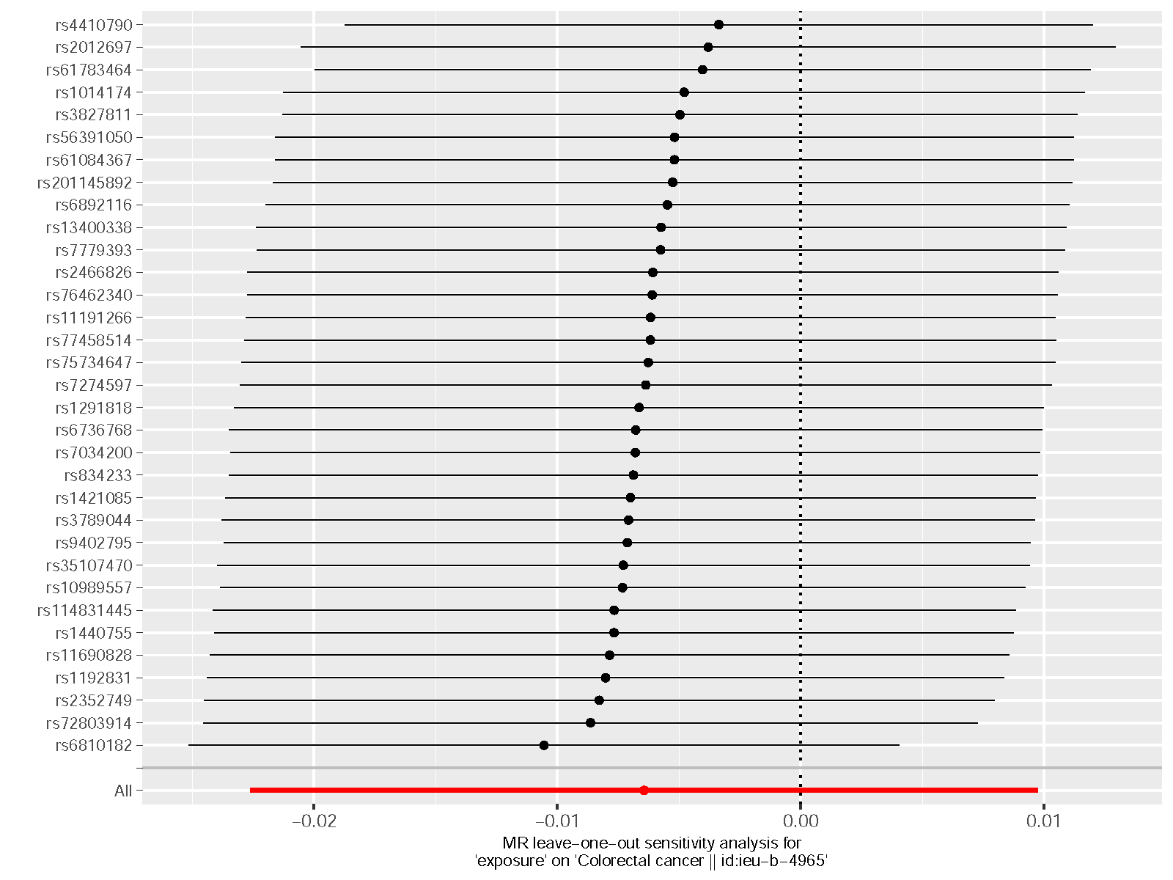


**C**

**A**

**B**

Figure S19.

A. Scatter plot of SNPs associated with Instant coffee and their risk of colorectal cancer. B. Funnel plot of SNPs associated with Instant coffee and their risk of colorectal cancer. C. Leave-one-out of SNPs associated with Instant coffee and their risk of colorectal cancer.


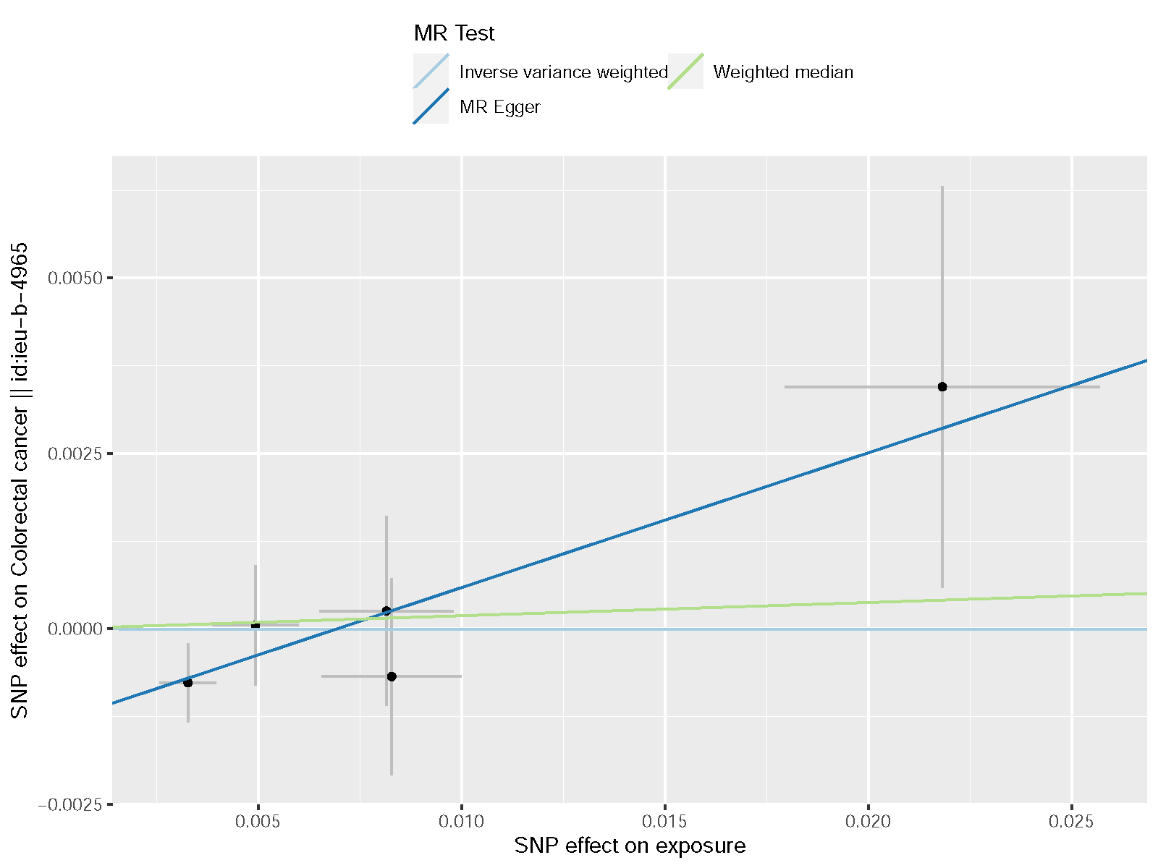

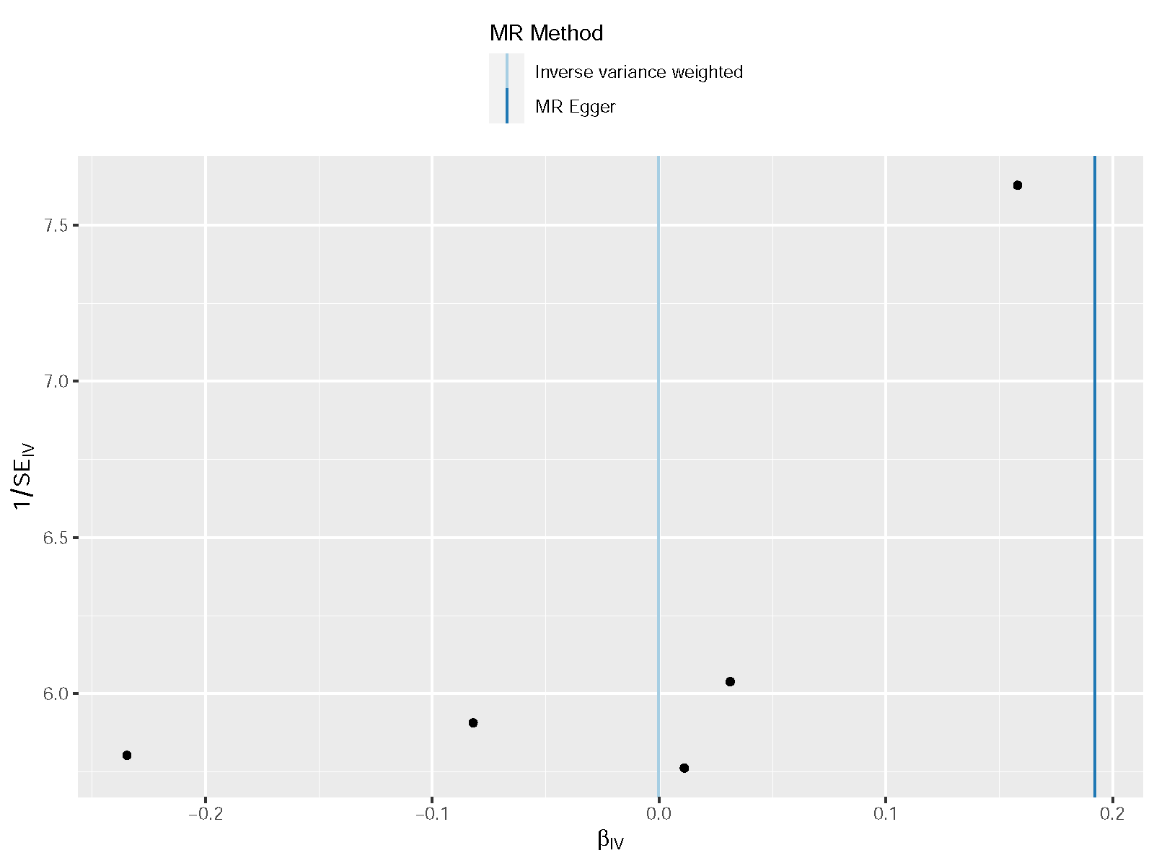

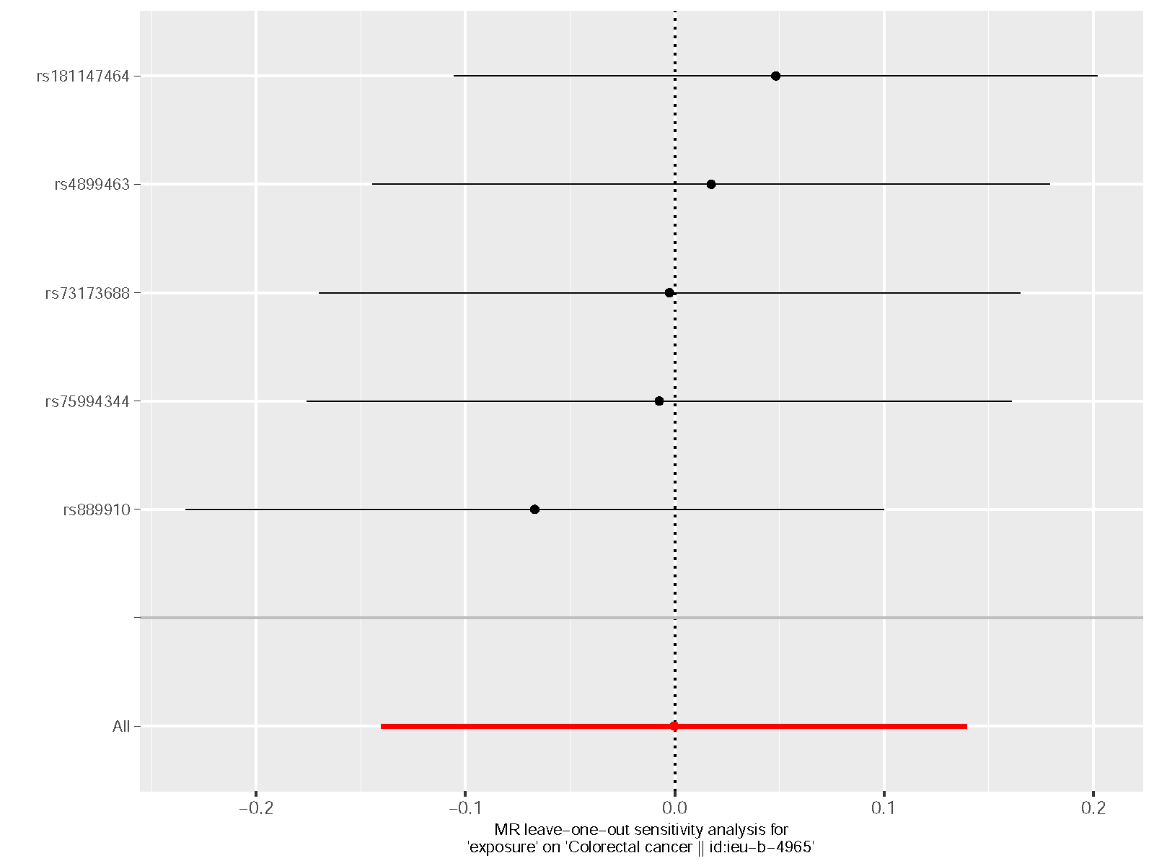


**C**

**B**

**A**

Figure S20.

A. Scatter plot of SNPs associated with Other type of coffee and their risk of colorectal cancer. B. Funnel plot of SNPs associated with Other type of coffee and their risk of colorectal cancer. C. Leave-one-out of SNPs associated with Other type of coffee and their risk of colorectal cancer.


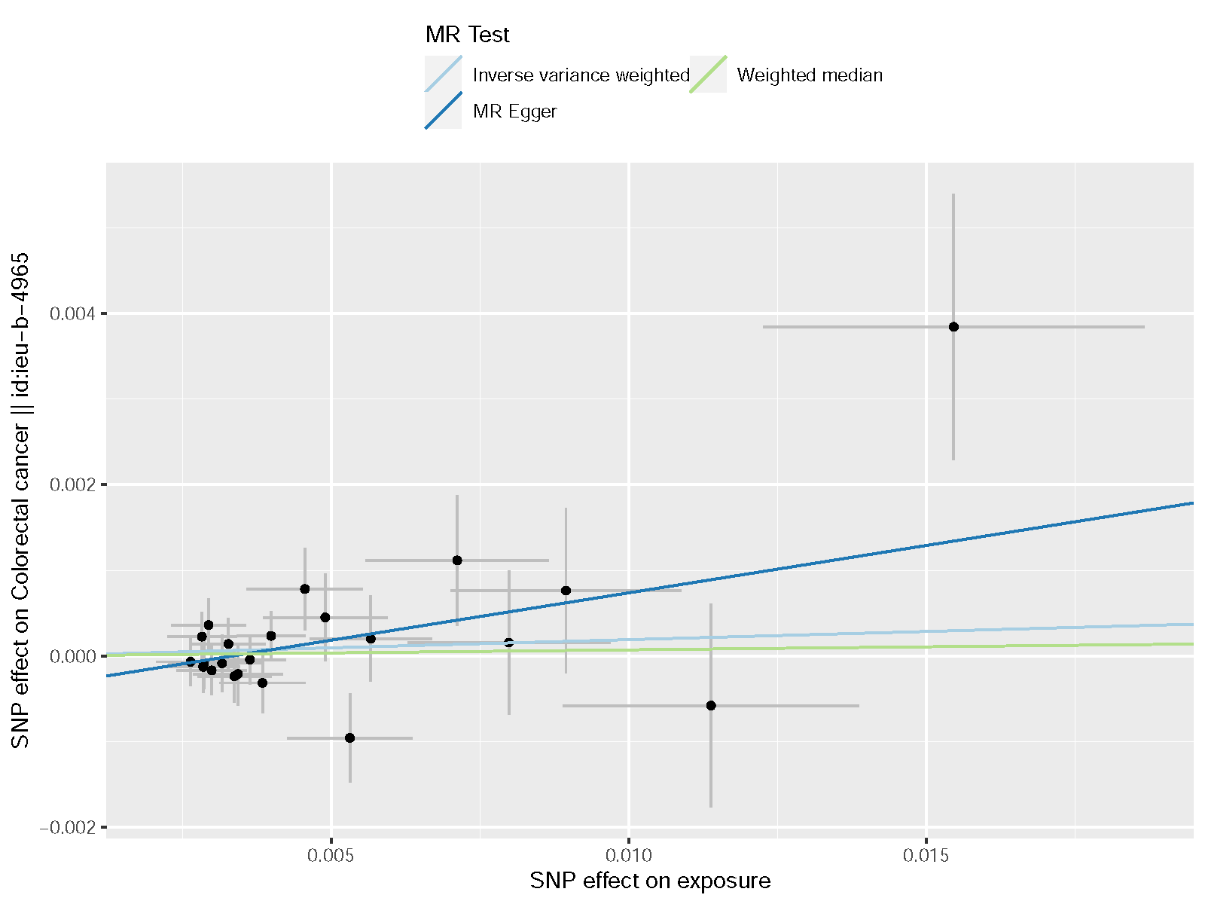

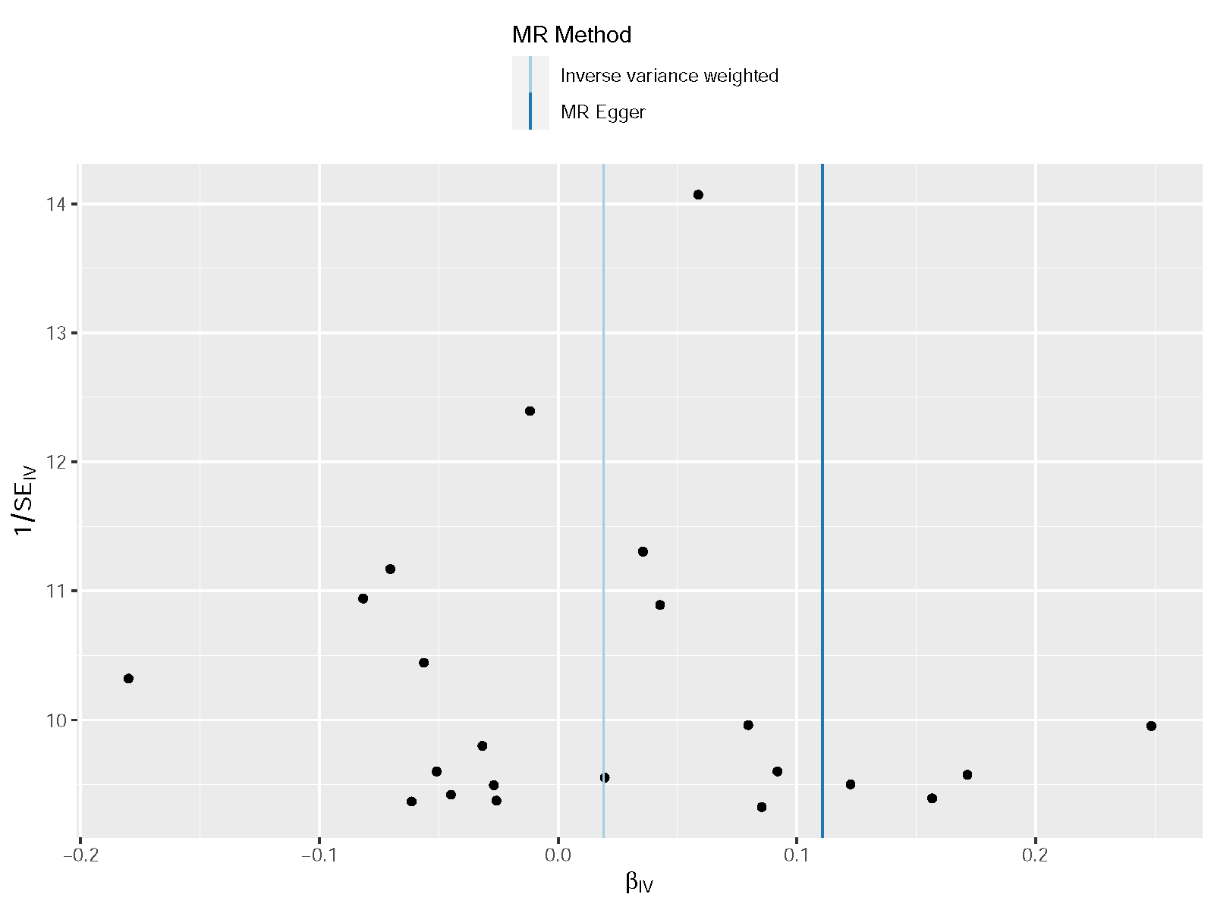

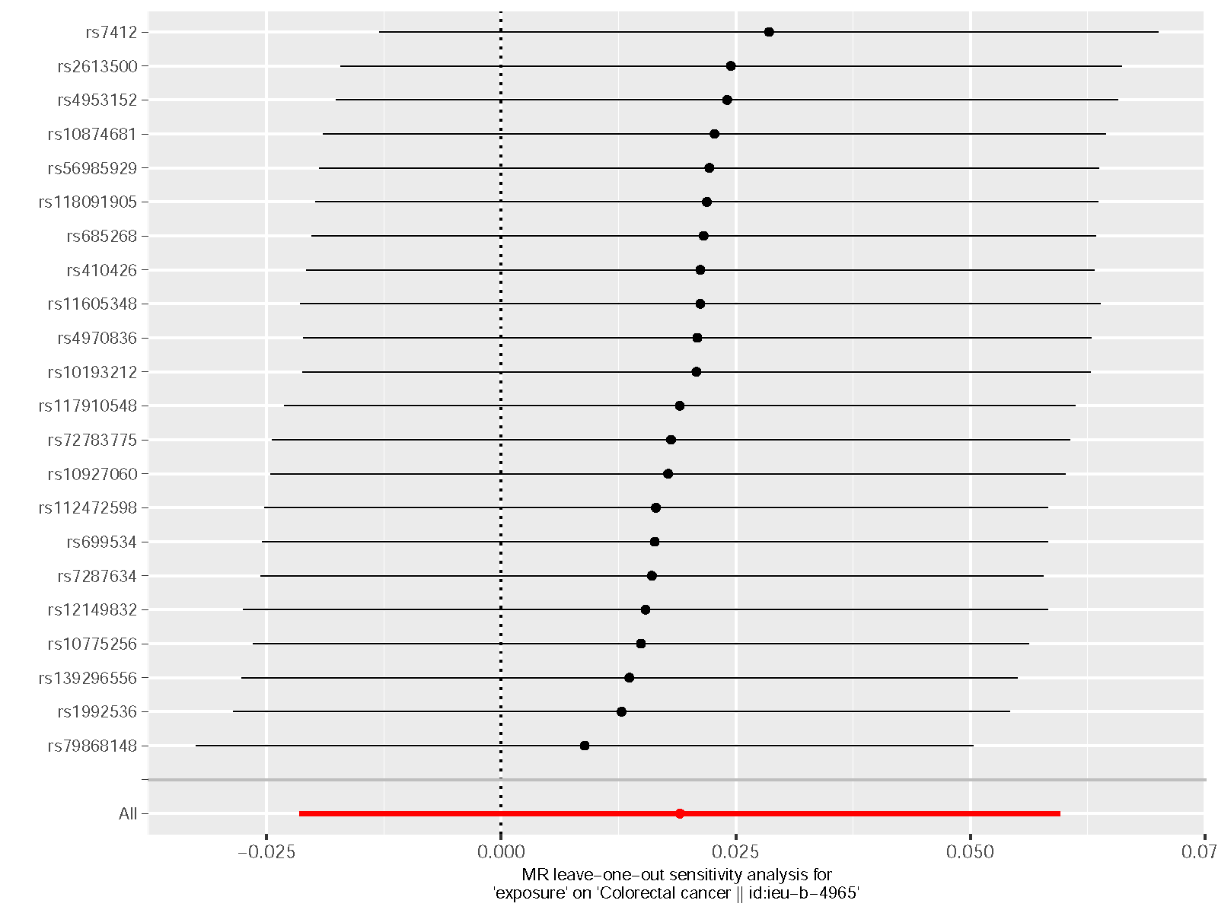


**C**

**B**

**A**

Figure S21.

A. Scatter plot of SNPs associated with Full cream milk and their risk of colorectal cancer. B. Funnel plot of SNPs associated with Full cream milk and their risk of colorectal cancer. C. Leave-one-out of SNPs associated with Full cream milk and their risk of colorectal cancer.


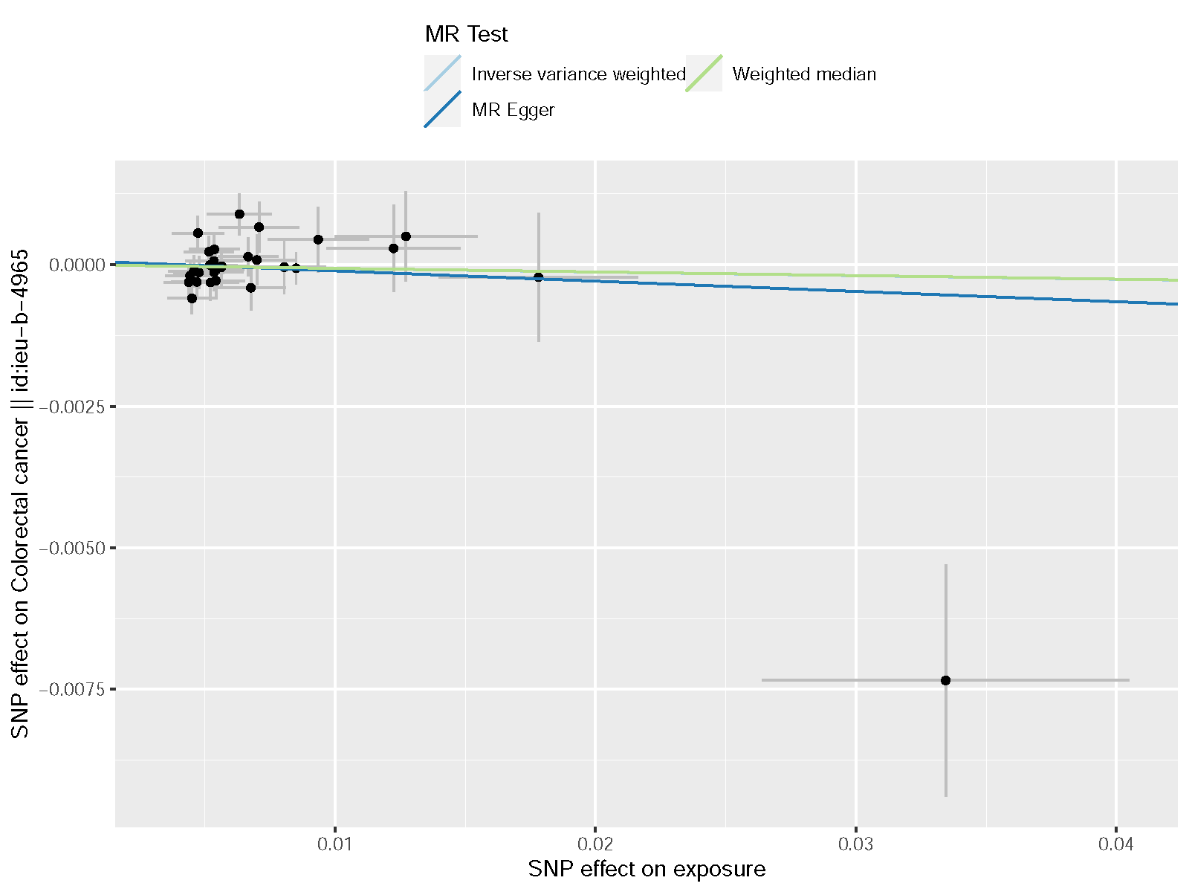

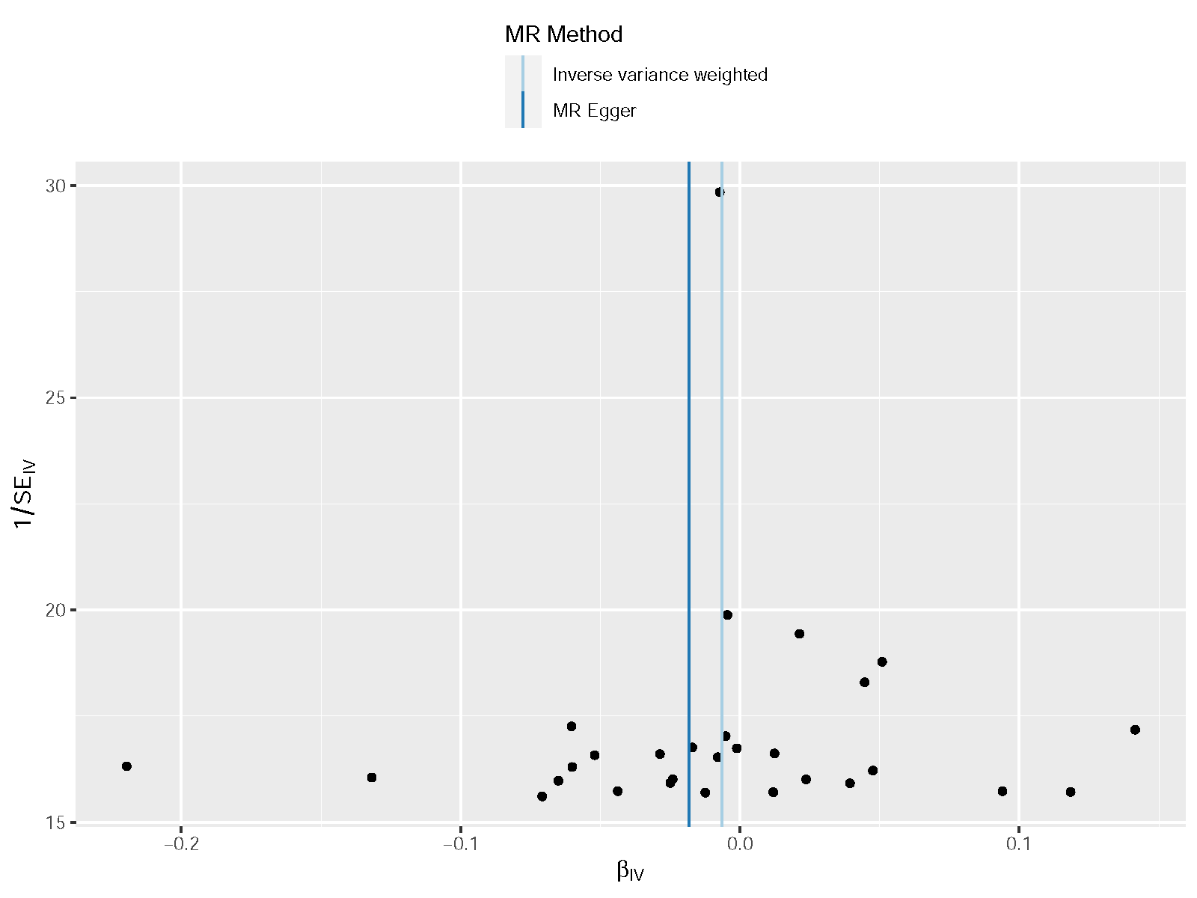

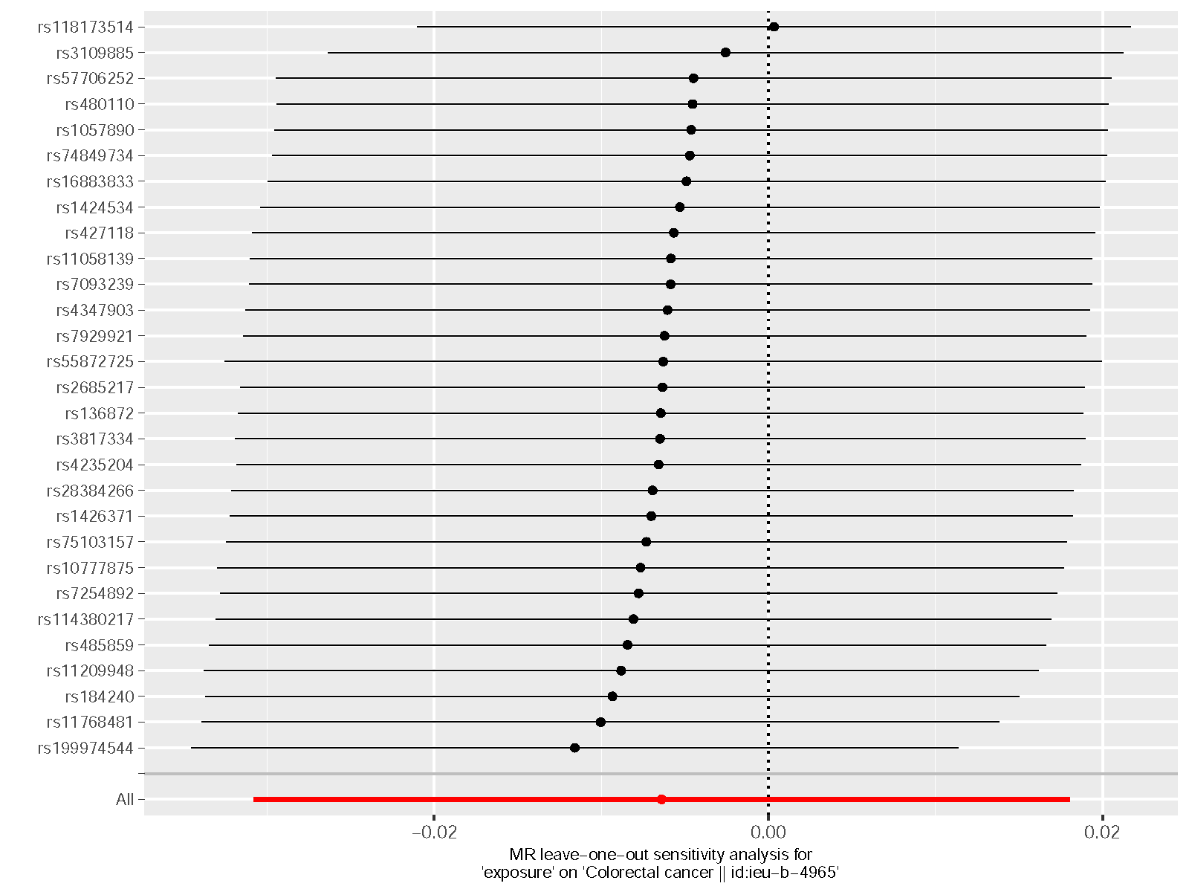


**A**

**C**

**B**

Figure S22.

A. Scatter plot of SNPs associated with Skimmed milk and their risk of colorectal cancer. B. Funnel plot of SNPs associated with Skimmed milk and their risk of colorectal cancer. C. Leave-one-out of SNPs associated with Skimmed milk and their risk of colorectal cancer.


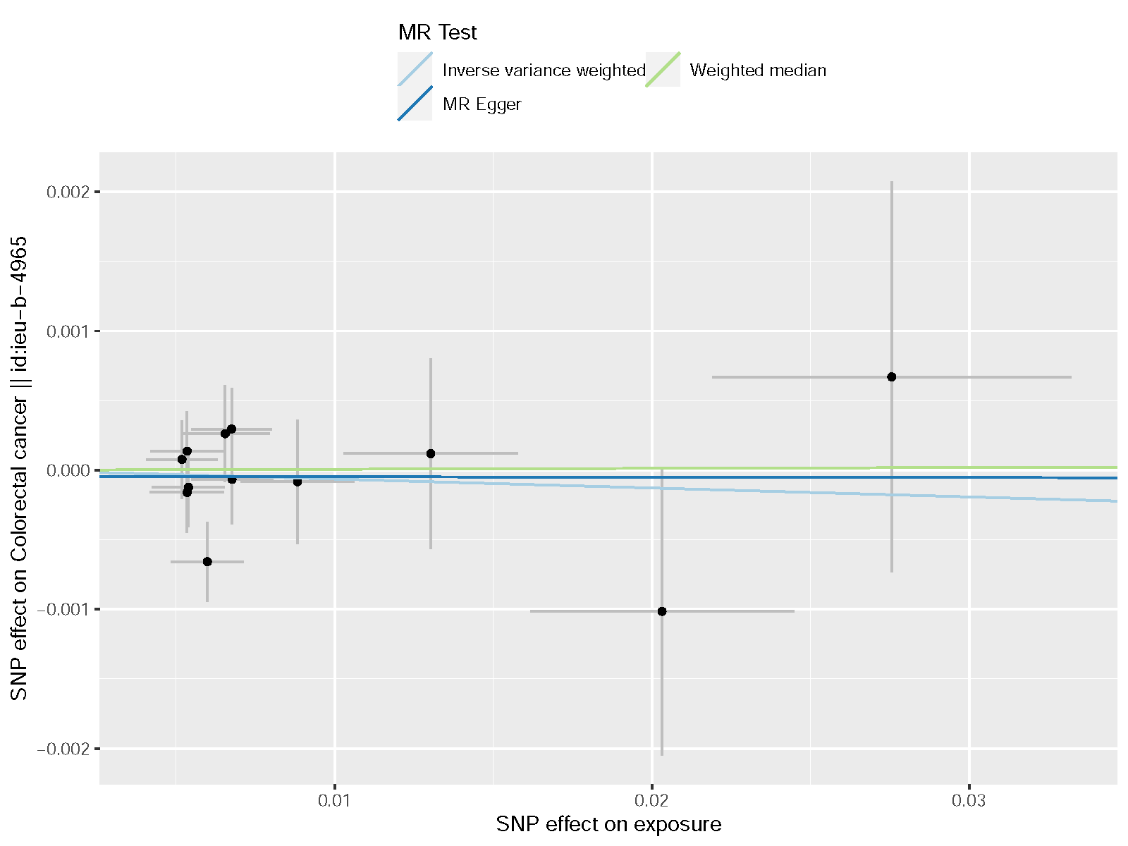

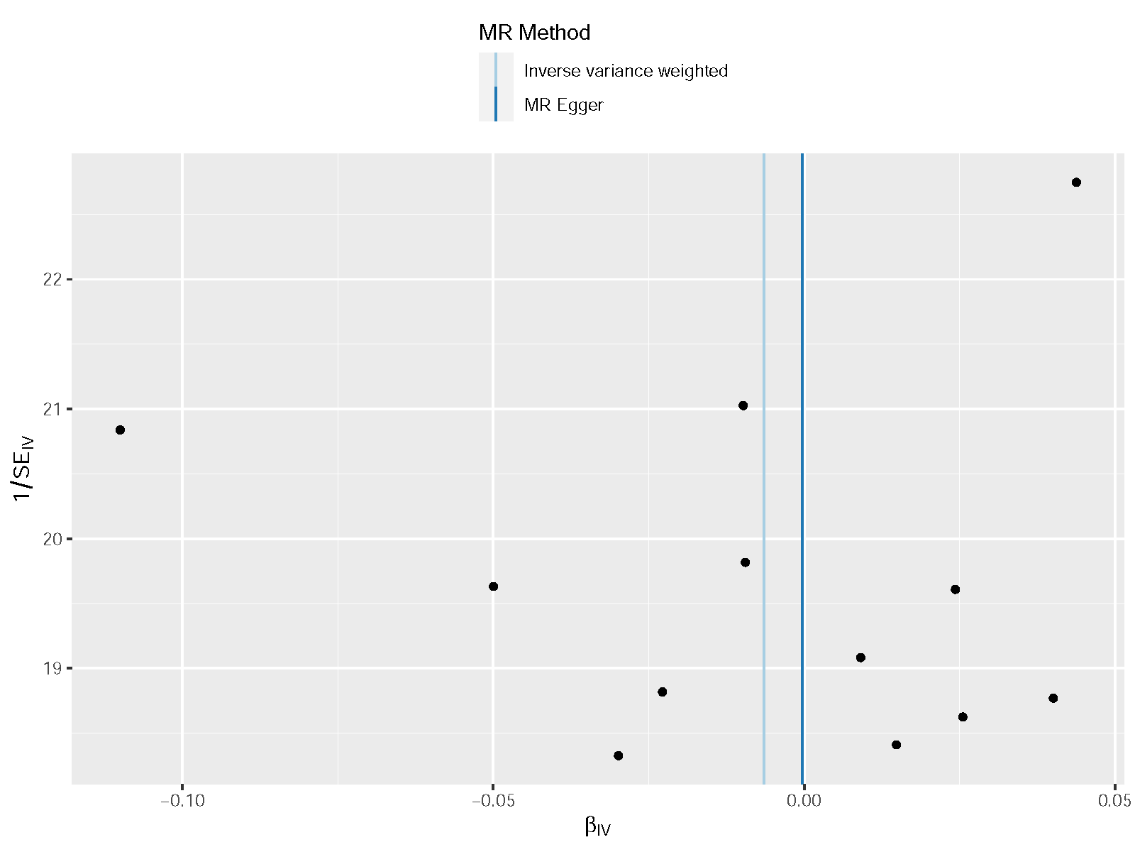

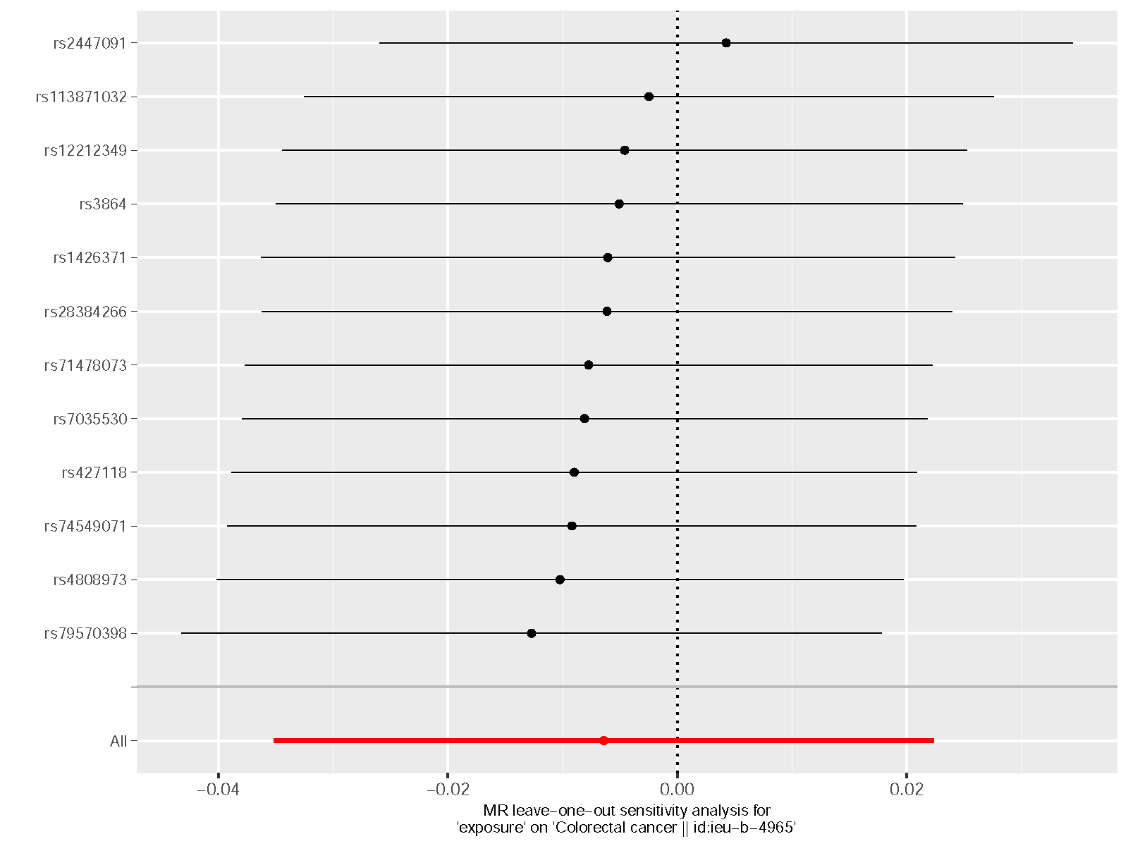


**C**

**A**

**B**

Figure S23.

A. Scatter plot of SNPs associated with Semi-skimmed milk and their risk of colorectal cancer. B. Funnel plot of SNPs associated with Semi-skimmed milk and their risk of colorectal cancer. C. Leave-one-out of SNPs associated with Semi-skimmed milk and their risk of colorectal cancer.


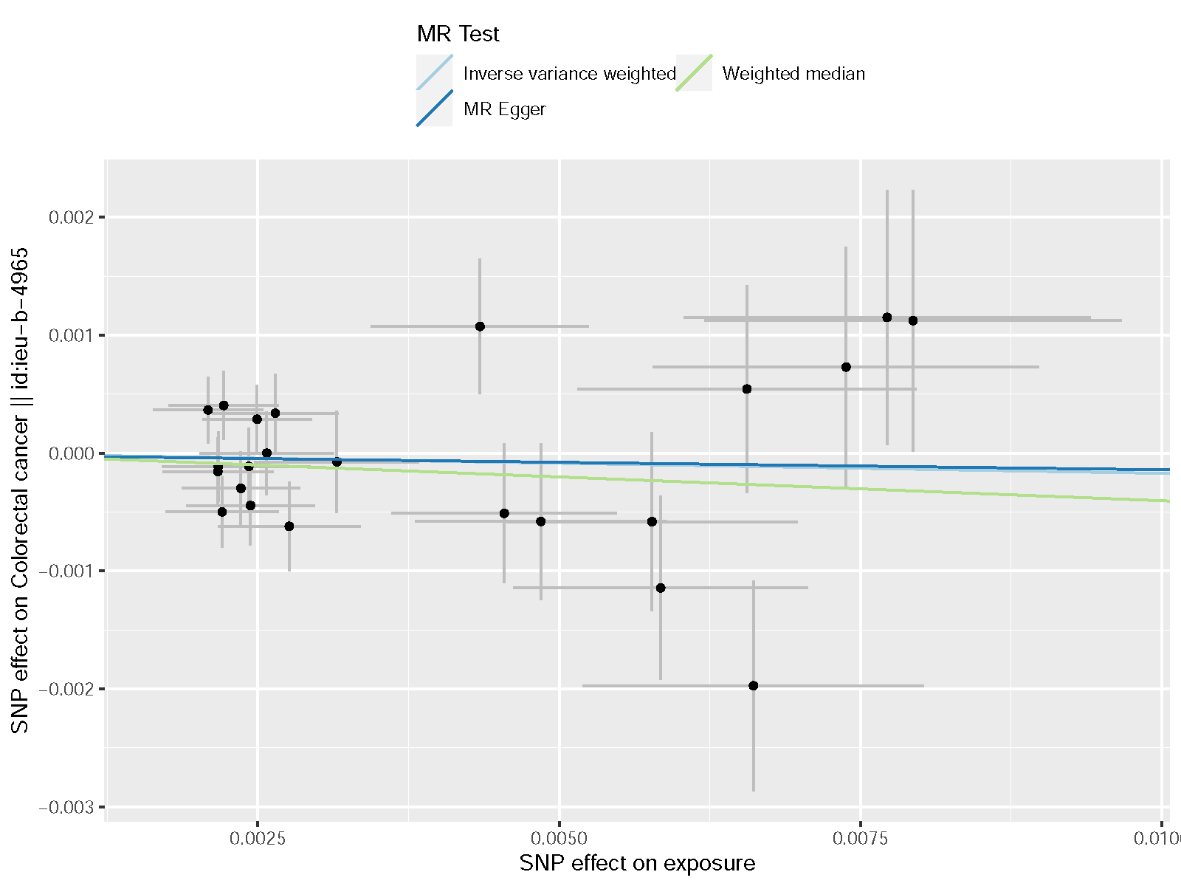

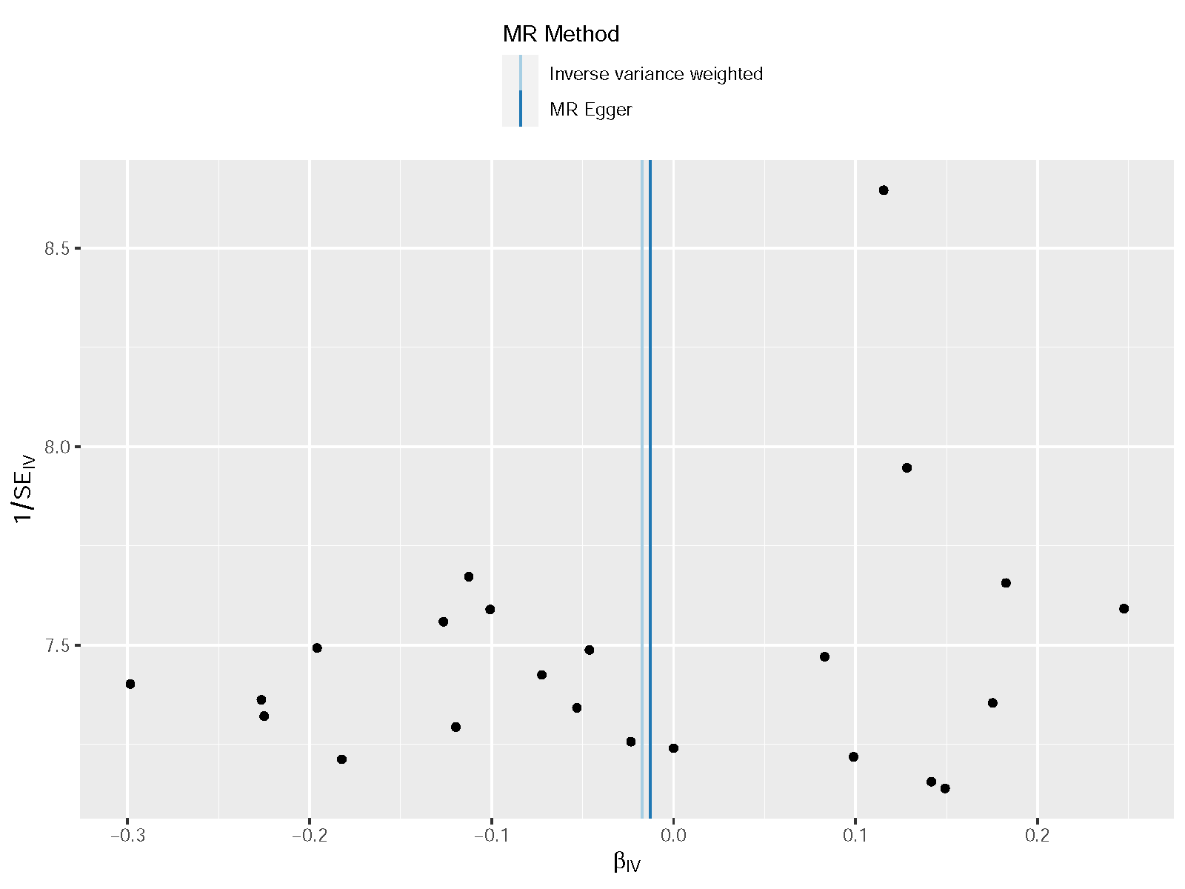

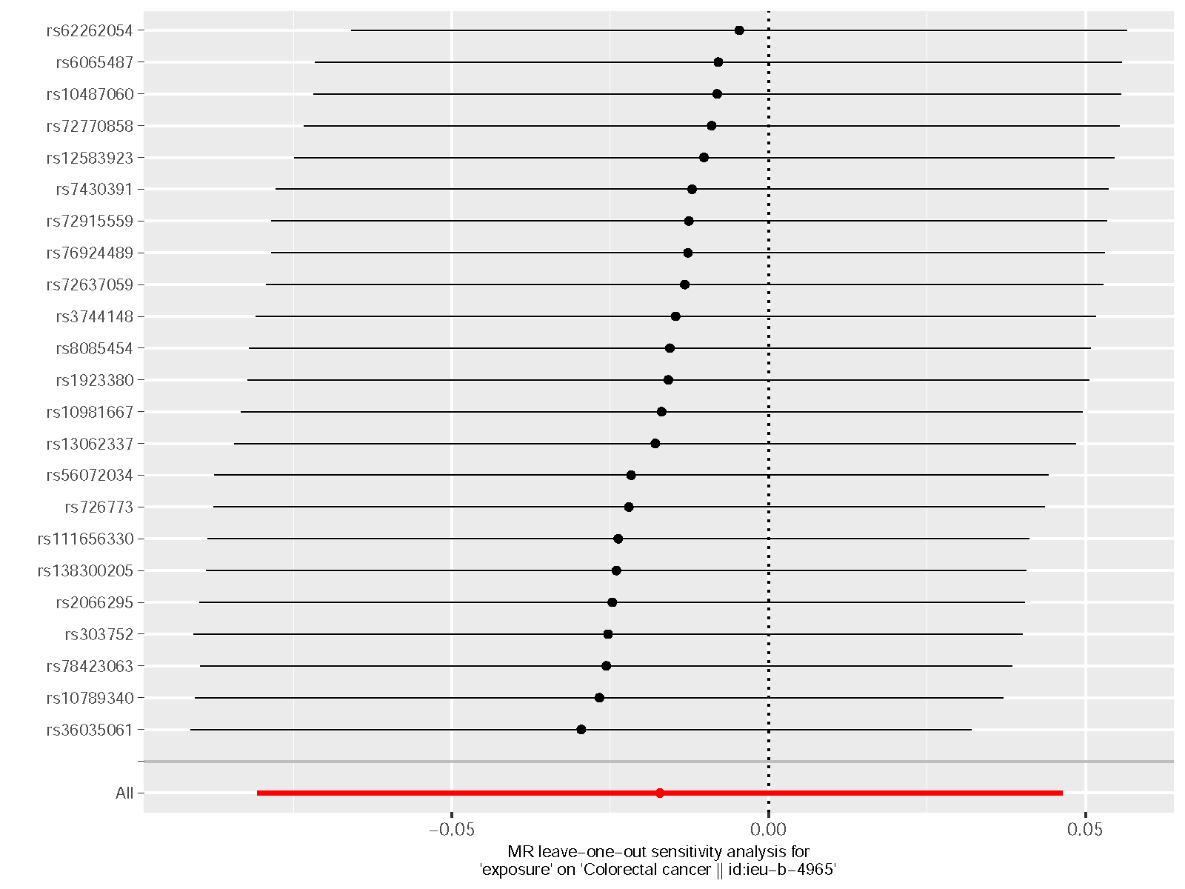


**A**

**B**

**C**

Figure S24.

A. Scatter plot of SNPs associated with Soya milk and their risk of colorectal cancer. B. Funnel plot of SNPs associated with Soya milk and their risk of colorectal cancer. C. Leave-one-out of SNPs associated with Soya milk and their risk of colorectal cancer.


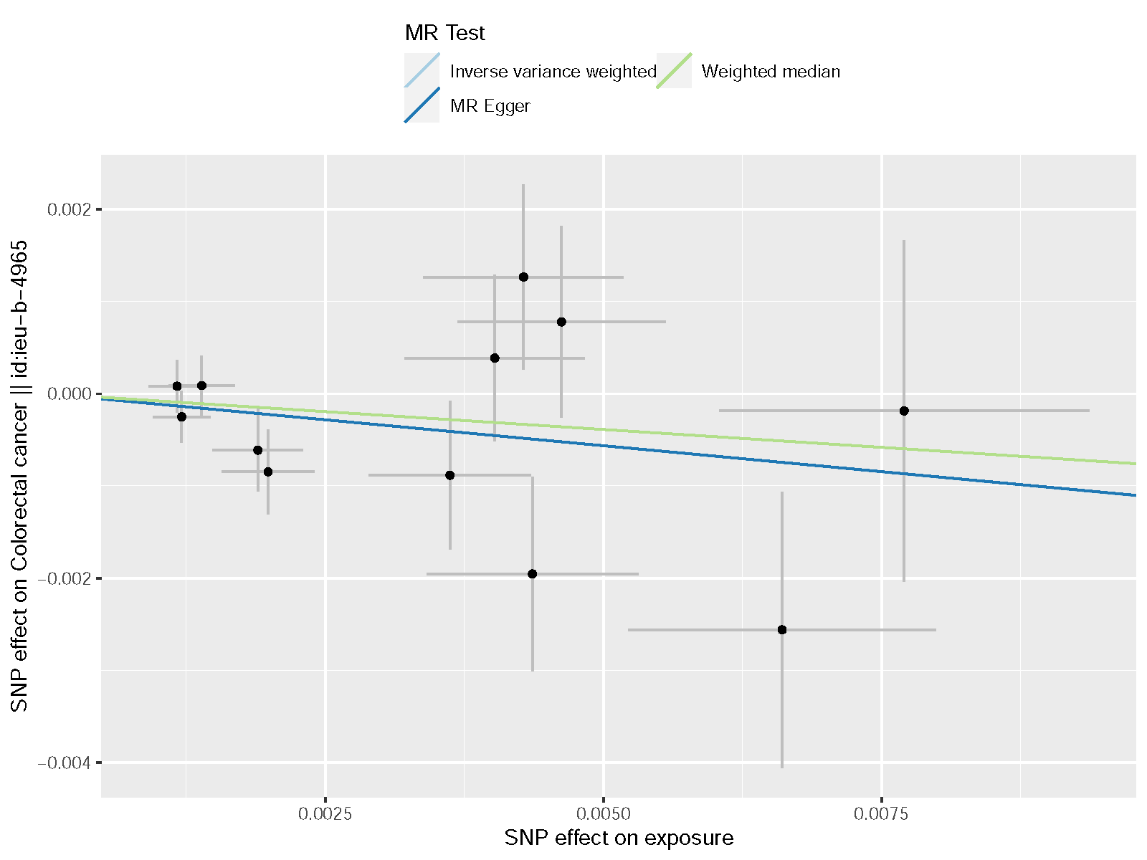

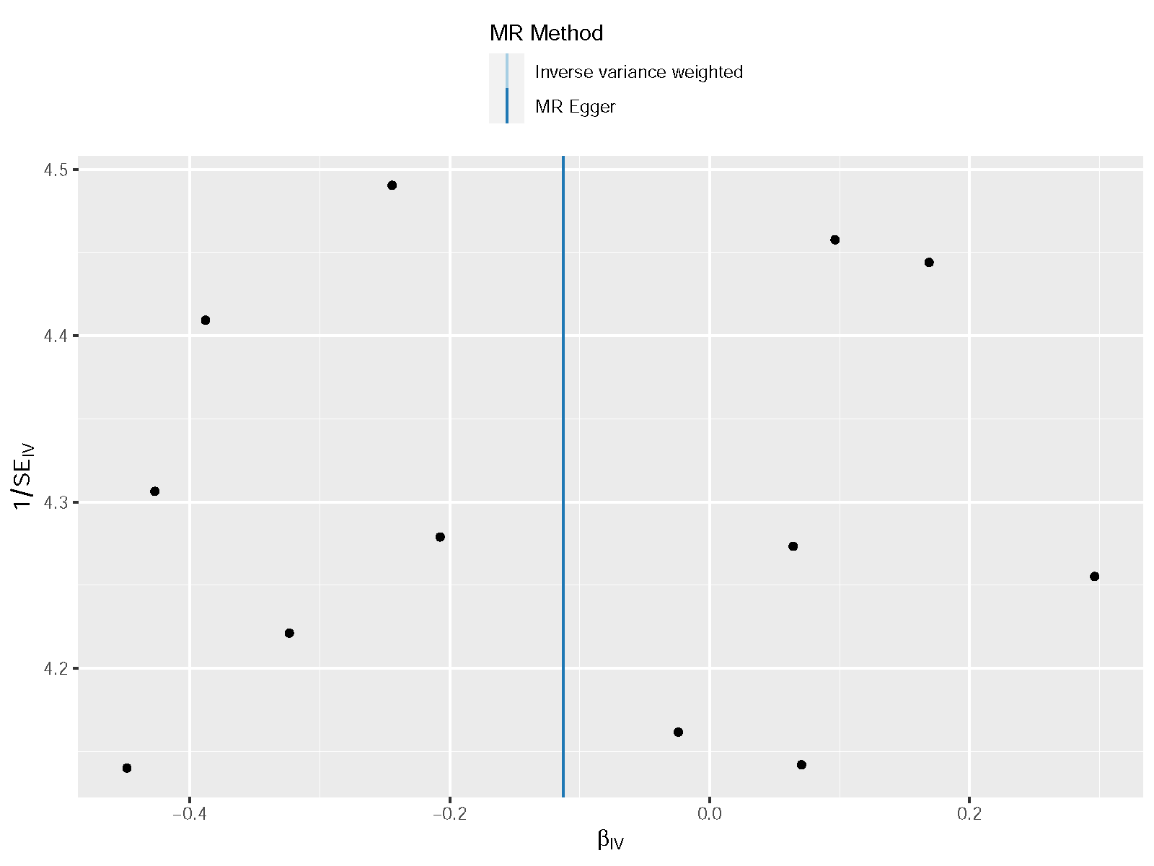

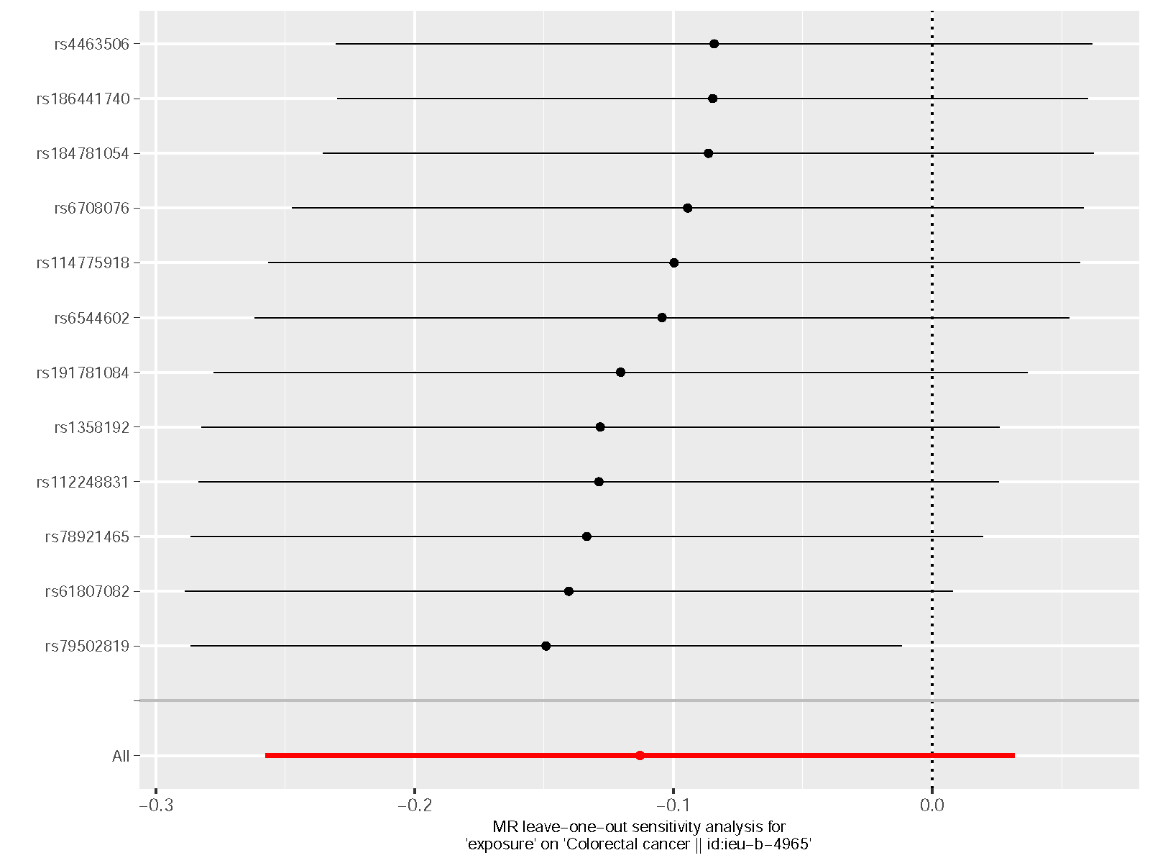


**C**

**B**

**A**

Figure S25.

A. Scatter plot of SNPs associated with Other type of milk and their risk of colorectal cancer. B. Funnel plot of SNPs associated with Other type of milk and their risk of colorectal cancer. C. Leave-one-out of SNPs associated with Other type of milk and their risk of colorectal cancer.
